# *Prosopis*: a global assessment of the biogeography, benefits, impacts and management of one of the world's worst woody invasive plant taxa

**DOI:** 10.1093/aobpla/plu027

**Published:** 2014-06-04

**Authors:** Ross T. Shackleton, David C. Le Maitre, Nick M. Pasiecznik, David M. Richardson

**Affiliations:** 1 Department of Botany and Zoology, Centre for Invasion Biology, Private Bag X1, Stellenbosch University, Matieland 7602, South Africa; 2 Natural Resources and the Environment, CSIR, P.O. Box 320, Stellenbosch 7599, South Africa; 3 Agroforestry Enterprises, Villebeuf, Cussy en Morvan 71550, France

**Keywords:** Classification and regression tree, distribution, global review, impacts, logistic regression, management, mesquite, tree invasions.

## Abstract

Invasive species cause ecological, economic and social impacts and are key drivers of global change. This is the case for the genus *Prosopis* (mesquite; Fabaceae) where several taxa are among the world's most damaging invasive species. Many contentious issues (‘conflicts of interest’) surround these taxa, and management interventions have not yet sustainably reduced the negative impacts. There is an urgent need to better understand the factors that drive invasions and shape management actions, and to compare the effectiveness of different management approaches. This paper presents a global review of *Prosopis*, focusing on its distribution, impacts, benefits and approaches to management. *Prosopis* was found to occur in a 129 countries globally and many more countries are climatically suitable. All areas with naturalized or invasive *Prosopis* species at present are suitable for more taxa and many Asian and Mediterranean countries with no records of *Prosopis* are bioclimatically suitable. Several *Prosopis* species have substantial impacts on biodiversity, ecosystem services, and local and regional economies in their native and even more so in their invasive ranges; others provide multiple benefits to local communities. Management efforts are underway in only a small part of the invaded range. Countries where more research has been done are more likely to implement formal management than those where little published research is available. Management strategies differ among countries; developed nations use mainly mechanical and chemical control whereas developing nations tend to apply control through utilization approaches. A range of countries are also using biological control. Key gaps in knowledge and promising options for management are highlighted.

## Abstract (precis)

Taxa from the genus *Prosopis* are widespread invasive aliens across the globe. Numerous species have contentious issues surrounding them as they provide both benefits and harm. *Prosopis* taxa are currently naturalised or invasive in 103 countries and are bioclimatically suitable for many more. There are numerous management practices available to control *Prosopis* invasions, each with their benefits and costs, however, in most areas management has had only limited success. There is need for more research to improve understanding and management success and for countries to develop strategic plants to guide managed in the future.

## Introduction

The increased movement of humans around the world has facilitated transportation of many species to environments far from their native ranges. This has been done purposefully—to introduce new crops and horticultural and forestry species—and accidentally, for example as weed seed in grain shipments (Mack 2003). These introductions have led to the rise of biological invasions that cause substantial ecological, social and economic impacts, and they are one of the key drivers of global change (Vitousek *et al*. 1997; Pimentel *et al*. 2000). However, many alien species have been embraced by humans and are crucial for local livelihoods and national economies through the goods and services they provide (Shackleton *et al*. 2007; Kull *et al*. 2011; van Wilgen *et al*. 2011).

It is important to understand the dynamics of invasive species to reduce their negative impacts and maximize their benefits, but frameworks linking theory and management for biological invasions are lacking (Hulme 2003; Wilson *et al*. 2014). Management is inefficient in many areas due to lack of knowledge on key aspects of the invasive species. It is crucial to understand the reasons for introductions, uses (benefits), costs, ecology and scales of invasions and to elucidate perceptions and potential contentious issues when creating sustainable management plans (Kull *et al*. 2011; van Wilgen and Richardson 2014; Wilson *et al*. 2014). This is true for invasive species in the genus *Prosopis*.

Taxa of *Prosopis* (mesquite; Fabaceae) occur in most of the world's hot arid and semi-arid regions as native or introduced species (Pasiecznik *et al*. 2001). The genus *Prosopis* as described by Burkart (1976) consists of 44 species. They have been introduced globally and have become naturalized or invasive in many places (Rejmánek and Richardson 2013). Several *Prosopis* species are also ‘weedy’ in parts of their native ranges (Pasiecznik *et al*. 2001). In this paper we define native species as those whose presence in an area is not attributable to introduction by humans (this includes species that have spread into areas without assistance from humans by overcoming biogeographic barriers). Alien taxa are those whose presence in an area is attributable to introduction by humans. Naturalized taxa are alien taxa that are self-sustaining. Invasive taxa are naturalized taxa that have spread substantially from introduction sites (further details in Pyšek *et al*. 2004). We define ‘weedy’ taxa as native taxa that have increased in abundance and/or geographic range in their native ranges.

Numerous *Prosopis* taxa are recognized as major invaders across large parts of the world (Pasiecznik *et al*. 2001; Brown *et al*. 2004). ‘*Prosopis*’ is listed as one of the 20 weeds of national significance in Australia and taxa in the genus are declared as major invasive species in Ethiopia, India, Kenya and South Africa, and Sudan is advocating for its eradication (FAO 2006; Australian Weeds Committee 2012; Low 2012; van Wilgen *et al*. 2012). Factors that make many *Prosopis* species successful invaders include the production of large numbers of seeds that remain viable for decades, rapid growth rates, an ability to coppice after damage (Felker 1979; Shiferaw *et al*. 2004), root systems that allow them to efficiently utilize both surface and ground water (to depths of >50 m) (Nilsen *et al*. 1983; Dzikiti *et al*. 2013), and allelopathic and allelochemical effects on other plant species (Elfadl and Luukkanen 2006). Many *Prosopis* species can also withstand climatic extremes such as very high temperatures and low rainfall, and they are not limited by alkaline, saline or unfertile soils (Pasiecznik *et al*. 2001; Shiferaw *et al*. 2004). Interspecific hybridization also enhances invasiveness in many introduced regions (Zimmermann 1991).

*Prosopis* invasions generate environmental, social and economic benefits as well as harm (Chikuni *et al*. 2004; Geesing *et al*. 2004; Wise *et al*. 2012). This has led to contentious issues surrounding the genus (Richardson 1998*b*; van Wilgen and Richardson 2014). Some advocates promote it as a ‘wonder plant’ while others call for its eradication, or contrast its positive and negative aspects, e.g. ‘Boon or bane’ (Tiwari 1999), ‘Pest or providence, weed or wonder tree?’ (Pasiecznik 1999), ‘Invasive weed or valuable forest resource?’ (Pasiecznik 2002). Contrasting views, contradictory perceptions and unclear policies are limiting options for constructive dialogue between different parties. This is exacerbated by problems in identifying and differentiating morphologically similar species, and by a general lack of knowledge on the distribution, scale of invasion, benefits, impacts and effective management approaches. Furthermore, many different approaches for managing *Prosopis* have been tried in different situations, without a thorough evaluation of the relative effectiveness of the methods. The Food and Agricultural Organization has called for a sound, unbiased global overview of *Prosopis* to act as a prerequisite for the holistic management of the genus (FAO 2006). Such reviews have been useful for guiding and prioritizing management and improving knowledge in other groups of woody invasive plants (Richardson and Rejmánek 2004, 2011; Kull *et al*. 2011; Wilson *et al*. 2011).

The aims of this paper are thus to (i) contrast benefits and costs of invasive *Prosopis*, (ii) update knowledge on *Prosopis* occurrence and introductions globally and highlight the potential range expansion of *Prosopis*, (iii) elucidate ecological, economic and social factors that shape attempts at managing *Prosopis*, (iv) compare and contrast the effectiveness of different management approaches in different regions, and (v) identify priorities for research and policy development. We review the literature and collate data from many sources. Details on the approach for the literature review, approaches used for statistical analyses and climate matching are provided in **Supporting Information**.

## Benefits and Costs

### Benefits, costs and invasiveness of different species

**Figure 1.**
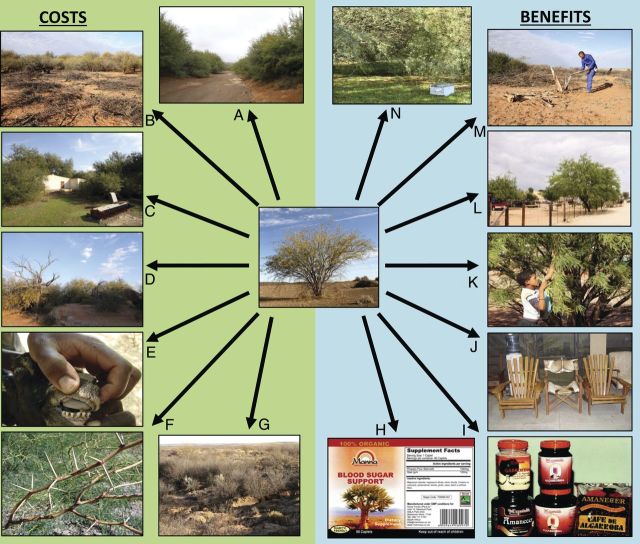
Costs and benefits of introduced *Prosopis* species: (A) invasive *Prosopis* stand altering hydrology in Loeriesfontein, South Africa; (B) cleared *Prosopis* in the foreground and uncleared in the background illustrating impenetrable thickets, loss of land, loss of grazing potential and the effort needed for its control in Kenhardt, South Africa; (C) loss of access to a barn and encroachment of fields in Calvinia, South Africa; (D) death of a native tree (*Searsia lancea*) due to competition from *Prosopis* in Kenhardt, South Africa; (E) effects of *Prosopis* pods on a goat's teeth in Kenya; (F) *Prosopis* thorns that cause tyre damage and injure humans and livestock; (G) *Prosopis* causing loss of topsoil and erosion in Prieska, South Africa; (H) ‘manna’—a blood sugar medicine made from *Prosopis* in South Africa (www.mannaplus.co.za); (I) food products made from *Prosopis* in Peru; (J) timber from *Prosopis* used to make furniture in Kenya; (K) a young boy collecting *Prosopis* pods to feed livestock in Askham, South Africa; (L) *Prosopis* used for shade and ornamentation in Askham, South Africa; (M) *Prosopis* used as a fuel in Kenhardt, South Africa; (N) a bee hive placed in an invasive *Prosopis* stand Calvinia, South Africa. Photos: S. Choge (J), G. Cruz (I), P. Manudu (E, F), R. Shackleton (A–D, G, K–N).

*Prosopis* provides benefits and generates costs which have led to contentious issues surrounding the genus (Fig. 1). The ‘usefulness’ of *Prosopis* has led to the large-scale introduction of five species in particular (*P. chilensis*, *P. glandulosa*, *P. juliflora*, *P. pallida* and *P. velutina*) and the subsequent naturalization and invasion of these taxa and their hybrids leading to the provision of benefits and costs in their new ranges **[see Supporting Information]**. Although *P. pallida* is invasive in many areas (Rejmánek and Richardson 2013), it appears to be less aggressive than some other species (Pasiecznik *et al*. 2006*a*, *b*).

Several species are also weedy and thus provide both benefits and costs in their native ranges (*P. affinis*, *P. caldenia*, *P. campestris*, *P. chilensis*, *P. cineraria*, *P. farcta*, *P. glandulosa*, *P. hassleri*, *P. humilis*, *P. juliflora*, *P. kuntzei*, *P. nigra*, *P. pubescens*, *P. ruscifolia*, *P. strombulifera*, *P. tamarugo*, *P. velutina*) **[see Supporting Information]**. At least 19 (invasive and weedy) of the 44 species in the genus are known to generate benefits and costs, with the rest being only beneficial. The invasiveness and potential negative impacts of many *Prosopis* species are still unknown as only a handful have been introduced.

### Uses/benefits

*Prosopis* species have been used for a variety of products for more than 5000 years in their native ranges (Pasiecznik *et al*. 2001). The numerous goods and services provided by *Prosopis* have led to global introductions and have made some species important for local communities. *Prosopis* is commonly used for fuel, fodder, windbreaks, shade, construction materials and soil stabilization through its invasive ranges in Africa and Asia (Pasiecznik *et al*. 2001; Wise *et al.* 2012). In some areas the benefits from *Prosopis* are, or were, regarded as a key income source for many households. In one village in Malawi, 44 % of people relied on *Prosopis* products as a primary or supplementary source of income (Chikuni *et al*. 2004). Communities in Kenya have benefited greatly from the sale of charcoal and *Prosopis* pods for fodder, boosting the local economy in some areas by US$1.5 million per year (Choge *et al*. 2012). In India, *Prosopis* provides up to 70 % of fuelwood needs for local households in some dry region villages (Pasiecznik *et al*. 2001).

Although utilization is most common in rural settings to sustain local livelihoods, *Prosopis* products are also exploited on a large scale by private companies. In South Africa, pods are collected to produce organic medicines (‘manna’) that are said to have properties that stabilize blood sugar levels in humans. This company is making profits of US$100 000 per annum and has the potential to increase profits 10-fold if the product is marketed internationally (Wise *et al*. 2012). A company in Brazil, Riocon, has an annual turnover of US$6 million a year from the sale of *Prosopis* pod flour for animal feeds (A. Davi, Ricocon, pers. comm.).

### Negative impacts/costs

**Figure 2.**
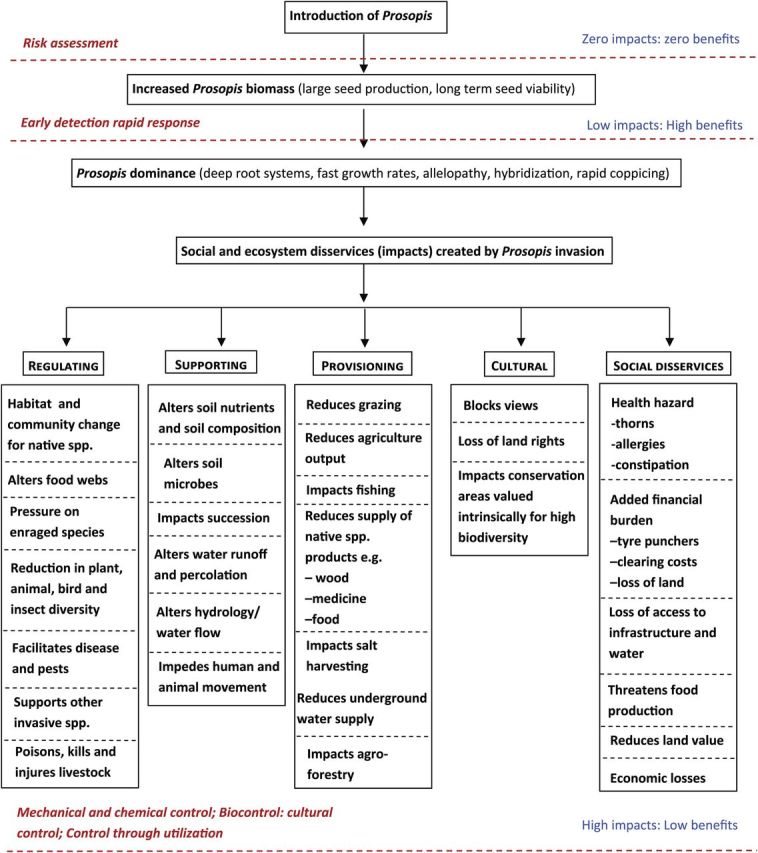
Cause-and-effect network diagram showing the negative effects of *Prosopis* invasions and management options that can be used to target each stage of invasion.

*Prosopis* invasions also have a variety of negative social, ecological and economic impacts (Figs 1 and 2). They alter ecosystem services such as water supply, hydrological functioning, grazing potential and soil quality (DeLoach 1984; Bedunah and Sosebee 1986; Archer 1989; Le Maitre *et al*. 2000; van Klinken *et al*. 2006; Ndhlovu *et al*. 2011; Nie *et al*. 2012; Dzikiti *et al*. 2013). Native biodiversity in many parts of the world has also been negatively impacted by invasive *Prosopis* species (Steenkamp and Chown 1996; Dean *et al*. 2002; El-Keblawy and Al-Rawai 2007; Belton 2008; Kaur *et al*. 2012).

Local communities in Kenya, Sudan, Eritrea, Malawi and Pakistan noted a range of negative consequences arising from invasive *Prosopis* (Choge *et al*. 2002; Chikuni *et al*. 2004; Mwangi and Swallow 2005; Laxén 2007; Bokrezion 2008; Kazmi *et al*. 2009)*.* These included effects on livestock health, *Prosopis* thorns causing tyre punctures and flesh wounds, dense thickets reducing access to water points, roads, infrastructure and agricultural and range lands, drying up of water sources, reducing natural forest cover and the services from these forests, as well as providing refuge for thieves.

In many parts of Africa *Prosopis* invasions are a leading cause of detrimental impacts on local community structure and functioning, leading to an increase in their vulnerability. This includes the potential loss of land rights for local livestock herders in Mali and violent conflict over limited natural resources between neighbouring communities in Ethiopia and Kenya (Centre for Sustainable Development Initiatives 2009; Djoudi *et al*. 2011; Stark *et al*. 2011). One Kenyan community has even taken the Food and Agricultural Organization (FAO) and the Kenyan government to court over the harm created by the introduction of *Prosopis* (Pasiecznik *et al*. 2006*a*).

Native weedy *Prosopis* taxa are also estimated to cause a loss of US$200–500 million per annum to the livestock industry in the USA (DeLoach 1984). In South Africa costs of managing *Prosopis* invasions are substantial, averaging $35.5 million per annum (van Wilgen *et al*. 2012).

### Benefits vs. costs and the dimensions of contentious issues

Perceptions on the benefits and costs of invasive alien species are strongly influenced by invasion abundance (Binggeli 2001; Shackleton *et al*. 2007). As abundance increases, associated costs rise and benefits fall due to issues such as resource accessibility (Wise *et al*. 2012). In India, *Prosopis* was initially seen as beneficial, but over time the negative consequences became more apparent, leading to increasingly negative perceptions of the plant from some quarters (Pasiecznik *et al*. 2001). A similar situation arose in Kenya where, as *Prosopis* became invasive, it was described as a ‘bad omen’ by some local people (Choge and Chikamai 2004) and more than 65 % of people in three villages mentioned that their lives would have been better off if *Prosopis* was never introduced (Maundu *et al*. 2009). In Sudan, over 90 % of livestock farmers viewed *Prosopis* as a problem as it became more widespread (Elsidig *et al*. 1998).

In many areas, invasive *Prosopis* trees do not sustain their full use potential due to intraspecific competition in dense stands which, generally, form over time. In such cases relatively few pods are produced for fodder and human consumption and dense invasive stands become impenetrable for humans and livestock making utilization of resources difficult (Chikuni *et al*. 2004; Mwangi and Swallow 2005). Wise *et al*. (2012) show that net economic benefits decrease as invasion densities increase in South Africa. They predict that the net cost of having *Prosopis* in the country will become negative in 4–20 years depending on future rates of spread. A framework by Shackleton *et al*. (2007) also shows that useful invasive aliens initially have high benefits, but as invasion densities increase, costs rise which lead to an increase in human vulnerability. This raises questions about the introduction of ‘miracle’ species in the past such as *Acacia*, *Leucaena* and *Prosopis* because the adverse impacts tend to exceed the benefits as the invasions progress, if left unmanaged (de Wit *et al*. 2001; Pasiecznik 2004; Wise *et al.* 2012; Low 2012), as well as the continued promotion of invasive alien species like *Prosopis* for biofuels today (Witt 2010; Naseeruddin *et al*. 2013).

The fact that the detrimental effects emerge only after invasions have reached unmanageable levels exacerbates contentious issues surrounding invasive species and may delay management decisions, in many cases restricting the implementation of effective management. There have also been conflicts of interest regarding which form of management to implement—how best to preserve, exploit and even enhance benefits while reducing negative impacts of *Prosopis* invasions (Zimmermann 1991).

## Introductions, Current and Potential Distribution of *Prosopis*

### Introductions

#### Dates and sources of introduction

**Figure 3.**
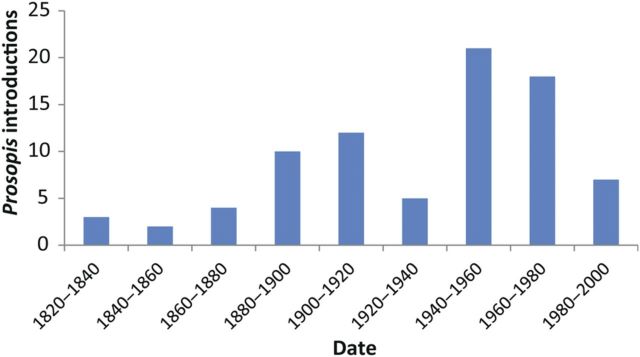
Time scale of all *Prosopis* introductions globally (*n* = 82 known species–country introduction dates).

Intercontinental introductions of *Prosopis* species have occurred over several centuries (Fig. 3). The first reports were of the introduction of *Prosopis* species from the Americas to Senegal in 1822, and to Australia, Hawaii, India, Philippines, South Africa, Sri Lanka and Sudan in the late 1800s and early 1900s (Pasiecznik *et al*. 2001). However, most of the widespread introductions were made into Africa and Asia between the 1970s and 1990s (Fig. 3) as part of reforestation programmes after major droughts in the Sahel. Many areas, notably India, South Africa and Sudan, have had multiple introductions over many decades. There is no evidence of new introductions post 1990, with the last recorded introductions being in Malawi and Burkina Faso in 1986 (Ræbild *et al*. 2003; Chikuni *et al*. 2004). There have, however, been recent calls for the introduction of known invasive *Prosopis* species to new locations. Hasan and Alam (2006) recommend that the planting of *Prosopis* would be beneficial to combat degradation in Bangladesh. Parvaresh (2011) proposed using *Prosopis* to stabilize dunes to protect important biologically diverse wetlands and mangrove forests in Iran. The promotion of biofuels could also lead to the spread of invasive woody species such as *Prosopis* (Witt 2010). There has also been extensive natural spread (commonly by means of flood water) and human-assisted spread (livestock trade) into new areas within countries where it is already naturalized and invasive (Van den Berg 2010).

Seed introductions have come both from native populations and from naturalized and invasive populations in countries where *Prosopis* was introduced previously. However, the original sources of seed and dates for introductions to many countries are poorly documented. Seed introduced to Hawaii came from a tree in France with a speculated provenance in Brazil (Pasiecznik *et al*. 2001) and *P. pallida* introduced to Australia came from Hawaii (Pasiecznik *et al*. 2001). South Africa had multiple introductions of many species and seed was most likely introduced from native ranges in Chile, Honduras, Mexico and USA (Zimmermann 1991). Seed from naturalized populations in South Africa was introduced into Egypt and seed introduced into Sudan came from South Africa and Egypt (Pasiecznik *et al*. 2001). The provenance of early *Prosopis* introductions to India is uncertain (likely Mexico or Jamaica); later introductions came from Argentina, Australia, Mexico, Peru and Uruguay (Pasiecznik *et al*. 2001).

#### Reasons for introduction

Most introductions of *Prosopis* were intentional, although there have been accidental cross-border introductions between neighbouring counties. *Prosopis* was introduced for many reasons: to provide fodder and shade in the arid areas of South Africa and Australia; for dune stabilization, afforestation and fuel wood supply in Sudan; for live fencing in Malawi; initially to rehabilitate old quarries and later for afforestation and the provision of fuelwood and fodder in Kenya; for fuelwood production and rehabilitating degraded soil in India; for local greening, ornamental cultivation and soil stabilization in many Middle Eastern countries; and for vegetation trials in Spain (Zimmermann 1991; Ghazanfar 1996; Pasiecznik *et al*. 2001; Choge *et al*. 2002; Chikuni *et al*. 2004; Elfadl and Luukkanen 2006; van Klinken *et al*. 2006; Laxén 2007; N. Pasiecznik and E. Peñalvo López, unpubl. res.). *Prosopis* was possibly first introduced unintentionally into Botswana, Nigeria and Yemen through livestock trading with neighbouring countries (Pasiecznik *et al*. 2001; Geesing *et al*. 2004).

#### Fate of introductions

**Figure 4.**
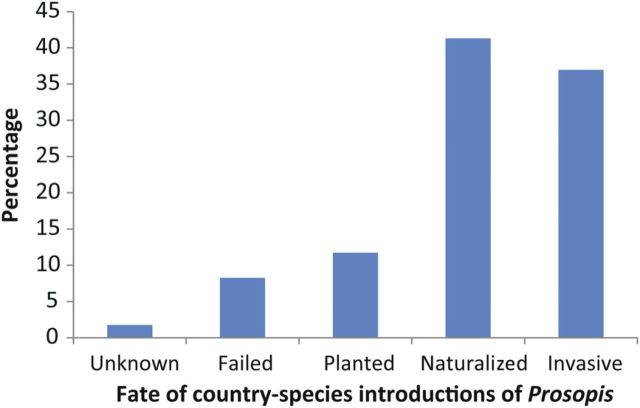
Classification of all records of introduced *Prosopis* species (236 introductions in 103 countries); classification of ‘naturalized’ and ‘invasive’ follows the criteria of Pyšek *et al*. (2004).

Of all the introductions of *Prosopis* species reviewed here, 79 % have led to naturalization, of which 38 % have become invasive (Fig. 4). No information on naturalization is available for 8 % of records, and 2 % of introductions are known to have failed (i.e. did not survive planting). Currently 12 % of introductions are only recorded as ‘planted’.

### Distribution

**Figure 5.**
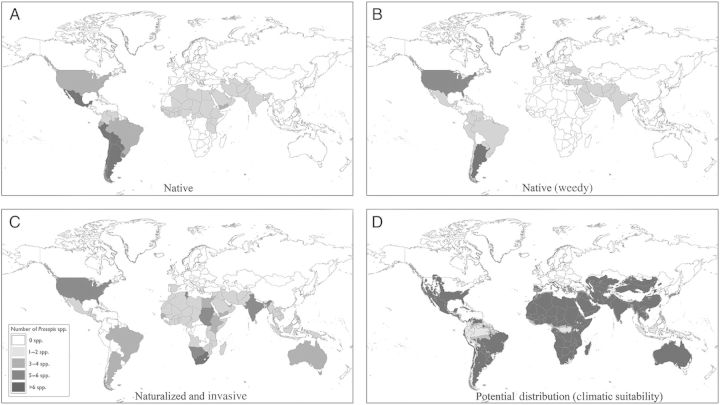
Global distribution of *Prosopis* species: (A) species diversity in countries with native taxa; (B) species diversity of taxa recognized as being weedy within their native ranges; (C) species richness of introduced *Prosopis* taxa that have either naturalized or become invasive (following the criteria of Pyšek *et al*. 2004); and (D) potential *Prosopis* species richness based on climatic suitability.

*Prosopis* currently occurs naturally or as an introduced species in at least 129 mainland and island countries and territories (Fig. 5; **see Supporting Information**). This includes the Caribbean islands (18) and mainland counties (19) in the Americas (excluding Canada, Suriname and Guyana), 40 countries in Africa, 26 in Asia, 4 in Europe, 24 island/atoll countries in the Pacific, Atlantic and Indian Oceans and Australia.

The last comprehensive global review of *Prosopis* distribution listed the presence of taxa in 93 mainland and island/attol countries (Pasiecznik *et al*. 2001). It is unlikely that *Prosopis* has been introduced into more places in the 13 years since that review was undertaken, but rather that data availability has increased in the intervening period or that there has been unintentional spread e.g. into Tanzania. Of the 129 countries, 26 have only native species, 64 have only introduced *Prosopis* species, and 39 have both native and introduced species. *Prosopis* is weedy in 38 % of countries where it occurs naturally and 38 % of species in the genus are currently categorized as weedy in their native ranges. The distribution and scale of invasions in countries with invasive *Prosopis* are not well known, with only 13 % of countries having detailed distribution or percentage cover data and not just records of occurrence.

### Potential distribution

Climate matching was used to assess areas of potential naturalization and invasion (Peel *et al*. 2007). We identified many regions that are climatically suitable for *Prosopis* where there are currently no records of any taxa (Fig. 5D).This includes countries in Europe (Greece, Italy, Portugal, Romania, etc.), South America (Guyana and Suriname), Asia (China, Japan, Nepal, South Korea, etc.) and numerous island/atoll countries and overseas territories (Comoros, Malta, Solomon Islands, Timor-Leste, etc.) (Fig. 5D; **Supporting Information**). All countries where at least one *Prosopis* species has been introduced and has established have the potential for the naturalization of additional *Prosopis* species. For example, there are currently seven naturalized and invasive *Prosopis* species recorded in South Africa, but the country is climatically suitable for many more species **[see Supporting Information]**. Maundu *et al*. (2009) also illustrated a high climatic suitability for *Prosopis* in southern and eastern Africa and showed that there are many areas that could have invasions but currently do not.

## Management of *Prosopis*

Naturalized and/or weedy *Prosopis* are reported in 112 countries. Currently 23 countries with weedy or invasive *Prosopis* (21 %) implement some form of formal management. No countries rely exclusively on biological control, 6 (26 %) use only mechanical or chemical control, 5 (22 %) use control through utilization and 11 (48 %) apply an integrated approach (three or more methods, including biological control, mechanical control, chemical control, control through utilization and cultural control) (Table 2).

Countries that use only chemical and mechanical control are mainly found in the Middle East and have small isolated invasions and are usually wealthier nations, whereas control through utilization is applied in poorer countries such as Kenya and Ethiopia. Biological control is driven by Australia and South Africa; however, there are also areas where ‘biological control agents’ are present but were not deliberately introduced, for example, Egypt (seed-feeding beetles—Coleoptera and Burchidae), Sudan and Yemen (*Algarobis prosopis*) (Delobel and Fediere 2002; Al-Shurai and Labrada 2006; Babiker 2006). In Yemen there is no evidence that the non-native *A. prosopis* feeds on the native *Prosopis cineraria* (Al-Shurai and Labrada 2006). There are concerns, however, that introduced insects could affect less invasive *P. pallida* populations in these areas that are utilized by local communities (Pasiecznik *et al*. 2006*a*, *b*). Another view is that any effect of such insects could improve the usefulness of less invasive taxa by reducing seed production and therefore potential invasiveness and could lead to less dense stands with larger trees and greater pod production (Zachariades *et al*. 2011).

Logistic regressions were run to determine which factors underpin whether a country has formal management of *Prosopis* taking place or not. The degree of understanding of *Prosopis* invasion impacts and ecology (besides residence time—the time since introduction) is a better determinant of whether or not a country will manage *Prosopis* than the socioeconomic conditions of the country (Table 1). The stepwise regression revealed that the level of impacts and overall knowledge on *Prosopis* invasions are key determinants of the presence of management within a country or not. Having knowledge on invasion potential/risk allows countries either to act timeously or to develop protocols to guide management based on an overall understanding of impacts, ecology, uses and special scales. Having a good understanding surrounding *Prosopis* invasions also helps to highlight the need for management, and subsequent management also stimulates the accumulation of further knowledge on invasions. Residence time might not be a significant predictor, because in wetter areas invasions tend to establish much faster than in drier areas (Table 1). Also, all countries have had *Prosopis* long enough to have naturalized and invasive populations (Zimmermann *et al*. 2006).

**Table 1.** Logistic regression highlighting the importance of different ecological, economical and social factors in determining management of *Prosopis* within a country.

Explanatory variable	Nagelkerke *R*^2^	Predictions—% correct	Wald stat	*P* value
No. of introduced *Prosopis* spp.	0.540	84.3	13.04	0.000
Source of introduction known	0.234	70.0	4.815	0.999
Time since introduction	0.009	47.1	0.275	0.626
Use level	0.103	67.1	4.19	0.242
Distribution and extent of *Prosopis* cover known	0.616	81.4	7.087	0.069
Level of *Prosopis* impacts	0.685	87.1	19.638	0.000
No. of publications relating to *Prosopis*	0.960	88.6	20.765	0.000
Overall knowledge of *Prosopis* invasions	0.686	92.9	16.993	0.005
GDP per capita	0.013	65.7	0.680	0.410
Human development index	0.041	68.6	0.324	0.569

Simple socioeconomic variables are poor predictors of the existence of management strategies as there is evidence of management in countries at all levels of development (Table 1). Many of the poorer countries receive foreign aid to implement and run management programmes, at least at the outset.

The findings of this review contradict previous publications that have argued that less developed countries have conducted less research and management of invasive alien species (McNeely *et al*. 2005; Pyšek *et al*. 2008; Nuñez and Pauchard 2009; McGeoch *et al*. 2010). Some developing countries are at the forefront of *Prosopis* research and management such as Kenya (control through utilization, social impacts) and South Africa (biological control), along with developed countries such as Australia and the USA. Witt (2010) noted that the prominence and severity of the impacts of *Prosopis* in developing countries has motivated this investment in research and understanding. However, there may be a lack of research for less prominent invasive alien species in poorer regions of the world.

**Figure 6.**
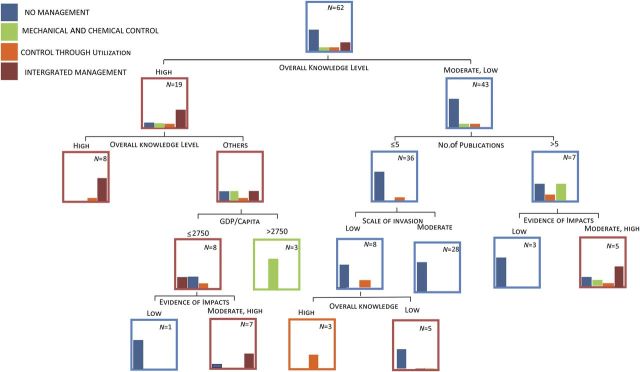
A classification and regression tree model using social, ecological and economic variables to explore the drivers of different types of *Prosopis* management globally.

The classification and regression model highlights the factors that underpin which management approaches counties are likely to adopt (Fig. 6). Similar to the regression output, the overall level of knowledge of *Prosopis* is an important factor when predicting which management approach or technique a country will adopt (Fig. 6). Countries with a good understanding of *Prosopis* based on the number of publications and the diversity of published materials have a higher chance of having some form of management, and in general this takes the form of integrated management. The level of development of a county, indicated by gross domestic product per capita, also influences the type of management approach a country is likely to adopt. Wealthier countries are more likely to implement mechanical and chemical control methods, which are the most costly but also currently the most effective options. Middle-income countries most commonly implement integrated management, whereas poor countries predominantly adopt control through utilization for managing *Prosopis*.

The advantages and disadvantages of these approaches differ (Table 2), and are closely linked to the costs of the control method. For example, countries with limited invasions are more likely to use mechanical and chemical control, whereas those with large-scale invasions are more likely to adopt an integrated approach, as purely mechanical and chemical control becomes too costly (van Klinken *et al*. 2006). Control through utilization aims to aid local development while simultaneously controlling *Prosopis* impacts and is therefore promoted in poorer parts of the world.

**Table 2.** A comparison of techniques for managing *Prosopis* and their advantages and disadvantages.

Control type	Advantages	Disadvantages
Biological control	• Relatively inexpensive once implemented • Works over large areas, including areas that are inaccessible for mechanical control • Minimal associated costs after biocontrol agent is released (monitoring is required)	• Biocontrol agents have not yet had substantial impacts on reducing stand density or extent of invasions and rates of spread in some areas such as South Africa but have been more successful in places like Australia • Initial research is expensive • Potential to spread across borders unintentionally • Inapplicable in areas where native *Prosopis* is weedy • Conflicts of interest around the use of biological control in areas where *Prosopis* invasion is seen as beneficial (e.g. South Africa, Kenya)
Mechanical control	• Efficient at removing *Prosopis* over large areas	• Labour and capital intensive
Chemical control	• Efficient at removing *Prosopis* over large areas	• Labour and capital intensive
Utilization	• Maximizes on benefits to be had from biological invasions • Promotes rural social–economical development • Reduces overexploitation of native spp. • Profits counteract management costs	• Encouraging utilization may create dependency on the species, thereby exacerbating conflicts of interest • Some areas have lower-value *Prosopis* spp. (more thorny, bitter pods, shrubby forms) making utilization more difficult • Many *Prosopis* invasions are in remote areas making large-scale utilization difficult
Cultural control/other control (e.g. fire, grazing and livestock transport management)	• Low costs • Can also prevent other types of degradation	• Requires people to change perceptions • Large-scale education programmers are needed • Does not always work for all *Prosopis* spp.—e.g. fire-tolerant hybrids • Not applicable in all areas, e.g. places with low biomass and fire-tolerant hybrids

### Contentious issues surrounding invasive *Prosopis* taxa and their management

The benefits and impacts and choice of different management approaches of *Prosopis* have led to contentious issues regarding management. Control through utilization is advocated by some as a management technique that enables benefit of invasive *Prosopis* to be utilized while simultaneously reducing the negative impacts of invasions and promoting local development (Choge and Chikamai 2004). However, many believe that this approach is inefficient at reducing invasions and leads to other problems such as dependencies (Table 2) (van Wilgen *et al*. 2011) and that other approaches such as chemical and mechanical clearing should be prioritized, although they are costly (Witt 2010). To date, there is no evidence of the success of control through utilization as a management technique (Table 2). The control through utilization approach is motivated around local development (which is needed) more so than managing invasions at large spatial and temporal scales.

There are conflicting views on best management approaches (eradication vs. control through utilization) in different villages in Kenya (Mwangi and Swallow 2005; Njoroge *et al*. 2012). Similar cases of contentious issues and conflicts of interest have been seen for other management approaches such as biological control. In South Africa only seed-feeding beetles were introduced so that neither the *Prosopis* trees themselves nor the production of pods would be harmed (Richardson 1998*a*)—even though better biological control agents have been identified that would harm trees and be more effective in reducing invasions (Zachariades *et al*. 2011).

### Case studies comparing different management approaches

Despite the growing body of research on management options for weedy and invasive *Prosopis* stands (van Klinken *et al*. 2006), there is an ongoing debate on how to effectively manage large-scale invasions. Different approaches are currently being used to manage *Prosopis*, each with their own set of advantages and disadvantages (Table 2). The following case studies were selected as being representative of different management strategies and also encompass the approaches most commonly employed in countries with different levels of socioeconomic development (developed—Australia; emerging economies—South Africa; developing—Kenya). The case studies are also characteristic of management strategies driven and implemented by different stakeholders, e.g. government driven with mainly private implementation (Australia), mainly government driven and implemented (South Africa) and government driven with some non-government organization (NGO) and international support (Kenya).

#### Australia

*Prosopis* has invaded over one million hectares and could potentially spread over 70 % of Australia's land area (Osmond 2003). *Prosopis* taxa are considered as one of the 20 worst invasives in Australia, and in accordance with the *Weeds Management Act* 2001, a strategic plan has been developed to guide management (Australian Weeds Committee 2012). *Prosopis* is a declared weed in all the mainland states and one territory in Australia and has been categorized in accordance with the threats it poses and the corresponding management responses that need to be implemented (van Klinken and Campbell 2009). This includes preventing introductions, trade, sale or movements of *Prosopis* taxa and the eradication of small populations and control of large populations (Australian Weeds Committee 2012). In general, most landowners use mechanical and chemical control measures to manage *Prosopis*. Although control and eradication programmes are primarily funded by the state, many private landowners also fund management operations. For example, in Queensland $A4 million was allocated for *Prosopis* management by the government, which was supplemented further by over $A600 000 by landholders between 1995 and 1999 and over $A2 million was spent on clearing between 2001 and 2005 (Martin and van Klinken 2006).

Control of *Prosopis* first started in 1954 at Mardie Station, Western Australia, and by 1962 a major reduction in *Prosopis* density had been achieved. Populations increased again when funding diminished, but in the mid-1970s the allocation of government funding led to substantial progress with clearing (van Klinken and Campbell 2009). In other areas of Western Australia control was improving, but after funding lapsed many infestations returned in the 1990s with the exception of some areas such as Yeeda Station where control had been successful due to annual monitoring and clearing (van Klinken and Campbell 2009). In Queensland substantial funding was invested for clearing in the area around Comongin Station, and by 2005 over 4000 ha of dense *Prosopis* stands had been removed (van Klinken and Campbell 2009). In northern Queensland research concluded that eradication was feasible in the region and significant steps have been made towards this goal (van Klinken and Campbell 2009). New South Wales and South Australia have similar examples of good control efforts and others that have had limited success due to a lapse in control and monitoring (van Klinken and Campbell 2009).

Four biological control agents have been released in Australia: *Algarobius bottimeri* and *A. prosopis* (seed-feeding bruchids), *Evippe* species (a leaf-tying moth) and *Prosopidopsylla flava* (a sap sucker) (van Klinken *et al*. 2003; van Klinken 2012). Two have established widely (*A. prosopis*, *Evippe* species), and the latter has had noticeable impacts on *Prosopis* populations through reducing long-term growth rates (van Klinken 2012). Biological control in Australia has been more successful than in other places like South Africa and the benefit-to-cost ratios are positive (0.5), with expectations to increase in the future (Page and Lacey 2006). The release of more agents is recommended to further improve control (van Klinken *et al*. 2003; van Klinken 2012).

Experiments have shown that some species are highly fire tolerant (especially the hybrids), which reduces the potential for using fire as a control method in many areas (van Klinken *et al*. 2006). Grazing control has also been advised to help prevent establishment and further spread of *Prosopis* (Csurhes 1996), although this approach has had limited success in Argentina and the USA (Dussart *et al*. 1998; Brown and Archer 1989). There are also regulations on the transport of livestock in areas infested with *Prosopis* to prevent its spread and accidental introduction elsewhere in Australia (Australian Weeds Committee 2012). Management policy is backed up by good legislation; Australia is one of two countries with a national management strategy. The government has also published many easily accessible documents on *Prosopis* management methods to inform landowners on control measures, and the *Prosopis* strategic plan places a lot of emphasis on educating and making stakeholders aware of *Prosopis* invasions and how to manage them (Australian Weeds Committee 2012). There have been rewarding examples of control success (van Klinken and Campbell 2009); however, *Prosopis* populations continue to spread in many areas and further management is needed.

#### South Africa

*Prosopis* invasions in South Africa cover an estimated 1.8 million hectares, and are increasing at 8 % per annum (Versfeld *et al*. 1998; Van den Berg 2010). They have the potential to invade between 5 and 32 million hectares of South Africa based on climatic suitability—about a third of the area of the country (Rouget *et al*. 2004). *Prosopis* is declared as a category 2 invasive alien species because it provides benefits and causes harm; this status means that it is legal to grow *Prosopis* in demarcated areas once a permit has been issued. A combination of mechanical, chemical and biological control methods is used to control *Prosopis*, mainly by the government-managed Working for Water programme. Three seed-feeding beetles (*A. prosopis*, *A. bottimeri* and *Neltumius arizonensis*) were introduced as biological control agents to try and reduce spread while maintaining its benefits (Zimmermann 1991; Coetzer and Hoffmann 1997). *Neltumius arizonensis* failed to establish (Zachariades *et al*. 2011). Although biological control is considered the most cost-effective way of managing large-scale invasions of many species, there are many cases where the agents fail to make a significant impact and *Prosopis* is one of them (van Wilgen *et al*. 2012). The overall return on investment is low compared with biological control programmes for *Opuntia* species and Australian *Acacia* species in South Africa (van Wilgen *et al*. 2012). There is potential to release more agents, such as the *Evippe* species which is already successful in Australia (see above), should the contentious issues surrounding the benefits and costs of *Prosopis* be resolved (Zachariades *et al*. 2011). *Prosopis* cover increased by ∼35 % between 1996 and 2008, despite the expenditure of R435.5 million (US$42.7 million) on control over this period. Only 15 100 ha were cleared using mechanical and chemical control with this substantial budget (van Wilgen *et al*. 2012), which makes the cost/ha very expensive (US$2828). The limited success to date may be due to lack of a management strategy and of prioritization of management projects (Forsyth *et al*. 2012). There is a need for researchers, managers and policy-makers to agree on new strategies for prioritizing areas for interventions to curb the spread of *Prosopis* and to ensure that the limited resources are used effectively (Forsyth *et al*. 2012). There have been some attempts at controlling *Prosopis* through utilization, but they had no noticeable impacts on invasions, and these initiatives failed as input and transport costs were too high and financial returns were low (Zimmermann *et al*. 2006). South Africa also has many particularly aggressive hybrids that form dense shrub-dominated stands, which makes the utilization approach difficult (Zimmermann *et al*. 2006).

#### Kenya

*Prosopis* is estimated to have invaded one million hectares and has the potential to invade nearly half of Kenya's surface (Maundu *et al*. 2009; Witt 2010). It was declared a noxious weed in 2008 (Low 2012). Biological and mechanical control was initially proposed as the management approach to combat *Prosopis* invasions, but the government later opted for a control-by-utilization approach (FAO 2006; Pasiecznik and Felker 2006). The FAO, with support from several NGOs, initiated programmes to manage *Prosopis* through utilization. These efforts were continued by the government's forestry department and forestry research organization (KEFRI) following the end of these projects. Considerable time and effort was taken to build capacity, formulate good policies and educate communities to utilize the goods and services from *Prosopis* (Pasiecznik *et al*. 2006*a*). For example, small-scale utilization projects were established and a cookbook using *Prosopis* flour was created and supplied to communities to promote its use (Choge *et al*. 2006; Pasiecznik *et al*. 2006*a*). Although initial costs for training and purchasing appropriate small-scale processing machinery are high, they are considered to be lower than other control approaches (Pasiecznik *et al*. 2006*a*). In 2002, trade in *Prosopis* goods and services was worth US$2122 per household per year in some villages (Choge *et al*. 2002). Ten years later, trade in *Prosopis* products in four selected areas was estimated to exceed US$1.5 million (Choge *et al*. 2012). Each tonne of pods that are collected and milled into flour is estimated to remove approximately two million viable seeds (Pasiecznik *et al*. 2006*a*). Changes in legislation, and the promotion of *Prosopis* use, helped drive the substantial rise in use and led to 100 % of the locals in one village supporting control through utilization as the most preferred management method to adopt in Kenya (Njoroge *et al*. 2012). However, in other villages 85–90 % of people surveyed considered complete eradication of *Prosopis* to be the best option (Mwangi and Swallow 2005). There are still, however, contentious issues surrounding the benefits and costs of the species and management approaches in Kenya (Pasiecznik *et al*. 2006*a*). There are many publications on the profits that are being made through utilization, but there is no evidence that these utilization programmes have contained or reduced the extent of *Prosopis* invasions. There is, therefore, a need for further investigation of the successes and failure of control through utilization programmes (Geesing *et al*. 2004). A common problem with trying to promote *Prosopis* utilization is that it is seen as an inferior resource in many communities, with people preferring to use native species (Geesing *et al*. 2004). Recently, a new utilization approach to increase invasive *Prosopis* use has been adopted in Kenya—a power station (based on technology from India) is currently being built in the Kenyan Rift Valley which aims to produce electricity for the local area from burning *Prosopis* biomass (S. Choge, pers. comm.).

### Research and management needs

**Figure 7.**
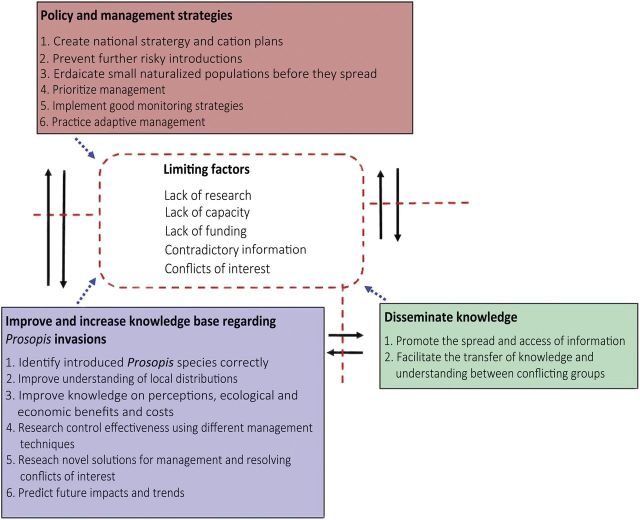
Requirements for research and management needs regarding *Prosopis* and factors limiting success.

This section highlights key management and research issues that need to be addressed to improve *Prosopis* control and the factors that currently constrain progress in these areas (Fig. 7). There is a great need for countries to develop national and even regional strategies, to provide guidelines for research and management in a targeted way, as each country has unique requirements and needs. Australia and Ascension Island are the only counties/territories to have strategic plans for *Prosopis* management and countries with long-standing *Prosopis* control programmes such as South Africa and Kenya still do not*.* Some broad-scale factors that need to be considered are suggested below.

#### Policy and management

National strategies and management/action plans need to be created and adopted to guide the coordinated control of *Prosopis* (Fig. 7)*.* Such national strategies and plans are important to set up frameworks on how to guide *Prosopis* management and research*.* Numerous organizations and national governments globally have undertaken projects to control *Prosopis*, and planning and prioritization from the outset would ensure greater success. Country-specific strategic plans need to be created as there are large differences in invasion rates and scales and socio-economic situations within different areas of the world.

Introductions of known invasive *Prosopis* species to climatically suitable countries where it does not already exist should be undertaken such as in China, European countries along the Mediterranean and North East Asia, and spread of *Prosopis* into new areas within countries where it is invasive should be prevented. Risk assessments for purposeful introductions need to be conducted in the future. Pathways of accidental introductions between neighbouring countries and into new areas in countries with invasive *Prosopis* need to be managed. This could include regulations on livestock and fodder transport which is currently implemented in Australia (Australian Weeds Committee 2012). This is done by holding livestock in feed lots for a week before they are transported to ensure that all *Prosopis* seeds have excreted.

Countries need to eradicate small naturalized populations before they become invasive. Early detection and rapid response is a cost-effective way of preventing invasive species from getting out of hand and causing devastating, irreversible impacts in the future. For example, in Spain, *Prosopis* has started to show signs of naturalization at a single location where it was planted for experiments and eradication attempts now would be most cost effective in the long run (N. Pasiecznik and E. Peñalvo López, unpubl. res.).

There is also an urgent need for managers and researchers to monitor the effectiveness of control measures. Adaptive management needs to be promoted and applied for controlling *Prosopis* invasions where operational success is so far limited, so that the causes of the failures can be identified and addressed to improve overall control. Managers and researchers need to collaborate in research to design from the outset successful adaptive management strategies to be implemented.

#### Improve knowledge

There are many research questions regarding *Prosopis* invasions in many parts of the world that need to be answered to improve management (Fig. 7).

These include correctly identifying *Prosopis* species present and gaining consensus on the status introduced and weedy species (e.g. following the criteria proposed by Pyšek *et al*. 2013). There have been numerous misidentifications of introduced *Prosopis* species, especially in Africa. This has caused much taxonomic confusion and contradictions between different sources of information that are only starting to be clarified. There are also hybridized populations in many areas where *Prosopis* has been introduced, further hindering identification (Zimmermann 1991. It was recently recognized that *P. pallida*, which was seen as not being as invasive as other species, is more widespread than originally thought as it was misidentified as *P. juliflora* in Africa (Pasiecznik *et al*. 2006*b*). Most species introduced to Africa were described as *P. chilensis*, but this is not the case, and accurate species lists are not available for many African countries such as Angola. Molecular methods are useful for clarifying taxonomic issues, especially in areas where hybridization has taken place. It is important to know which taxa are present for management, e.g. when looking for biological control agents and understanding ecology and rates of spread (Pyšek *et al*. 2013).

There is a need to improve the understanding of *Prosopis* distribution and population sizes in introduced ranges to guide management planning (Wilson *et al*. 2014). As indicated earlier, only 13 % of countries with naturalized and invasive *Prosopis* have maps or detailed records of occurrence and scale of invasion. No information is available on the scale of *Prosopis* invasions on any of the Pacific (besides Hawaii), Indian Ocean or Caribbean Islands. Only a few African countries have a good understanding of the scale of invasions and, in Asia, information on the distribution of invasive *Prosopis* is only available for India and Pakistan. Such knowledge is essential for planning and implementing management. Bioclimatic mapping at board local scales is useful for understanding potential spread and occurrence of invasive species. However, bioclimatic models can be of limited value at very local scales as other biotic and abiotic factors come into play (Robinson *et al*. 2011). On a global scale, bioclimatic modelling is useful for highlighting which countries and species need risk assessments for purposeful introductions, and where introduction pathways need to be monitored to prevent unintentional introductions, e.g. between India and China or Iran and Turkmenistan.

Further knowledge on the ecology, local perceptions, and the ecological, economic and social benefits and impacts of *Prosopis* is needed to guide management (Wilson *et al*. 2014). Our study has highlighted that knowledge on *Prosopis* invasions is essential for management (Table 1; Fig. 6). Most of the literature comes from a handful of countries (Australia, India, Kenya, South Africa, USA), and research in other areas is needed since each region has its own set of factors that drive invasions and complicate management. There is also a need for research to better predict trends such as future densities, extent and impacts which is particularly important when it comes down to developing strategic responses. Drivers of weediness in areas where it is native such as Argentina, Mexico, Middle East and the USA require further study to improve understanding of what drives native plants to become invasive and provide insight into how to manage them.

The issue of the lack of knowledge is also present for research on the effectiveness of controlling populations using different methods. Utilization as a control method is becoming popular in many areas such as Djibouti, Ethiopia and Kenya. However, despite many reports showing how much monetary benefit *Prosopis* has provided, there is no information on how successful this approach is for controlling *Prosopis* invasions. There are also conflicting ideas on the role and success of biological control in Australia and South Africa and further work is needed (Zachariades *et al*. 2011). There is scope for identifying and potentially releasing additional biological control agents to improve control success in areas where this has been limited until now, such as in South Africa (Zachariades *et al*. 2011). Research is needed to identify novel solutions to aid the dilemma of management and contentious issues regarding invasive *Prosopis* globally. These include methods that retain the benefits, but reduce the impacts substantially.

Risk assessments need to be run for *Prosopis* species that have not been introduced yet to determine whether they might be better candidates for introduction, by providing benefits with fewer costs associated with invasiveness.

#### Dissemination of knowledge

Organizations involved in addressing land degradation and invasions should promote the dissemination of knowledge and awareness of both the impacts and benefits of *Prosopis* to prevent unwise introductions and promote management (Fig. 7). Some people still advocate the introduction of *Prosopis* species long after the severe impacts caused by invasions of these species were widely publicized; this has been described as ‘dangerous aid’ (Low 2012). Having regular multidisciplinary international meetings or workshops on *Prosopis* invasions may help to spread knowledge and create dialogue between parties, which could help to reduce contentious issues surrounding many invasive *Prosopis* species. The creation of management strategies using transdisciplinary approaches would also help to provide solutions acceptable to all stakeholders in situations where conflicting goals exist.

## Conclusions

*Prosopis* species are among the most widespread and damaging invasive woody plants in semi-arid and arid regions of the world and there is much potential for taxa to spread further. The detrimental effects on the environment and human livelihoods are escalating rapidly and there is an urgent need to devise more effective management approaches to drastically reduce adverse impacts and enhance benefits. However, there are still critical gaps in our knowledge of its ecology, impacts and how to retain benefits and reduce costs, and a lack of management capacity in many countries. Clearly focused research and strategic planning is needed to improve management, reduce costs and improve benefit flows.

## Sources of Funding

The DST-NRF Centre of Excellence for Invasion Biology and Working for Water Programme through their collaborative research project on ‘Integrated management of invasive alien species in South Africa’ – National Research Foundation (grant 85417).

## Contributions by the Authors

R.T.S. and D.M.R. conceived the idea. R.T.S., D.C.L. and N.M.P. collected the data. N.M.P. provided specialist taxonomic advice and management information. R.T.S. ran the statistics and R.T.S. and D.C.L. undertook the climate matching. R.T.S. led the writing with assistance from the others.

## Conflicts of Interest Statement

None declared.

## Supporting Information

The following Supporting Information is available in the online version of this article –

**File 1.** Methods for literature review, climate matching, regression analysis, classification and regression tree.

**File 2.** Global distribution of *Prosopis* species. Status codes (*sensu* Pyšek *et al*. 2004) are given in brackets: N, naturalized; I, invasive; NA, native; W, weedy; U, unknown. Countries partaking in management of *Prosopis* species are marked with an asterisk.

**File 3.** Climate matching output—list of climatically suitable countries and the associated species (excluding known native species).

**File 4.** Underlying information on *Prosopis* invasions worldwide.

Additional Information
