# Supplementary material for: Prosopis: a global assessment of the biogeography, benefits, impacts and management of one of the world's worst woody invasive plant taxa
Source: AoB Plants. 2014 Jun 4;6:plu027. doi: 10.1093/aobpla/plu027 (PMC4086457; doi:10.1093/aobpla/plu027)
Supplement: Additional Information [file supp_6_plu027_index.html]

Prosopis: a global assessment of the biogeography, benefits, impacts and management of one of the world's worst woody invasive plant taxa — Additional Information 

# *Prosopis*: a global assessment of the biogeography, benefits, impacts and management of one of the world's worst woody invasive plant taxa

## Additional Information

Additional Information

**Files in this Data Supplement:**

- Supplemental File S1 - doc file
- Supplemental File S2 - doc file
- Supplemental File S3 - doc file
- Supplemental File S4 - doc file
