# Supplementary material for: Prosopis: a global assessment of the biogeography, benefits, impacts and management of one of the world's worst woody invasive plant taxa
Source: AoB Plants. 2014 Jun 4;6:plu027. doi: 10.1093/aobpla/plu027 (PMC4086457; doi:10.1093/aobpla/plu027)
Supplement: Additional Information [file supp_plu027_plu027supp_file1.doc]

**File 1: Methods for literature review, climate matching, regression analysis, classification and regression tree.**

**Data collection** Literature was sourced from peer-reviewed journals, books and unpublished material (grey literature) using searches in the ISI web of Science, Scopus, JSTOR and Google Scholar. Online databases were also used, including the Invasive Species Compendium (CABI), the Global Invasive Species Database (ISSG), the Agroforestree Database, the Encyclopaedia of Life (EOL) and the Global Biodiversity Information Facility (GBIF). Multiple key words were used in the search, including “*Prosopis*”, “mesquite”, “invasive”, “alien”, “control”, “uses”, “management”, “benefits”, “impacts”, etc. as well as country names and *Prosopis* species names. Over 2 500 sources were reviewed between March and September 2013. Experts on different aspects of *Prosopis* were also consulted to verify data and to access additional information. The CIA WorldFactbook, UNEP and UNDP websites were also used to obtain social and economic data for use in the logistic regressions and classification/regression tree models. This included GDP per capita to rate economic performance and HDI a good indicator of social development that incorporates a number of variables. Similar indicators have been used by Essl *et al*. (2011) and Lotz and Allen (2013) to aid in predicting factors relating to biological invasions. Key information that was gathered included the recorded locations of *Prosopis* species: the status of the species in different regions (i.e. whether native/weedy/alien/naturalised/invasive – defined in Introduction). The literature was also reviewed to assess the presence and type of management programs implemented in each country and information on the date of introduction, uses, impacts, and scale of invasion were also collected. This data was used for the logistic regressions, classification and regression trees, to create occurrence maps and reasons of introductions, understand the global stats of introduced species as well discuss impacts, benefits and management *Prosopis*.

**Data analysis**

***Occurrence mapping*:** *Prosopis* species richness and occurrence were mapped on a country level only. Insufficient sub-national data on *Prosopis* distribution meant that more accurate assessments could only be made for very few countries and thus were not carried out. Our data therefore illustrates the presence but not the extent of *Prosopis* occurrence in any country. Maps were produced to show the countries where *Prosopis* is native, where it is weedy in native settings, where it has been introduced and become naturalised or invasive, and the potential global distribution of *Prosopis* based on climatic characteristics. To determine potential distribution, we ran bioclimatic climate matching models using Köppen-Geiger maps. *Prosopis* occurrence coordinates (point data) were downloaded from the Global Biodiversity Information Facility (www.gbif.org/‎; May 2013). Data were rigorously sorted and checked for quality (removal of duplicate records, those with coordinate errors e.g. points in the ocean) coordinates that were too coarse (only one or two decimal points), and to ensure the inclusion of only native and naturalised populations (following the protocol outlined in Donaldson *et al*. 2014). There were species in GBIF not listed in Burkart (1976) (e.g. *Prosopis nuda*, *P. ordata*, *P.* panta) were removed. This reduced the number of occurrence records for the 44 *Prosopis* species from 5000 to less than 1400.

Broad-scale climatic suitability has been shown to be an excellent predictor of potential distribution for many types of organisms (Metzger *et al*. 2013). Climatic matching was run using Köppen-Geiger maps to highlight areas suitable for *Prosopis* species around the world based on climatic characteristics (Peel *et al*. 2007). All *Prosopis* species were included. The geographic locations of the majority of the point locations occurred within Köppen zones with well-defined values and theses climate regions was included for the analysis. There was uncertainty involved with some point locations with low geographic precision which landed up in unlikely zones because they were located in areas with very steep climatic gradients where the Köppen Zone boundary location also could be inaccurate due to the pixel resolution. These points were removed from the analysis. Point coordinates from both native and invasive ranges were included to improve projections of potential invasiveness (Broennimann and Guisan, 2008). Although several species are currently much more invasive than others, no evidence exists to show a clear separation between invasive and non-invasive species based on life-history or other features of the species. We suggest that all *Prosopis* species should be regarded as potentially invasive, and that it is appropriate to define a bioclimatic envelope for the entire genus to serve as input to future risk assessments before further introductions are made.

**Statistics:** Logistic regressions were run to determine which environmental, economic and social factors were correlated whether a country here is existence of formal management initiatives for *Prosopis* or not. The analysis was run for all countries categorised as having invasive or weedy *Prosopis* populations. The predictors used were: Number of introduced *Prosopis* species, Source of introduction, Time since introduction, Distribution and extent of *Prosopis* cover known, Level of use, Level of *Prosopis* impacts, Number of publications relating to *Prosopis*, Overall knowledge of *Prosopis* invasions, GDP per capita and Human development index (HDI). This information was collected using the literature and online databases [Supporting Information – File 4]. Data from the reviewed literature for uses, impacts, understanding of scale of invasion were all put on a rating scale (low (1) to high (4)) based on evidence from the literature. Overall level of knowledge was based on a rating scale of 1 (low) to 5 (high) based on an assessment of the number of publications and the diversity of information spanning different factors relating to invasions. Therefore counties with high ratings had many publications spanning a range of topics (e.g. understanding of a range of impacts, use levels, perceptions, scale of distribution, effectiveness of management etc) whereas countries with a similar number of publications, but dealing only with impacts on plants, would score lower. For each country the literature was used to assess whether any formal management operations were being conducted by the state or NGOs. The management approach applied within each country was then categorised into the main approach utilised (chemical control, mechanical control, biological control, cultural control, control through utilisation, or integrated management – use of three or more strategies). Only countries and territories with invasive and weedy species were included. Univariate logistic regressions were run for each variable independently to assess individual contributions. A forward-stepwise logistic regression was then run using all predictor variables. This approach identified the most significant predictor variables, producing the minimal adequate model (Bucharova and van Kleunen, 2009). The logistic regression analyses were run in SPSS v 21. Data-mining techniques (Classification/regression trees) were used to identify and explore factors associated with the type of management approach that different countries are likely to adopt. Classification/regression tree models were run in Statistica v 11 using the same explanatory variables as for the logistic regressions (Table 1). These models were pruned based on variance, and the stopping parameter (minimum number of cases) was set low, at 7. This was because the overall sample size was relatively small and there were four management-approach categories.

**References**

Broennimann O, Guisan A. 2008. Predicting current and future biological invasions: both native and invaded ranges matter. Biology Letters 4:585-589.

Bucharova A, van Kleunen M. 2009. Introduction history and species characteristics partly explain naturalization success of North American woody species in Europe. Journal of Ecology 97:230-238.

Burkart A. 1976. A monograph of the genus Prosopis (Leguminosae subfam. Mimosoideae). Part 1 and 2). Catalogue of the recognised species of Prosopis. Journal of the Arnold Arboretum 57:219-249;450-526.

Donaldson JE, Hui C, Richardson DM, Robertson MP, Webber BL, Wilson JRU. 2014. Invasion trajectory of alien trees: the role of introduction pathway and planting history. *Global Change Biology* doi:10.1111/gcb.12486.

Essl F, Dullinger S, Rabitch W, Hlume PE, Hülber K, Jarošík V, Kleinbauer I, Frausmann F, Kühn I, Nentwig W, Vilá M, Genovesi P, Gherardi F, Desprez-Loustau M, Roques A, Pyšek P. 2011. Socioeconomic legacy yield an invasion debt. *PNAS* 108:203-207.

Lotz A, Allen CR. 2013. Social-ecological predictors of global invasions and extinctions. Ecology and Society 18:15 doi.org/10.5751/ES-05550-180315.

Metzger MJ, Bruce RGH, Jongman RHG, Sayre R, Trabucco A, Zomer R. 2013. A high-resolution bioclimate map of the world: a unifying framework for global biodiversity research and monitoring. Global Ecology and Biogeography 22:630-638.

Peel MC, Finlayson BL, McMahon TA. 2007. Updated world map of the Köppen-Geiger climate classification. Hydrology and Earth System Science 11:1633-1644.
