# Supplementary material for: Prosopis: a global assessment of the biogeography, benefits, impacts and management of one of the world's worst woody invasive plant taxa
Source: AoB Plants. 2014 Jun 4;6:plu027. doi: 10.1093/aobpla/plu027 (PMC4086457; doi:10.1093/aobpla/plu027)
Supplement: Additional Information [file supp_plu027_plu027supp_file2.doc]

**SUPPORTING INFORMATION**

**File 2**: Global distribution of *Prosopis* species. Status codes (*sensu* Pyšek *et al*. 2004 with additional category weedy to describe native species that are invasive in their native rages) are given in brackets: N = naturalised; I = invasive; NA = native; W = weedy; U = unknown. Countries partaking in management of Prosopis species are marked with an asterisk.

| **Country** | ***Prosopis* species status** | **References** |
| --- | --- | --- |
| Afghanistan | *P. cineraria* (NA); *P. farcta* (W) *P. juliflora* (N) | Sohrabi *et al*. 2011; GBIF, 2013 |
| Algeria | *P. farcta* (NA), *P. juliflora* (I) | Habit *et al*. 1990; Pasiecznik *et al*. 2001; ISSG, 2005 |
| Angola | *Prosopi*s spp. (N) | Poyton, 2009 |
| Antigua and Barbuda | *P. juliflora* (N) | Johnston, 1962 |
| Argentina* | *P. abbreviata* (NA), *P. affinis* (W), *P. alba* (NA), *P. algarobilla* (NA), *P. alpataco* (NA), *P. argentina* (NA) *P. caldenia* (W), *P. calingastana* (NA), *P. camperstris* (W), *P. castellanosii* (NA), *P. chilensis* (NA), *P. denudans* (NA), *P. elata* (NA), *P. ferox* (NA), *P. fiebrigii* (NA), *P. flexuosa* (NA),*P. glandulosa* (N), *P. hasslei* (W), *P. humilis* (W), *P. juliflora* (N), *P. kuntzei* (W), *P. laevigata* (NA), *P. nigra* (W), *P. pugionata* (NA), *P. reptans* (NA), *P. rojasiana* (NA), *P. ruizleali* (NA), *P. ruscifolia* (W), *P. sericantha* (NA), *P. strombulifera* (NA), *P. torquata* (NA), *P. tamarugo* (N), *P. vinalilla* (NA), *P. hybrids* (I/W) | CABI, 2005; GBIF, 2013 |
| Aruba | *P. juliflora* (N) | DCBE, 2009 |
| Ascension Island* | *P. juliflora* (I) | Pickup, 1999; Belton, 2008; |
| Australia* | *P. glandulosa* (I), *P. juliflora* (I)*, P. pallida* (I)*, P. velutina* (I)*, P.* hybrids (I) | Panetta and Carstairs, 1989 Osmond, 2003; van Klinken *et al*. 2006 |
| Azerbaijan | *P. farcta* (NA) | CABI, 2005 |
| Bahamas | *P. juliflora* (N) | CABI, 2005 |
| Bahrain | *P. farcta* (NA), *P. glandulosa* (U), *P. juliflora* (N) | Pasiecznik *et al*. 2001; AFTD, 2009 |
| Bangladesh | *P. juliflora* (N) | CABI, 2005 |
| Barbados | *P. juliflora* (N) | Jonston, 1962; |
| Belize | *P. juliflora* (NA) | AFTD, 2009 |
| Benin | *P. africana* (NA) *P. juliflora* (U) | AFTD, 2009; CABI, 2009 |
| Bermuda | *P. juliflora* (U) | CABI, 2009 |
| Bolivia | *P. alba* (NA), *P. algarobilla* (NA), *P. alpataco* (NA), *P. chilensis* (NA), *P. denudans* (NA), *P. elata* (NA), *P. ferox* (NA), *P. flexuosa* (NA), *P. juliflora* (NA), *P. kuntzei* (NA), *P. laevigata* (NA), *P. nigra* (NA), *P. pallida*(NA), *P. ruscifolia* (NA), *P. vinalillo* (NA) | Pasiecznik *et al*. 2001; Grandtner, 2005; AFTD, 2009 |
| Botswana | *P. chilensis* (I)*, P. glandulosa* (I)*, P. pallida* (N), *P. juliflora* (N), *P. velutina* (I)*, P.* hybrids (I) | Botswana Gov. 2009; Poyton, 2009; Muzila *et al*. 2011 |
| Brazil | *P. affinis*(N/W), *P. alba* (N), *P. flexuosa* (NA), *P. juliflora* (I), *P. laevigata* (NA), *P. pallida* (N), *P.rubriflora* (NA), *P. ruscifolia* (N) | Pasiecznik *et al*. 2001; Leão *et al*. 2011 de Olivera *et al*. 2012 |
| Brunei Darussalam | *P. juliflora* (U) | Pasiecznik *et al*. 2001; AFTD, 2009 |
| Burkina Faso | *P. africana* (NA), *P. juliflora* (I) | Ræbild *et al*. 2003; Weber *et al*. 2008 |
| Cambodia | *P. juliflora* (U) | AFTD, 2009 |
| Cameroon | *P. africana* (NA) | Pasiecznik et al., 2001; AFTD, 2009 |
| Cape Verde | *P. glandulosa* (N), *P. juliflora* (I), *P. pallida* (I) *P. velutina* (N), *P.* hybrids (I) | FAO, 2006; AFTD, 2009; Cienciala *et al*. 2013 |
| Cayman Islands | *P. juliflora* (N) | CABI, 2005 |
| Central African Republic | *P. africana* (NA) | AFTD, 2009 |
| Chad | *P. africana* (NA), *chilensis* (N), *P. juliflora* (I) | Pasiecznik *et al*. 2001; ISSG, 2005; Geesing *et al.* 2004 |
| Chile | *P. alba* (NA), *P. alpataco* (NA), *P. burkartii* (NA), *P. chilensis* (W),*P. flexuosa* (NA), *P. fruticosa* (NA),*P. laevigata* (NA), *P. strombulifera* (NA), *P. tamarugo* (W) | Pasiecznik *et al*. 2001; AFTD, 2009; GBIF 2013 |
| Colombia | *P. flexuosa* (NA), *P. juliflora* (W), *P. nigra* (W), *P. pallida* (NA) | Grandtner, 2005; Vallejo *et al*. 2012; GBIF, 2013 |
| Costa Rica | *P. juliflora* (NA) | Burkart, 1976 |
| Cote d’Ivore | *P. africana* (NA), *P. juliflora* (U) | CABI, 2005 |
| Cuba | *P. juliflora* (N), *P. glandulosa* (N) | Johnston, 1962; Pasiecznik *et al*. 2001 |
| Curacao | *P. juliflora* (N) | Pasiecznik *et al*. 2001 |
| Cyprus | *P. farcta* (NA) | Pasiecznik *et al*. 2004 |
| Djibouti* | *P. cineraria* (I), *P. juliflora* (I), P. pallida (I), | Pasiecznik *et al*. 2001 |
| Dominican Republic | *P. juliflora* (N) | ISSG, 2010; Pasiecznik *et al*. 2001 |
| Ecuador | *P. chilensis* (NA), *P. juliflora* (NA), *P. pallida* (NA) | Grandtner, 2005; GBIF, 2013 |
| Egypt* | *P. africana* (NA), *P. chilensis* (N), *P. farcta* (NA), *P. glandulosa* (I), *P. juliflora* (I), *P. velutina* (I) | Pasiecznik *et al*. 2001; Ghazali, 2006; Weber *et al*. 2008 |
| El Salvador | *P. juliflora* (NA) | Burkart, 1976 |
| Eritrea* | *P. juliflora* (I) | Zimmermman, 1991;  ISSG, 2005; Bokerezion, 2008 |
| Ethiopia* | *P. africana* (NA), *P. chilensis* (I), *P. juliflora* (I), *P. pallida* (I) | ISSG, 2005; Berhanu and Tesfay, 2006; Shiferaw *et al*. 2004; FARM-Africa, 2008 |
| Fiji | *P. pallida* (U) | Gallaber and Merlin, 2010 |
| Galapagos | *P. juliflora* (NA), *Prosopis spp*. (NA) | Wiggins  *et* *al*.1971 |
| Gambia | *P. africana* (NA), *P. juliflora* (I) | Pasiecznik *et al*. 2001 |
| Georgia | *P. farcta* (W) | Pasiecznik *et al*. 2001 |
| Ghana | *P. africana* (NA), *P. juliflora* (I) | Pasiecznik *et al*. 2001 |
| Guam | *P. pallida* (N) | Fosberg *et al*. 2013 |
| Guatemala | *P. juliflora* (NA) | Pasiecznik et al. 2001 |
| Guinea | *P. africana* (NA) | Pasiecznik *et al*. 2001 |
| Guinea-Bissau | *P. africana* (NA), *P. juliflora* (I) | AFTD, 2009 |
| Haiti | *P. chilensis* (U), *P. flexuosa* (IP), *P. juliflora* (I), *P. velutina* (N) | Burkart, 1976; Lee *et al*. 1992; Timyan, 1996 |
| Hawaii* | *P. juliflora* (I), *P. pallida* (I), *P. hybrids* (I) | Kaur *et al*. 2012 |
| Honduras | *P. juliflora* (NA) | Burkart, 1976 |
| India* | *P. cineraria* (W), *P. farcta* (NA), *P. glandulosa* (I), *P. juliflora* (I), *P. pallida* (N), *P. velutina* (N), *P.* hybrids (I) | Pasiecznick *et al.* 2004; CABI; 2005; FAO, 2006; AFTD, 2009; Kaur *et al*. 2012; |
| Indonesia | *P. juliflora* (U) | Pasiecznik *et al*. 2001; CABI, 2005 |
| Iran* | *P. cineraria* (W), *P. farcta* (W), *P. juliflora* (I), *P. koelziana* (NA) | Pasiecznik *et* al. 2001; AFTD, 2009; Sohribi *et al*. 2011; Sajad and Sefidi, 2012. |
| Iraq | *P. farcta* (W), *P. juliflora* (I) | Burkart,1976; Berhanu and Tesfay, 2006 |
| Israel | *P. farcta (*W), *P. glandulosa* (N), *P. juliflora* (I), *P. pallida* (N) | Pasiecznik *et al*. 2001; CABI, 2005 |
| Jamaica | *P. juliflora* (N) | Pasiecznik *et al*. 2001 |
| Jordan* | *P. farcta* (W), *P. glandulosa* (N), *P. juliflora* (I), *P. pallida* (N) | Pasiecznik *et al*. 2001; Quasem, 2007 |
| Kenya* | *P. africana* (NA), *P. chilensis* (I), *P. juliflora* (I), *P. pallida* (N) | Choge *et al*. 2002; Mwangi and Swallow, 2005; FAO, 2006; Maturi, 2013 |
| Kiribati | *P. pallida* (U) | Fosberg et al. 1976; GISD, 2013 |
| Kuwait | *P. glandulosa* (N), *P. juliflora* (I) | Burkart, 1976 |
| Laos | *P. juliflora* (N) | AFTD, 2009 |
| Lesotho | *Prosopis spp.* (N) | Poynton, 2009 |
| Liberia | *P. africana* (NA), *P. juliflora* (N) | CABI, 2005 |
| Libya | *P. farcta* (NA), *P. juliflora* (I) | CABI, 2005 |
| Madagascar | *P. juliflora* (N) | Du Pay, 1990, CABI, 2005 |
| Malaysia | *P. juliflora* (U) | CABI, 2005 |
| Malawi | *P. glandulosa* (I) | Chikuni *et al*. 2004 |
| Mali | *P. africana*(NA), *P. juliflora* (I) | Weber et al., 2008; Djoudi *et al*. 2011; |
| Mauritania | *P. chilensis*(U), *P. juliflora* (I), *P. pallida*(N) | Gritzner, 1979; Jensen and Hajej, 2001 |
| Mariana Islands | *P. pallida* (N) | Fosberg *et al*. 1976 |
| Marquesas Islands | *P. juliflora* (I), *P. pallida* (N) | Gallaber and Merlin; 2010; Lorence and Wagner, 2013 |
| Mauritius | *P. pallida* (N) | Gallaber and Merlin, 2010 |
| Mexico | *P. articulata* (NA), *P. glandulosa* (W), *P. juliflora* (NA), *P. laevigata* (NA), *P. nigra* (N), *P. palmeri* (NA), *P. pubescens* (NA), *P. reptans (NA)*, *P. tamaulipana* (NA), *P. velutina* (W), *P.* hybrids (I/W) | Grandtner, 2005 GBIF, 2013 |
| Montserrat | *P. juliflora* (N) | Gallaber and Merlin, 2010 |
| Morocco | *P. juliflora* (N) | Benata *et al*. 2008 |
| Mozambique | *P. juliflora* (I) | Witt, 2013 – pers. comm. |
| Myanmar | *P. glandulosa* (N) | Gallaber and Merlin, 2010 |
| Namibia* | *P. chilensis*(N), *P. glandulosa*(I), *P. pallida*(I), *P. juliflora* (I), P. velutina (I), *P.* hybrids (I) | Zimmerman, 1991; Smit, 2004; Poynton, 2009 |
| Netherlands Antilles | *P. juliflora* (N) | Grandtner, 2005 |
| New Caledonia | *P. pallida* (U) | MacKee, 1994 |
| Nicaragua | *P. juliflora* (NA) | AFTD, 2009 |
| Niger* | *P. africana*(NA), P. juliflora (I) | Geesing *et* al, 2004; FAO, 2006; Weber *et al*. 2008; GBIF, 2013 |
| Nigeria | *P. africana*(NA), P. juliflora (I) | Burkart, 1976; Borokini and Babalola, 2012 |
| Oman* | *P. cineraria* (NA), *P. juliflora* (I), | Ghazanfar, 1996; Al Rawahy *et al*. 2003;; Al Abri *et al*. 2004 |
| Pakistan* | *P. cineraria* (W), *P. glandulosa* (N), *P. juliflora* (I), *P. koelziana* (NA) | Pasiecznik *et al*. 2004; AFTD, 2009; Hussain *et al*. 2010; Khan *et al*. ,2011 |
| Panama | *P. juliflora* (NA) | AFTD, 2009 |
| Papua New Guinea | *P. pallida* (N), *P. juliflora* (I) | CABI, 2005; AFTD, 2009 |
| Paraguay | *P. affinis* (NA), *P. algarobilla* (NA), *P. argentina* (NA), *P. alba* (NA), *P. campestris* (W), *P. chilensis* (NA),*P. elata* (NA), *P. fiebrigii* (NA), *P. hassleri* (NA), *P. humilis* (NA), *P. kuntzei* (NA), *P. juliflora*  (NA), *P. nigra* (NA), *P. P. rojasiana* (NA), *P. ruscifolia* (W), *P. rubriflora* (NA), *P. sericantha* (NA), *P. vinalillo* (NA) | GBIF, 2013 |
| Peru | *P. alba* (NA), *P. chilensis* (NA), *P. juliflora* (NA), *P. laevigata* (NA), *P. pallida* (NA), *P. reptans* (NA) | Grandtner, 2005; GBIF, 2013 |
| Philippines | *P. juliflora* (N) | Burkart, 1976; Gallaber and Merlin, 2010 |
| Polynesia | *P. pallida* (N) | Burkart, 1976; ISSU, 2005; Lorence and Wagner, 2013 |
| Puerto Rico | *P. glandulosa* (N), *P. juliflora* (I), *P. pallida* (N) | Little and Wadsworth; 1964; GBIF, 2013 |
| Qatar | *P. cineraria* (NA), *P. glandulosa* (N), *P. juliflora* (N) | Pasiecznik *et al*. 2001 |
| Reunion | *P. glandulosa* (N), *P. juliflora* (I) | Kueffer and Lavergne, 2004; CABI, 2005 |
| Saint Helena | *P. juliflora*(N) | GISD, 2013 |
| Saudi Arabia* | *P. africana* (U), *P. cineraria* (NA), *P. farcta* (W), *P. glandulosa* (N), *P. juliflora* (I), *P. koelziana* (NA) | Pasiecznik *et al*. 2001; Hall *et al*. 2010 |
| Senegal | *P. africana* (NA), *P. alba* (N), *P. cineraria* (N), *P. pallida* (N) *P. juliflora* (I) | Diagne, 1992; Pasiecznik *et al*. 2001 |
| Sierra Leone | *P. africana* (NA), *P. chilensis* (N) | CABI, 2005, GBIF, 2013 |
| Society Islands | *P. juliflora* (U), *P. pallida* (U) | Fosberg, 1997; |
| Somalia | *P. chilensis* (I), *P. juliflora* (I) | Zollner, 1986; CABI, 2005 |
| South Africa* | *P. alba* (I), *P. chilensis* (I), *P. glandulosa* (I), *P. juliflora* (I), *P. laevigata* (I), *P. pubescens* (N), *P. velutina* (I), *P. hybrids* (I) | Zimmermann, 1991; Poynton, 2009; Van den Berg, 2010; Mazibuko, 2012 Wise *et al*. 2012 |
| Spain | *P. chilensis* (N), *P. velutina* (N) | Tilstone *et al*. 1998; Pasiecznik and Peñalvo López, in review |
| Sri Lanka | *P. cineraria* (N), *P. juliflora* (I) | Parera and Pasiecznik, 2005; AFTD, 2009 |
| St Lucia | *P. juliflora* (N) | AFTD, 2009 |
| St Vincent | *P. juliflora* (N) | AFTD, 2009 |
| Sudan* | *P. africana* (NA), *P. chilensis* (I), *P. glandulosa* (I), *P. pallida* (N), *P. juliflora* (I), *P. velutina* (I) | Burkart, 1976; El Fadl, 1997; CABI, 2005; FAO, 2006; ISSG, 2006; Bokrezion, 2008 |
| Syria | *P. farcta* (W) | ISSU, 2005 |
| Tajikistan | *P. farcta* (NA) | GBIF, 2013 |
| Tanzania* | *P. africana* (NA), *P. chilensis* (N), *P. juliflora* (I) | AFTD, 2009; Witt, 2013 – pers. comm. |
| Thailand | *P. juliflora* (U) | Pasiecznik *et al*. 2001 |
| Togo | *P. africana* (NA), *P. chilensis* (N) | AFTD, 2009 |
| Trinidad and Tobago | *P. juliflora* (N) | ISSG, 2013 |
| Tunisia | *P. chilensis* (N), *P. cineraria* (N), *P. farcta* (NA), *P. glandulosa* (N), *P. juliflora* (I), *P. laevigata* (N), *P. velutina* (N) | Habit and Saavedra, 1990 |
| Turkey | *P. farcta* (W) | Pasiecznik *et al*. 2001; ISSU, 2005 |
| Turkmenistan | *P. farcta* (NA) | GBIF, 2013 |
| Uganda | *P. africana* (NA), *P. juliflora* (N) | Pasiecznik *et al*. 2001 |
| Ukraine | *P. farcta* (W) | Pasiecznik *et al*. 2001 |
| United Arab Emirates* | *P. cineraria* (N), *P. farcta* (NA), *P. glandulosa* (N), *P. juliflora* (I) | El-Keblawy and Al-Rawi, 2007; AFTD, 2009 |
| United States* | *P. alba* (N), *P. articulata* (NA), *P. chilensis* (W), *P. cineraria* (N), *P. cinerascens* (NA), *P. farcta* (I), *P. glandulosa* (W), *P. juliflora* (I), *P. laevigata* (NA), *P. pallida* (I), *P. pubesens* (W), *P. strombulifera* (W), *P. velutina* (W), *P.* hybrids (I/W) | Johnston, 1962; Grandtner, 2005; GBIF, 2013 |
| Uruguay | *P. affinis* (NA), *P. alba* (NA), *P. caldenia* (W), *P. chilensis* (NA), *P. nigra* (NA), *P.* *ruscifolia* (NA) | GBIF, 2013 |
| Venezuela | *P. flexuosa* (NA), *P. juliflora* (W) | Grandtner, 2005; Burkart, 1976 |
| Vietnam | *P. juliflora* (N) | Pasiecznik et al. 2001 |
| Virgin Islands | *P. juliflora* (N), *P. pallida* (N) | Little and Wadsworth, 1964; Burkart, 1976 |
| Western Sahara | *P. juliflora* (N) | Habit *et al*. 1990; Witt, 2013 – pers. comm. |
| Yemen* | *P. chilensis* (I), *P. cineraria* (NA), *P. farcta* (NA), *P. glandulosa* (N), *P. juliflora* (I), *P. koelziana* (NA) | FAO, 2006; Geesing *et al*. 2004 |
| Zanzibar | *P. juliflora* (N) | Nahonyo *et al*. 2005 |
| Zimbabwe | *P. juliflora* (N), *P. pallida* (U) | White, 1962; Poynton, 2009 |

Agroforestree Database (AFTD). 2009. *Prosopis*. Kenya: World Agroforestry Centre.

Al Abri AS, Al Ajmi DS, Al Halhali AS, Al Saqry NM, Forsberg NE, Kadim IT, Mahgoub O, Richie AR. 2004. In: Salem B, Nefzaoui A, Morand-Fehr P, eds. *Nutrition and feeding strategies of sheep and goats under harsh climates*. Zaragoza: CIHEAM.

Al Rawahy SH, Al Dhafri K, Al Bahlani SS. 2003. Germination, growth and drought resistance of native and alien plant species of the genus *Prosopis* in the Sultanate of Oman. *Asian Journal of Plant Sciences* 2:1020-1023.

Belton T. 2008*. Management Strategy for Mexican thorn (Prosopis juliflora) on Ascension Island: An assessment of this species, and recommendations for management*. Bedfordshire: RSPB.

Berhanu A, Tesfaye G. 2006. The *Prosopis* dilemma, impacts on drylands biodiversity and some controlling methods. *Journal of the Drylands* 1(2):158-164.

Benata H, Mohammed O, Noureddine B, Abdelbasset B, Abdelmoumen H, Muresu R, Squartini A, El Idrissi MM. 2008. Diversity of bacteria that nodulate Prosopis juliflora in the eastern area of Morocco. *Systematic and Applied Microbiology* 31(5):378-386.

Borokini TI, Babalola FD. 2012. Management of invasive plant species in Nigeria through economic exploitation: lessons from other countries. *Management of Biological Invasions* 3(1):45-55.

Bokrezion H. 2008. *The ecological and socio-economic role of Prosopis juliflora in Eritrea: An analytical assessment within the context of rural development in the Horn of Africa.* PhD Thesis, Johannes Gutenberg University, Mainz.

Botswana Government. 2009. *Botswana fourth national report to the convention of biological diversity*. Gaborone: Botswana Government.

Burkart A. 1976. A monograph of the genus *Prosopis* (Leguminosae subfam. Mimosoideae). Part 1 and 2) Catalogue of the recognised species of *Prosopis*. *Journal of the Arnold Arboretum* 57:219-249;450-526.

Chikuni MF, Dudley CO, Sambo EY. 2004. *Prosopis glandulosa* Torry (Leguminosae-Mimosoidae) at Swang’oma, Lake Chilwa plain: A blessing in disguise. *Malawi Journal of Science and Technology* 7:10-16.

Choge SK, Ngujiri FD, Kuria MN, Busaka EA, Muthondeki JK. 2002.*The status and impact of Prosopis spp. in Kenya*. Nairobi: KEFRI.

Cienciala E, Centeio A, Balazek P, da Cruz Gomes Soares M, Russ R. 2013. Estimation of stem and tree level biomass models for *Prosopi juliflora/pallida* applicable to multi-stemmed tree species. *Trees* 27:1061-1070.

de Oliveira LSB, de Andrade LA, Fabricante JR, Gonçalves GS. 2012. Structure of a *Prosopis juliflora* (Sw.) DC. Population established in a temporary riverbed in the microregion of Cariri in the Stat of Paraiba. *Semina: Ciências Agrárias, Londrina* 33(5):1769-1778.

Diagne O. 1996. Utilization and nitrogen fixation of *Prosopis juliflora* in Senegal. In: Felker P, Moss J, eds. *Prosopis: Semiarid fuelwood and forage tree; Building consensus for the disenfranchised*. Kingsville: Centre for Semi-Arid Forest Resources.

Djoudi H, Brockhaus M, Locatelli B. 2011. Once there was a lake: vulnerability to environmental changes in northern Mali. *Regional Environmental Change* DOI 10.1007/s10113-011-0262-5.

Du Puy, 1990. *Prosopis juliflora (Sw.) DC. From Madagascar.* UK: Royal Botanic Gardens, Kew.

Dutch Caribbean Biodiversity Explorer (DCBE). 2009. *Prosopis*. Netherlands Antilles: Government of the Netherlands Antilles.

El Fadl MA. 1997. Management of *Prosopis juliflora for use in agroforestry systems in the Sudan.* Helsinki: Tropical Forestry Reports 16, University of Helsinki.

El-Keblawy A, Al-Rawai A. 2007. Impacts of the invasive exotic *Prosopis juliflora* (Sw.) D.C. on the native flora and soils of the UAE. *Plant Ecology* 190:23-35.

FAO 2006. *Problems posed by the introduction of Prosopis spp. in selected countries*. Rome: Plant Production and Protection Division, Food and Agricultural Organization of the United Nations.

FARM- Africa. 2008. *Experiences on Prosopis management case of Afar Region.* Ethiopia: FARM-Africa.

Fosberg FR, Sachet M, Oliver R. 1976. A geographical checklist of the Micronesian dicotyledonae. *Micronesia* 15:1-295.

Gallaber T, Merlin M. 2010. Biology and impacts of Pacific Island invasive species. 6. *Prosopis pallida* and *Prosopis juliflora* (Algarroba, Mesquite, Kiawe) (Fabaceae). *Pacific Science* 64: 489-526.

Geesing D, Al-Khawlani M, Abba ML. 2004. Management of introduced *Prosopis* species: Can economic exploitation control and invasive species? *Unasylva* 217:36-44.

Ghazanfar SA. 1996. Invasive *Prosopis* in the Sultanate of Oman. *Alien* 3:10.

Ghazaly UF. 2006. *Community-based management of invasive* *Prosopis juliflora in Egypt.* Washington*:* GISP.

Global Biodiversity Information Facility (GBIF). 2013. *Prosopis species Lists*. Copenhagen and Denmark: GBIF.

Global Invasive Species Database (ISSG). 2005. *Prosopis*. IUCN.

Grandtner MM. 2005. *ELSEVIER’S Directory of trees*. Amsterdam: ELSEVIER.

Gritzner JA. 1979*. Environmental degradation in Mauritania: Staff Report*. Mauritania: Board on Science and Technology for International Development, Commission on International Relations and National research Council.

Habit MA, Saavedra JC. 1990. *The current state of knowledge on* *Prosopis juliflora*. Rome, Italy: FAO.

Hall M, Llewellyn OA, Miller AG, Al-Abbasi TM, Al-Wetaid AH, Al-Harbi RJ, Al-Shammari KF. 2010. Important plant areas in the Arabian Peninsula: 2. Farasan Archipelago. *Edinburgh Journal of Botany* 67(2):189-208.

Habit MA, Saavedra JA. 1990. *The current state of knowledge on Prosopis juliflora*. II international conference on *Prosopis*. Brazil 25-29 August. Italy: FAO.

Hussain SS, Ahmed M, Siddiqui MF, Wahab M. 2010. Threatened and endangered native plants of Karachi. *International Journal of Biology and Biotechnology* 7(3):259-266.

Invasive Species Compendium (CABI). 2005. *Prosopis*. Oxfordshire, UK: CABI.

Jensen AM, Hajej MS. 2001. The road of hope: control of moving sand dunes in Mauritania. *Unasylva* 207:31-36.

Johnston MC. 1962. The North American mesquites Prosopis Sect. Algarobia (Leguminosae). *Brittonia* 14(1):72-89.

Khan I, Marwat KB, Khan IA, Ali H, Dawar K, Khan H. 2011. Invasive weeds of southern districts of Khyber Pakhtunkhwa –Pakistan. *Pakistani Journal of Weed Science Research* 17(2):161-174.

Kaur R, Gonzales WL, Llambi LD, Soriano PJ, Callaway RM, Rout ME, Gallaher JT, Inderjit. 2012. Community impacts of Prosopis juliflora invasion: Biogeographic and congeneric comparisons. PLoS One 7:e44966.

Kueffer C, Lavergne C. 2004. *Case studies on the status of invasive woody plant species in the western Indian ocean*. Rome: FAO.

Leão, TCC, de Almeida WR, de Sá Dechoum M, Ziller SR. 2011. *Espécies exόticas invasoras: no Nordeste do Brasil*. Brazil: CEPAN Instituto Hόrus.

Lee SG, Russel EJ, Bingham RL, Felker P. 1992. Discovery of thornless, non-browsed, erect tropical *Prosopis* in 3-year-old Hatian progeny trials. *Forest Ecology and Management* 48:1-13.

Little EL, Wadsworth FH. 1964. *Common trees of Porto Rico and the Virgin Islands*. Washington, D.C: Department of Agriculture, Forest Services.

Lorence DH, Wagner WL. 2013. *Flora of the Marquesas Islands*. National Tropical Botanical Garden and the Smithsonian Institution.

MacKee HS. 1994. *Catalogue des plantes introduites et cultivées en Nouvelle-Calédonie.* Paris: Muséum Natural d’Historie.

Mazibuko DM. 2012. *Phylogenetic relationships of Prosopis in South Africa: An assessment of the extent of hybridization, and the role of genome size and seed size in the invasion dynamics.* MSc Thesis, Stellenbosch University, Stellenbosch.

Muturi GM, Poorter L, Mohren GMJ, Kigomo BN. 2013. Ecological impacts of *Prosopis* species invasion in Turkwel riverine forest, Kenya. *Journal of Arid Environments* 92:89-97.

Muzila M, Setshogo MP, Moseki B, Morapedi R. 2011. An assessment of *Prosopis* L. in the Bokspits area, south-western Botswana, based on morphology. *The African Journal of Plant Science and Biotechnology* 5:75-80.

Mwangi M, Swallow B. 2005. *Invasion of Prosopis juliflora and local livelihoods: Case study from the Lake Baringo area of Kenya. ICRAF Working Paper – no. 3.* Nairobi: World Agroforestry Centre.

Nahonyo CL, Mwasumbi LB, Msuya CA, Masao CA, Suya TB, Shing’weda C. 2005. NGEZI – *Vumawimbi Forest Reserves biodiversity inventory report*. Dar es Salam: Department of Zoology and Marin Biology, University of Dar es Salam.

Osmond R. 2003. *Best practice manual, mesquite: Control and management options for mesquite (Prosopis spp.) in Australia*. Queensland: National Weeds Programme and Queensland Department of Natural Resources and Mines.

Panetta FD, Carstairs SA. 1989. Isozymic discrimination of tropical Australian populations of mesquite (*Prosopis* spp.): implications for biological control. *Weed Research* 29:157-165.

Pasiecznik NM, Felker P, Harris PJC, Harsh LN, Cruz G, Tewari JC, Cadoret K, Maldonado LJ. 2001. *The Prosopis juliflora-Prosopis pallida complex: A monograph*. Coventry, UK: HDRA.

Pasiecznik NM, Harris PJC, Smith SJ. 2004. *Identifying tropical Prosopis species: A field guide*. Coventry, UK: HDRA.

Pasiecznik NM, Peñalvo López E. In review. 25 year results from an arid zone tree species elimination trial in Almeria, Spain, and an invasive risk assessment of the exotic species introduced. *Biodiversity and Conservation*

Perera ANF, Pasiecznik NM. 2005. *Using invasive Prosopis to improve livelihoods in Sri Lanka*. Coventry UK: HDRA.

Pickup AR. 1999. *Ascension Island Management Plan*. Report from RSPB and Birdlife International.

Poynton RJ. 2009. *Tree planting in southern Africa, volume 3: Other genera*. Pretoria, South Africa: Department of Agriculture, Forestry and Fisheries.

Qasem JR. 2007. Chemical control of *Prosopis farcta* (Banks and Sol.) Macbride in the Jordan Valley. *Crop Protection* 26:572-575.

Ræbild A, Diallo BO, Graudal L, Dao L, Sanou J. 2003.*Evaluation of a species and provenance trial of Prosopis at Gonsé, Nurkina Faso*. Rome: FAO.

Sajad G, Sefidi K. 2012. Comparison of sustainable forest management (SFM) trends at global and country levels: case study in Iran. *Journal of forestry Research* 23(2):311-317.

Shiferaw H, Teketay D, Nemomissa S, Assefa F. 2004.Some biological characteristics that foster the invasion of *Prosopis juliflora* (Sw.) DC.at Middle Awash Rift Valley Area, north-eastern Ethiopia. *Journal of Arid Environments* 58:135-154.

Smit P. 2004. Prosopis: a review of existing knowledge relevant to Namibia. *Journal of the Scientific Society* 52:13-40.

Sohrabi S, Gherekhloo J, Mohassel MHR, Ghanbari A, Mahalati MN. 2011. *Cardinal temperatures of three invasive weeds in Iran*. Acuna, Switzerland: 3^rd^ International Symposium on Weeds and Invasive Plants.

Tilstone GH, Pasiecznik NM, Harris PJC, Wainwright SJ. 1998. The growth of multipurpose tree species in the Almeria province of Spain and its relationship to native plant communities. *International Tree Crops Journal* 9(4):247-259.

Timyan J. 1996. BWA YO: *Important trees of Hati*. Washington, D.C. South-East Consortium for International Development.

Van den Berg EC. 2010. *Detection, quantification and monitoring Prosopis spp. in the Northern Cape Province of South Africa using Remote Sensing and GIS*. MSc Thesis, North-West University, Potchefstroom.

van Klinken RD, Graham J, Flack LK. 2006. Population ecology of hybrid mesquite (*Prosopis* species) in Western Australia: how does it differ from native range invasions and what are the implications for impacts and management? *Biological Invasions* 8:727-741.

Vallejo VE, Arbeli Z, Terán W, Lorenz N, Dick RP, Roldna F. 2012. Effect of land management and *Prosopis juliflora* (Sw.) DC trees on soil microbial community and enzyme activities in intensive silvopastoral systems of Colombia. *Agriculture, Ecosystems and Environment* 150:139-148.

Weber JC, Larwanou M, Abasse TA, Kalinganire A. 2008. Growth and survival of *Prosopis africana* provenances tested in Niger and related rainfall gradients in the West African Sahel. *Forest Ecology and Management* 256:585-592.

White F. 1962. *Forest and Flora of Northern Rhodesia*. Oxford, UK: Oxford University Press.

Wiggens IR, Proter M, Anderson EF. 1971. *Flora of the Galápagos Islands*. Stanford: Stanford University Press.

Wise RM, van Wilgen BW, Le Maitre DC. 2012. Costs, benefits and management options for an invasive alien tree species: The case of mesquite in the Northern Cape, South Africa. *Journal of Arid Environments* 84:80-90.

Zimmermann HG. 1991. Biological control of Prosopis, *Prosopis* spp. (Fabaceae), in South Africa. *Agriculture, Ecosystems and Environment* 37:175-186.

Zollner D. 1986. Sand dune stabilization in central Somalia. *Forest Ecology and Management* 16:223-232.
