# Supplementary material for: Prosopis: a global assessment of the biogeography, benefits, impacts and management of one of the world's worst woody invasive plant taxa
Source: AoB Plants. 2014 Jun 4;6:plu027. doi: 10.1093/aobpla/plu027 (PMC4086457; doi:10.1093/aobpla/plu027)
Supplement: Additional Information [file supp_plu027_plu027supp_file3.doc]

**SUPPORTING INFORMATION**

**File 2. Bioclimatic modelling output – list of climatically suitable countries and the associated species (excluding known native species)**

| **Country** | ***Prosopis* species** |
| --- | --- |
| Afghanistan | *P. abbreviate; P. affinis; P. africana; P. alba; P. algarobilla; P. alpataco; P. argentina; P. articulata; P. caldenia; P. castellanosii; P. chilensis; P. cinerascens; P. denudans; P. elata; P. ferox; P. fiebrigii; P. flexousa; P. glandulosa; P. hassleri; P. humilis; P. juliflora; P. kuntzei; P. laevigata; P. nigra; P. pallida; P. palmeri; P. pubescens; P. reptans; P. rojasiana; P. ruscifolia; P. sericantha; P. strombulifera; P. tamarugo; P. torquata; P. velutina; P. vinalillo* |
| Albania | *P alpataco; P. affinis; P. alba; P. algarobilla; P. alpataco; P. caldenia; P. cinerascens; P. denudans; P. elata; P. farcta; P. fiebrigii; P. flexousa; P. glandulosa; P. hassleri; P. humilis; P. juliflora; P. laevigata; P. nigra; P. pallida; P. pubescens; P. reptans; P. ruscifolia; P. sericantha; P. strombulifera; P. velutina* |
| Algeria | *P. abbreviata; P. affinis; P. africana; P. alba; P. alpataco; P. argentina; P. articulata; P. caldenia; P. castellanosii; P. chilensis; P. cineraria; P. cinerascens; P. denudans; P. elata; P. ferox; P. flexousa; P. glandulosa; P. hassleri; P. juliflora; P. kuntzei ; P. laevigata; P. nigra; P. pallida; P. palmeri; P. pubescens; P. reptans; P. rojasiana ; P. ruscifolia; P. sericantha; P. strombulifera; P. tamarugo; P. torquata; P. velutina; P. vinalillo* |
| Angola | *P. abbreviata; P. affinis; P. africana; P. alba; P. alpataco; P. argentina; P. articulata; P. caldenia; P. campestris; P. castellanosii; P. chilensis; P. cineraria; P. denudans; P. elata; P. farcta; P. ferox; P. fiebrigii; P. flexousa; P. glandulosa; P. hassleri; P. juliflora; P. kuntzei; P. laevigata; P. nigra; P. pallida; P. palmeri; P. pubescens; P. reptans; P. ruscifolia; P. sericantha; P. strombulifera; P. tamarugo; P. torquata; P. velutina; P. vinalillo* |
| Argentina | *P. africana; P. articulata; P. cineraria; P. cinerascens; P. farcta; P. glandulosa; P. pallida; P. pubescens; P. tamarugo; P. velutina* |
| Armenia | *P. alpataco; P. abbreviata; P. affinis; P. africana; P. alba; P. algarobilla; P. alpataco; P. articulata; P. caldenia; P. cineraria; P. cinerascens; P. elata; P. fiebrigii; P. glandulosa; P. hassleri; P. humilis; P. juliflora; P. kuntzei ; P. laevigata; P. nigra; P. pallida; P. palmeri; P. pubescens; P. reptans; P. ruscifolia; P. sericantha; P. strombulifera; P. torquata; P. velutina; P.flexousa* |
| Australia | *P alpataco; P. abbreviata; P. affinis; P. africana; P. alba; P. algarobilla; P. alpataco; P. articulata; P. caldenia; P. campestris; P. castellanosii; P. chilensis; P. cineraria; P. cinerascens; P. denudans; P. elata; P. farcta; P. ferox; P. fiebrigii; P. glandulosa; P. hassleri; P. humilis; P. juliflora; P. kuntzei ; P. laevigata; P. nigra; P. pallida; P. palmeri; P. pubescens; P. rojasiana; P. ruscifolia; P. sericantha; P. strombulifera; P. torquata; P. velutina; P. vinalillo; P.flexousa; P.reptans* |
| Azerbaijan | *P. abbreviata; P. affinis; P. africana; P. alba; P. algarobilla; P. alpataco; P. argentina; P. articulata; P. caldenia; P. cineraria; P. cinerascens; P. elata; P. farcta; P. fiebrigii; P. glandulosa; P. hassleri; P. humilis; P. juliflora; P. kuntzei; P. laevigata; P. nigra; P. pallida; P. palmeri; P. pubescens; P. reptans; P. ruscifolia; P. sericantha; P. strombulifera; P. torquata; P. velutina; P. flexousa* |
| Bahamas | *P. abbreviata; P. affinis; P. africana; P. alba; P. alpataco; P. argentina; P. caldenia; P. castellanosii; P. chilensis; P. cineraria;*  *P. denudans; P. elata; P. farcta; P. ferox; P. fiebrigii; P. flexousa;*  *P. glandulosa; P. hassleri; P. juliflora; P. kuntzei; P. laevigata;*  *P. nigra; P. pallida; P. pubescens; P. ruscifolia; P. sericantha; P. strombulifera; P. tamarugo; P. torquata; P. velutina; P. vinalillo* |
| Bahrain | *P. abbreviata; P. africana; P. alba; P. alpataco; P. argentina; P. articulata; P. cineraria; P. farcta; P. glandulosa; P. juliflora; P. laevigata; P. pallida; P. palmeri; P. pubescens; P. reptans; P. strombulifera; P. torquata; P. velutina* |
| Bangladesh | *P. affinis; P. africana; P. alba; P. caldenia; P. campestris; P. chilensis; P. cineraria; P. elata; P. fiebrigii; P. flexousa; P. glandulosa; P. hassleri; P. juliflora; P. kuntzei; P. laevigata; P. nigra; P. pallida; P. palmeri; P. reptans; P. ruscifolia; P. sericantha; P. torquata; P. vinalillo* |
| Barbados | *P. affinis; P. africana; P. alba; P. chilensis; P. cineraria; P. elata; P. fiebrigii; P. glandulosa; P. hassleri; P. juliflora; P. kuntzei; P. laevigata;P. nigra; P. pallida; P. ruscifolia; P. sericantha; P. vinalillo* |
| Belize | *P. affinis; P. chilensis; P. kuntzei ; P. pallida* |
| Benin | *P. abbreviata; P. affinis; P. africana; P. alba; P. alpataco; P. argentina; P. caldenia; P. castellanosii; P. chilensis; P. cineraria; P. denudans; P. elata; P. farcta; P. ferox; P. fiebrigii; P. flexousa; P. glandulosa; P. hassleri; P. juliflora; P. kuntzei; P. laevigata; P. nigra; P. pallida; P. pubescens; P. ruscifolia; P. sericantha; P. strombulifera; P. torquata; P. velutina; P. vinalillo* |
| Bhutan | *P. alba; P. alpataco; P. articulata; P. caldenia; P. campestris; P. chilensis; P. flexousa; P. glandulosa; P. hassleri; P. juliflora; P. kuntzei; P. laevigata; P. nigra; P. pallida; P. palmeri; P. reptans; P. ruscifolia; P. sericantha; P. torquata* |
| Bolivia | *P. africana; P. articulata; P. cineraria; P. cinerascens; P. denudans; P. farcta; P. fiebrigii; P. glandulosa; P. hassleri; P. humilis; P. palmeri; P. pubescens; P. reptans; P. rojasiana; P. sericantha; P. strombulifera; P. tamarugo; P. velutina* |
| Bosnia and Herzegovina | *P. affinis; P. alba; P. algarobilla; P. alpataco; P. caldenia; P. cinerascens; P. elata; P. fiebrigii; P. flexousa; P. glandulosa; P. hassleri; P. humilis; P. juliflora; P. kuntzei; P. laevigata; P. nigra; P. pallida; P. pubescens; P. reptans; P. ruscifolia; P. sericantha; P. strombulifera; P. velutina* |
| Botswana | *P. abbreviata; P. africana; P. alba; P. alpataco; P. articulata; P. caldenia; P. castellanosii; P. chilensis; P. cineraria; P. denudans; P. farcta; P. ferox; P. flexousa; P. glandulosa; P. juliflora; P. laevigata; P. nigra; P. pallida; P. palmeri; P. pubescens; P. reptans; P. sericantha; P. strombulifera; P. tamarugo; P. torquata; P. velutina* |
| Brazil | *P. abbreviata; P. africana; P. algarobilla; P. alpataco; P. alpataco; P. argentina; P. articulata; P. caldenia; P. campestris; P. castellanosii; P. chilensis; P. cineraria; P. cinerascens; P. denudans; P. elata; P. farcta; P. ferox; P. fiebrigii; P. glandulosa; P. hassleri; P. humilis; P. juliflora; P. kuntzei; P. nigra;*  *P. pallida; P. palmeri; P. pubescens; P. reptans; P. ruscifolia; P. sericantha; P. strombulifera; P. tamarugo; P. torquata; P. velutina; P. vinalillo* |
| Brunei | *P. juliflora; P. kuntzei* |
| Bulgaria | *P. affinis; P. alba; P. algarobilla; P. alpataco; P. caldenia; P. chilensis; P. cinerascens; P. denudans; P. elata; P. farcta; P. fiebrigii; P. flexousa; P. glandulosa; P. hassleri; P. humilis; P. juliflora; P. kuntzei; P. laevigata; P. nigra; P. pallida; P. pubescens; P. reptans; P. ruscifolia; P. sericantha; P. strombulifera; P. velutina* |
| Burkina Faso | *P. abbreviata; P. affinis; P. alba; P. alpataco; P. argentina; P. articulata; P. caldenia; P. castellanosii; P. chilensis; P. cineraria; P. denudans; P. elata; P. farcta; P. ferox; P. fiebrigii; P. flexousa; P. glandulosa; P. hassleri; P. juliflora; P. kuntzei; P. laevigata; P. nigra; P. pallida; P. palmeri; P. pubescens; P. reptans; P. ruscifolia; P. sericantha; P. sericantha; P. strombulifera; P. tamarugo; P. torquata; P. velutina; P. vinalillo* |
| Burma | *P. abbreviata; P. affinis; P. africana; P. alba; P. alpataco; P. argentina; P. articulata; P. caldenia; P. campestris; P. castellanosii; P. chilensis; P. cineraria; P. denudans; P. elata; P. farcta; P. ferox; P. fiebrigii; P. flexousa; P. glandulosa; P. hassleri; P. juliflora; P. kuntzei; P. laevigata; P. nigra; P. pallida; P. palmeri; P. pubescens; P. reptans; P. ruscifolia; P. sericantha; P. strombulifera; P. tamarugo; P. torquata; P. velutina; P. vinalillo* |
| Burundi | *P. affinis; P. africana; P. alba; P. alpataco; P. articulata; P. chilensis; P. cineraria; P. denudans; P. elata; P. fiebrigii; P. flexousa; P. glandulosa; P. hassleri; P. juliflora; P. kuntzei; P. nigra; P. pallida; P. ruscifolia; P. sericantha; P. strombulifera; P. velutina; P. vinalillo* |
| Cambodia | *P. affinis; P. africana; P. alba; P. chilensis; P. cineraria; P. elata; P. fiebrigii; P. glandulosa; P. hassleri; P. juliflora; P. kuntzei; P. laevigata; P. nigra; P. pallida; P. ruscifolia; P. sericantha; P. vinalillo* |
| Cameroon | *P. abbreviata; P. affinis; P. alba; P. alpataco; P. argentina; P. articulata; P. caldenia; P. castellanosii; P. chilensis; P. cineraria; P. denudans; P. elata; P. farcta; P. ferox; P. fiebrigii; P. flexousa; P. glandulosa; P. hassleri; P. juliflora; P. kuntzei; P. laevigata; P. nigra; P. pallida; P. palmeri; P. pubescens; P. reptans; P. ruscifolia; P. sericantha; P. strombulifera; P. tamarugo; P. torquata; P. velutina; P. vinalillo* |
| Canada | *P. abbreviata; P. africana; P. alba; P. argentina; P. articulata; P. chilensis; P. cineraria; P. denudans; P. flexousa; P. laevigata; P. pallida; P. palmeri; P. pubescens; P. reptans; P. strombulifera; P. torquata; P. velutina* |
| Cape Verde | *P. abbreviata; P. africana; P. alba; P. alpataco; P. argentina P. articulata; P. caldenia; P. castellanosii; P. chilensis; P. cineraria; P. denudans; P. farcta; P. ferox; P. flexousa; P. glandulosa; P. juliflora; P. laevigata; P. nigra; P. pallida; P. palmeri; P. pubescens; P. reptans; P. sericantha; P. strombulifera; P. tamarugo; P. torquata; P. velutina* |
| Central African Republic | *P. abbreviata; P. affinis; P. alba; P. alpataco; P. argentina; P. caldenia; P. castellanosii; P. chilensis; P. cineraria; P. denudans; P. elata; P. farcta; P. ferox; P. fiebrigii; P. flexousa; P. glandulosa; P. hassleri; P. juliflora; P. kuntzei; P. laevigata; P. nigra; P. pallida; P. pubescens; P. ruscifolia; P. sericantha; P. sericantha; P. strombulifera; P. tamarugo; P. torquata; P. velutina; P. vinalillo* |
| Chad | *P. abbreviata; P. affinis; P. alba; P. alpataco; P. argentina; P. articulata; P. caldenia; P. caldenia; P. castellanosii; P. chilensis; P. cineraria; P. cinerascens; P. denudans; P. elata; P. farcta; P. ferox; P. fiebrigii; P. flexousa; P. glandulosa; P. hassleri; P. juliflora; P. kuntzei; P. laevigata; P. nigra; P. pallida; P. palmeri; P. pubescens; P. reptans; P. rojasiana; P. ruscifolia; P. sericantha; P. strombulifera; P. tamarugo; P. torcuata; P. torquata; P. velutina; P. vinalillo* |
| Chile | *P. abbreviata; P. affinis; P. africana; P. argentina; P. articulata; P. caldenia; P. campestris; P. cineraria; P. cinerascens; P. denudans; P. elata; P. farcta; P. ferox’ P. glandulosa; P. hassleri; P. juliflora; P. kuntzei; P. nigra;*  *P. pallida; P. palmeri; P. pubescens; P. reptans; P. rojasiana;*  *P. ruscifolia; P. sericantha; P. torcuata; P. velutina; P. vinalillo* |
| China | *P. abbreviata; P. affinis; P. africana; P. alba; P. algarobilla; P. alpataco; P. argentina; P. articulata; P. caldenia; P. campestris; P. chilensis; P. cineraria; P. cinerascens; P. elata; P. farcta; P. ferox; P. fiebrigii; P. flexousa; P. glandulosa; P. hassleri; P. humilis; P. juliflora; P. kuntzei; P. laevigata; P. nigra; P. pallida; P. palmeri; P. pubescens ;P. reptans; P. rojasiana; P. ruscifolia; P. sericantha; P. strombulifera; P. torquata; P. velutina; P. vinalillo* |
| Colombia | *P. abbreviata; P. affinis; P. africana; P. alba; P. alpataco; P. argentina; P. articulata; P. caldenia; P. castellanosii; P. chilensis; P. cineraria; P. denudans; P. elata; P. farcta; P. ferox; P. fiebrigii; P. glandulosa; P. hassleri; P. kuntzei; P. laevigata; P. palmeri; P. pubescens; P. reptans; P. ruscifolia; P. sericantha; P. strombulifera; P. tamarugo; P. torquata; P. velutina; P. vinalillo* |
| Comoros | *P. juliflora; P. kuntzei* |
| Congo | *P. affinis; P. africana; P. alba; P. chilensis; P. cineraria; P. elata; P. fiebrigii; P. glandulosa; P. hassleri; P. juliflora; P. kuntzei; P. laevigata; P. nigra; P. pallida; P. ruscifolia; P. sericantha; P. vinalillo* |
| Congo (Democratic Republic of the) | *P. affinis; P. africana; P. alba ;P. alpataco; P. articulata; P. caldenia; P. campestris; P. chilensis; P. cineraria; P. elata; P. fiebrigii; P. flexousa; P. glandulosa; P. hassleri; P. juliflora; P. kuntzei; P. laevigata; P. nigra; P. pallida; P. palmeri; P. reptans; P. ruscifolia; P. sericantha; P. torquata; P. vinalillo* |
| Costa Rica | *P. affinis; P. africana; P. alba; P. alpataco; P. articulata; P. chilensis; P. cineraria; P. elata; P. fiebrigii; P. glandulosa; P. hassleri; P. kuntzei; P. laevigata; P. nigra; P. pallida; P. ruscifolia; P. sericantha; P. vinalillo* |
| Cote d'Ivoire | *P. affinis; P. alba; P. chilensis; P. cineraria; P. elata; P. fiebrigii; P. glandulosa; P. hassleri; P. juliflora; P. kuntzei; P. laevigata; P. nigra; P. pallida; P. ruscifolia; P. sericantha; P. vinalillo* |
| Croatia | *P. affinis; P. alba; P. algarobilla; P. alpataco; P. caldenia; P. cinerascens; P. elata; P. fiebrigii; P. flexousa; P. glandulosa; P. hassleri; P. humilis; P. juliflora; P. kuntzei; P. laevigata; P. nigra; P. pallida; P. pubescens; P. reptans; P. ruscifolia; P. sericantha; P. strombulifera; P. velutina* |
| Cuba | *P. affinis; P. africana; P. alba; P. chilensis; P. cineraria; P. elata; P. fiebrigii; P. glandulosa; P. hassleri; P. juliflora; P. kuntzei; P. laevigata; P. nigra; P. pallida; P. ruscifolia; P. sericantha; P. vinalillo* |
| Cyprus | *P. abbreviata; P. affinis; P. alba; P. algarobilla; P. alpataco; P. argentina; P. caldenia; P. castellanosii; P. chilensis; P. cinerascens; P. denudans; P. elata; P. ferox; P. fiebrigii; P. flexousa; P. glandulosa; P. hassleri; P. humilis; P. juliflora; P. kuntzei; P. laevigata; P. nigra; P. pallida; P. pubescens; P. reptans; P. ruscifolia; P. sericantha; P. strombulifera; P. tamarugo; P. torquata; P. velutina* |
| Djibouti | *P. abbreviata; P. africana; P. alba; P. alpataco; P. argentina; P. articulata; P. cineraria; P. farcta; P. glandulosa; P. juliflora; P. laevigata; P. pallida; P. palmeri; P. pubescens; P.reptans; P. strombulifera; P. torquata; P. velutina* |
| Dominica | *P. juliflora; P. kuntzei* |
| Dominican Republic | *P. abbreviata; P. affinis;P. africana; P. alba; P. argentina; P. caldenia; P. castellanosii; P. chilensis; P. cineraria; P. elata; P. farcta; P. ferox; P. fiebrigii; P. flexousa; P. glandulosa; P. hassleri; P. juliflora; P. kuntzei; P. laevigata; P. nigra; P. pallida; P. pubescens; P. ruscifolia; P. sericantha; P. strombulifera; P. tamarugo; P. torquata; P. velutina; P. vinalillo* |
| Ecuador | *P. abbreviata; P. affinis; P. africana; P. alba; P. alpataco; P. argentina; P. articulata; P. caldenia; P. castellanosii; P. cineraria; P. denudans; P. elata; P. farcta; P. ferox; P. fiebrigii; P. flexousa; P. glandulosa; P. hassleri; P. kuntzei;*  *P. laevigata; P. nigra; P. palmeri; P. pubescens; P. reptans; P. ruscifolia; P. sericantha; P. strombulifera; P. tamarugo; P. torquata; P. velutina; P. vinalillo* |
| Egypt | *P. affinis; P. alba; P. argentina; P. articulata; P. caldenia; P. chilensis; P. cineraria; P. cinerascens; P. elata; P. ferox; P. flexousa; P. glandulosa; P. hassleri; P. juliflora; P. kuntzei; P. laevigata; P. nigra; P. pallida; P. palmeri; P. pubescens; P. reptans; P. rojasiana; P. ruscifolia; P. sericantha; P. strombulifera; P. torcuata; P. velutina; P. vinalillo* |
| El Salvador | *P. affinis; P. africana; P. alba; P. chilensis; P. cineraria; P. elata; P. fiebrigii; P. glandulosa; P. hassleri; P. kuntzei; P. laevigata; P. nigra; P. pallida; P. ruscifolia; P. sericantha; P. vinalillo* |
| Equatorial Guinea | *P. affinis; P. africana; P. alba; P. chilensis; P. cineraria; P. elata; P. fiebrigii; P. glandulosa; P. hassleri; P. juliflora; P. kuntzei; P. laevigata; P. nigra; P. pallida; P. ruscifolia; P. sericantha;*  *P. vinalillo* |
| Eritrea | *P. abbreviata; P. affinis; P. africana; P. alba; P. algarobilla; P. alpataco; P. argentina; P. articulata; P. caldenia; P. chilensis; P. cineraria; P. cinerascens; P. denudans; P. elata; P. farcta;P. fiebrigii; P. flexousa;P. glandulosa;P. hassleri; P. humilis; P. juliflora; P. kuntzei; P. laevigata; P. nigra; P. pallida; P. palmeri; P. pubescens; P. reptans; P. ruscifolia; P. sericantha; P. strombulifera; P. torquata; P. velutina; P. vinalillo* |
| Ethiopia | *P. abbreviata; P. affinis; P. alba; P. alpataco; P. argentina; P. articulata; P. caldenia; P. campestris; P. chilensis; P. cineraria; P. denudans; P. elata; P. farcta; P. fiebrigii; P. flexousa; P. glandulosa; P. hassleri; P. juliflora; P. kuntzei; P. laevigata; P. nigra; P. pallida; P. palmeri; P. pubescens; P. reptans; P. ruscifolia; P. sericantha; P. strombulifera; P. torquata; P. velutina; P. vinalillo* |
| Fiji | *P. juliflora; P. kuntzei* |
| France | *P. affinis; P. alba; P. algarobilla; P. caldenia; P. chilensis; P. cinerascens; P. denudans; P. elata; P. farcta; P. fiebrigii; P. flexousa; P. glandulosa; P. hassleri; P. humilis; P. juliflora; P. laevigata; P. nigra; P. pallida; P. pubescens; P. reptans; P. ruscifolia; P. sericantha; P. trombulifera; P. velutina* |
| French Guiana | *P. affinis; P. chilensis; P. juliflora; P. kuntzei; P. pallida* |
| Gabon | *P. affinis; P. africana; P. alba; P. chilensis; P. cineraria; P. elata; P. fiebrigii; P. glandulosa; P. hassleri; P. juliflora; P. kuntzei; P. laevigata; P. nigra; P. pallida; P. ruscifolia; P. sericantha; P. vinalillo* |
| Gambia | *P. abbreviata; P. affinis; P. africana; P. alba; P. alpataco; P. argentina; P. caldenia; P. castellanosii; P. chilensis; P. cineraria;*  *P. denudans; P. elata; P. farcta; P. ferox; P. fiebrigii; P. flexousa; P. glandulosa; P. hassleri; P. juliflora; P. kuntzei; P. laevigata; P. nigra; P. pallida; P. pubescens; P. ruscifolia; P. sericantha; P. strombulifera; P. tamarugo; P. torquata; P. velutina; P. vinalillo* |
| Gaza Strip | *P. abbreviata; P. alba; P. alpataco; P. argentina; P. caldenia; P. castellanosii; P. chilensis; P. denudans; P. farcta; P. ferox; P. flexousa; P. glandulosa; P. juliflora; P. laevigata; P. nigra; P. pubescens; P. sericantha; P. strombulifera; P. tamarugo; P. torquata; P. velutina* |
| Georgia | *P. affinis; P. alba; P. algarobilla; P. alpataco; P. caldenia; P. cinerascens; P. elata; P. fiebrigii; P. flexousa; P. glandulosa; P. hassleri; P. humilis; P. kuntzei; P. laevigata; P. nigra; P. pallida; P. pubescens; P. reptans; P. ruscifolia; P. sericantha; P. strombulifera; P. velutina* |
| Ghana | *P. affinis P. alba; P. chilensis; P. cineraria; P. elata; P. fiebrigii; P. glandulosa; P. hassleri; P. juliflora; P. kuntzei; P. laevigata; P. nigra; P. pallida; P. ruscifolia; P. sericantha; P. vinalillo* |
| Greece | *P. abbreviata; P. affinis; P. africana; P. alba; P. algarobilla; P. alpataco; P. argentina; P. articulata; P. caldenia; P. chilensis; P. cineraria; P. cinerascens; P. denudans; P. elata; P. farcta; P. fiebrigii; P. flexousa; P. glandulosa; P. hassleri; P. humilis; P. juliflora; P. kuntzei; P. laevigata; P. nigra; P. pallida; P. palmeri; P. pubescens; P. reptans; P. ruscifolia; P. sericantha; P. strombulifera; P. torquata; P. velutina* |
| Guadeloupe | *P. affinis; P. chilensis; P. juliflora; P. kuntzei; P. pallida* |
| Guatemala | *P. affinis; P. africana; P. alba; P. articulata; P. chilensis; P. cineraria; P. elata; P. fiebrigii; P. glandulosa; P. hassleri; P. kuntzei; P. laevigata; P. nigra; P. pallida; P. ruscifolia; P. sericantha; P. vinalillo* |
| Guinea | *P. affinis; P. alba; P. chilensis; P. cineraria; P. elata; P. fiebrigii; P. glandulosa; P. hassleri; P. juliflora; P. kuntzei; P. laevigata; P. nigra; P. pallida; P. ruscifolia; P. sericantha; P. vinalillo* |
| Guinea-Bissau | *P. affinis; P. alba; P. chilensis; P. cineraria; P. elata; P. fiebrigii; P. glandulosa; P. hassleri; P. juliflora; P. kuntzei; P. laevigata; P. nigra; P. pallida; P. ruscifolia; P. sericantha; P. vinalillo* |
| Guyana | *P. affinis; P. africana; P. alba; P. chilensis; P. cineraria; P. elata; P. fiebrigii; P. glandulosa; P. hassleri; P. juliflora; P. kuntzei; P. laevigata; P. nigra; P. pallida; P. ruscifolia; P. sericantha; P. vinalillo* |
| Haiti | *P. abbreviata; P. affinis; P. africana; P. alba; P. alpataco; P. argentina; P. articulata; P. caldenia; P. castellanosii; P. chilensis; P. cineraria; P. elata; P. farcta; P. ferox; P. fiebrigii; P. flexousa; P. glandulosa; P. hassleri; P. juliflora; P. kuntzei; P. laevigata; P. nigra; P. pallida; P. pubescens; P. ruscifolia; P. sericantha; P. strombulifera; P. tamarugo; P. torquata; P. velutina; P. vinalillo* |
| Honduras | *P. affinis; P. africana; P. alba; P. algarobilla; P. alpataco; P. alpataco; P. articulata; P. caldenia; P. campestris; P. chilensis; P. cineraria; P. cinerascens; P. elata; P. fiebrigii; P. flexousa; P. glandulosa; P. hassleri; P. humilis; P. kuntzei; P. laevigata; P. nigra; P. pallida; P. palmeri; P. pubescens; P. reptans; P. ruscifolia; P. sericantha; P. strombulifera; P. torquata; P. velutina; P. vinalillo* |
| India | *P. abbreviata; P. affinis; P. africana; P. alba; P. algarobilla; P. alpataco; P. argentina; P. articulata; P. caldenia; P. campestris; P. castellanosii; P. chilensis; P. cinerascens; P. denudans; P. elata; P. ferox; P. fiebrigii; P. flexousa; P. glandulosa; P. hassleri; P. humilis; P. juliflora; P. kuntzei; P. laevigata; P. nigra; P. pallida; P. palmeri; P. pubescens; P. reptans; P. reptans; P. rojasiana; P. ruscifolia; P. sericantha; P. strombulifera; P. tamarugo; P. torquata; P. velutina; P. vinalillo* |
| Indonesia | *P. affinis; P. africana; P. alba; P. articulata; P. chilensis; P. chilensis; P. cinerariaP. elata; P. fiebrigii; P. glandulosa; P. hassleri; P. juliflora; P. laevigata; P. nigra; P. pallida; P. ruscifolia; P. sericantha; P. vinalillo* |
| Iran | *P. abbreviata; P. affinis; P. africana; P. alba; P. algarobilla; P. alpataco; P. argentina; P. articulata; P. caldenia; P. castellanosii; P. chilensis; P. inerascens; P. denudans; P. elata; P. ferox; P. fiebrigii; P. flexousa; P. glandulosa; P. hassleri; P. humilis; P. juliflora; P. kuntzei; P. laevigata; P. nigra; P. pallida; P. palmeri; P. pubescens; P. reptans;*  *P. rojasiana; P. ruscifolia; P. sericantha; P. sericantha; P. strombulifera; P. tamarugo; P. torquata; P. velutina; P. vinalillo* |
| Iraq | *P. abbreviata; P. affinis; P. africana; P. alba; P. alpataco; P. argentina; P. articulata; P. caldenia; P. castellanosii; P. chilensis; P. cineraria; P. cinerascens; P. denudans; P. elata; P. ferox; P. flexousa; P. glandulosa; P. hassleri; P. juliflora; P. kuntzei; P. laevigata; P. nigra; P. pallida; P. palmeri; P. pubescens; P. reptans; P. rojasiana; P. ruscifolia; P. sericantha; P. strombulifera; P. tamarugo; P. torquata; P. velutina; P. vinalillo* |
| Israel | *P. abbreviata; P. affinis; P. africana; P. alba; P. alpataco; P. argentina; P. articulata; P. caldenia; P. castellanosii; P. chilensis; P. cineraria; P. cinerascens; P. denudans; P. elata; P. ferox; P. flexousa; P. glandulosa; P. hassleri; P. juliflora; P. kuntzei; P. laevigata; P. nigra; P. pallida; P. palmeri; P. pubescens; P. reptans; P. reptans; P. rojasiana; P. ruscifolia; P. sericantha; P. strombulifera; P. tamarugo; P. torquata; P. velutina; P. vinalillo* |
| Italy | *P. affinis; P. alba; P. algarobilla; P. alpataco; P. alpataco; P. caldenia; P. chilensis; P. cinerascens; P. denudans; P. elata; P. farcta; P. fiebrigii; P. flexousa; P. glandulosa; P. hassleri; P. humilis; P. juliflora; P. kuntzei; P. laevigata; P. nigra; P. pallida; P. pubescens; P. reptans; P. ruscifolia; P. sericantha; P. strombulifera; P. velutina* |
| Jamaica | *P. affinis; P. africana; P. alba; P. chilensis; P. cineraria; P. elata; P. fiebrigii; P. glandulosa; P. hassleri; P. juliflora; P. kuntzei; P. laevigata; P. nigra; P. pallida; P. ruscifolia; P. sericantha; P. vinalillo* |
| Japan | *P. affinis; P. alba; P. algarobilla; P. alpataco; P. caldenia; P. cinerascens; P. elata; P. fiebrigii; P. flexousa; P. glandulosa; P. hassleri; P. humilis; P. juliflora; P. kuntzei; P. laevigata; P. nigra; P. pallida; P. pubescens; P. reptans; P. ruscifolia; P. sericantha; P. strombulifera; P. velutina* |
| Jordan | *P. abbreviata; P. affinis; P. africana; P. alba; P. alpataco; P. argentina; P. articulata; P. caldenia; P. chilensis; P. cineraria; P. cinerascens; P. elata; P. ferox; P. flexousa; P. glandulosa; P. hassleri; P. juliflora; P. kuntzei; P. laevigata; P. nigra; P. pallida; P. palmeri; P. pubescens; P. reptans; P. reptans; P. rojasiana; P. ruscifolia; P. sericantha; P. strombulifera*; *P. torquata; P. torquata; P. velutina; P. vinalillo* |
| Kazakhstan | *P. abbreviata; P. affinis; P. africana; P. alba; P. alpataco; P. argentina; P. articulata; P. caldenia; P. chilensis; P. cineraria; P. cinerascens; P. elata; P. farcta; P. ferox; P. flexousa; P. glandulosa; P. hassleri; P. juliflora; P. kuntzei; P. laevigata; P. nigra; P. pallida; P. palmeri; P. pubescens; P. reptans; P. reptans; P. rojasiana; P. ruscifolia; P. sericantha; P. strombulifera; P. torquata; P. velutina; P. vinalillo* |
| Kenya | *P. abbreviata; P. affinis; P. alba; P. alpataco; P. argentina; P. articulata; P. caldenia; P. castellanosii; P. chilensis; P. cineraria*  *P. elata; P. farcta; P. ferox; P. fiebrigii; P. flexousa; P. glandulosa; P. hassleri; P. juliflora; P. kuntzei; P. laevigata; P. nigra; P. pallida; P. palmeri; P. pubescens; P. reptans; P. ruscifolia; P. sericantha; P. strombulifera; P. tamarugo; P. torquata;P. velutina;P. vinalillo* |
| Kuwait | *P. abbreviata; P. africana; P. alba; P. alpataco; P. argentina; P. articulata; P. cineraria;P. farcta; P. glandulosa; P. juliflora; P. laevigata; P. pallida; P. palmeri; P. pubescens; P. reptans; P. strombulifera; P. torquata; P. velutina* |
| Kyrgyzstan | *P. abbreviata; P. affinis; P. africana; P. alba; P. alpataco; P. argentina; P. articulata; P. caldenia; P. chilensis; P. cineraria; P. cinerascens; P. elata; P. farcta; P. ferox; P. flexousa; P. glandulosa; P. hassleri; P. juliflora; P. kuntzei; P. laevigata; P. nigra; P. pallida;P. palmeri; P. pubescens;P. reptans;P. reptans;P. rojasiana; P. ruscifolia; P. sericantha; P. strombulifera; P. torquata; P. velutina; P. vinalillo* |
| Laos | *P. affinis; P. africana; P. alba; P. alpataco; P. articulata; P. caldenia; P. campestris; P. chilensis; P. cineraria; P. elata; P. fiebrigii; P. flexousa; P. glandulosa; P. hassleri; P. juliflora; P. kuntzei; P. laevigata; P. nigra; P. pallida; P. palmeri; P. reptans; P. ruscifolia; P. ruscifolia; P. sericantha; P. torquata; P. vinalillo* |
| Lebanon | *P. chilensis; P. denudans; P. farcta; P. flexousa; P. glandulosa; P. juliflora; P. pubescens; P. strombulifera; P. velutina* |
| Lesotho | *P. alba; P. alpataco; P. articulata; P. chilensis; P. kuntzei; P. laevigata; P. pallida* |
| Liberia | *P. affinis; P. alba; P. chilensis; P. cineraria; P. elata; P. fiebrigii; P. glandulosa; P. hassleri; P. juliflora; P. kuntzei; P. laevigata; P. nigra; P. pallida; P. ruscifolia; P. sericantha; P. vinalillo* |
| Libya | *P. abbreviata; P. affinis; P. alba; P. alpataco; P. argentina; P. articulata; P. caldenia; P. castellanosii; P. chilensis; P. cineraria; P. cinerascens; P. denudans; P. elata; P. farcta; P. ferox; P. flexousa; P. glandulosa; P. hassleri; P. juliflora; P. kuntzei; P. laevigata; P. nigra; P. pallida; P. palmeri; P. pubescens; P. reptans; P. rojasiana; P. ruscifolia; P. sericantha; P. strombulifera; P. tamarugo; P. torquata; P. velutina; P. velutina; P. vinalillo* |
| Madagascar | *P. abbreviata; P. affinis; P. africana; P. alba; P. algarobilla; P. argentina; P. articulata; P. caldenia; P. campestris; P. castellanosii; P. chilensis; P. cineraria; P. cinerascens; P. elata; P. farcta; P. ferox; P. fiebrigii; P. flexousa; P. glandulosa; P. hassleri; P. humilis; P. juliflora; P. kuntzei; P. laevigata; P. nigra; P. pallida; P. palmeri;P. pubescens; P. reptans; P. ruscifolia; P. sericantha; P. strombulifera; P. tamarugo; P. torquata; P. velutina; P. vinalillo* |
| Malawi | *P. abbreviata; P. affinis; P. africana; P. alba; P. alpataco; P. argentina; P. articulata; P. caldenia; P. campestris; P. castellanosii; P. chilensis; P. cineraria; P. denudans; P. elata; P. farcta; P. ferox; P. fiebrigii; P. flexousa; P. glandulosa; P. hassleri; P. hassleri; P. juliflora; P. kuntzei; P. laevigata; P. nigra; P. pallida; P. palmeri; P. pubescens; P. reptans; P. ruscifolia; P. sericantha; P. strombulifera; P. tamarugo; P. torquata; P. velutina; P. vinalillo* |
| Macedonia | *P. affinis; P. alba; P. algarobilla; P. alpataco; P. caldenia; P. cinerascens; P. denudans; P. elata; P. fiebrigii; P. flexousa; P. glandulosa; P. hassleri; P. humilis; P. juliflora; P. kuntzei; P. laevigata; P. nigra; P. pallida; P. pubescens; P. reptans; P. ruscifolia; P. sericantha; P. strombulifera; P. velutina* |
| Malaysia | *P. affinis; P. chilensis; P. juliflora; P. kuntzei; P. pallida* |
| Mali | *P. abbreviata; P. affinis; P. alba; P. alpataco; P. argentina; P. articulata; P. caldenia; P. castellanosii; P. chilensis; P. cineraria; P. denudans; P. elata; P. farcta; P. ferox; P. fiebrigii; P. glandulosa; P. hassleri; P. juliflora; P. kuntzei; P. laevigata; P. nigra; P. pallida; P. palmeri; P. pubescens;P. reptans; P. ruscifolia;P. sericantha;P. strombulifera;*  *P. tamarugo; P. torquata; P. velutina; P. vinalillo* |
| Malta | *P. farcta; P. glandulosa; P. juliflora; P. pubescens; P. velutina* |
| Martinique | *P. juliflora; P. kuntzei* |
| Mauritania | *P. abbreviata; P. africana; P. alba; P. alpataco; P. argentina; P. articulata; P. caldenia; P. castellanosii; P. chilensis; P. cineraria;*  *P. denudans; P. farcta; P. ferox; P. flexousa; P. glandulosa; P. juliflora; P. laevigata; P. nigra; P. pallida; P. palmeri; P. pubescens; P. reptans; P. sericantha; P. strombulifera; P. tamarugo; P. torquata; P. velutina* |
| Mauritius | *P. juliflora; P. kuntzei* |
| Mayotte | *P. affinis; P. africana; P. alba; P. chilensis; P. cineraria; P. elata; P. fiebrigii; P. glandulosa; P. hassleri; P. juliflora; P. kuntzei; P. laevigata; P. nigra; P. pallida; P. ruscifolia; P. sericantha; P. vinalillo* |
| Mexico | *P. abbreviata; P. affinis; P. africana; P. alba; P. algarobilla; P. alpataco; P. argentina; P. caldenia; P. campestris; P. castellanosii; P. chilensis; P. cineraria; P. cinerascens; P. denudans; P. elata; P. farcta; P. ferox; P. fiebrigii; P. flexousa;P. hassleri; P. humilis; P. kuntzei; P. nigra; P. pallida; P. ruscifolia; P. sericantha; P. strombulifera; P. tamarugo; P. torquata;P. vinalillo* |
| Mongolia | *P. abbreviata; P. africana; P. alba; P. alpataco; P. argentina; P. articulata; P. cineraria; P. glandulosa; P. juliflora; P. laevigata; P. pallida; P. palmeri; P. pubescens; P. reptans; P. strombulifera; P. torquata; P. velutina* |
| Montserrat | *P. juliflora; P. kuntzei* |
| Montenegro | *P. affinis; P. alba; P. algarobilla; P. alpataco; P. caldenia; P. cinerascens; P. elata; P. fiebrigii; P. flexousa; P. glandulosa; P. hassleri; P. humilis; P. juliflora; P. kuntzei; P. laevigata; P. nigra; P. pallida; P. pubescens; P. reptans; P. ruscifolia; P. sericantha; P. strombulifera; P. velutina* |
| Morocco | *P. abbreviata; P. affinis; P. africana; P. alba; P. alpataco; P. argentina; P. articulata; P. caldenia; P. castellanosii; P. chilensis; P. cineraria; P. cinerascens; P. denudans; P. elata; P. farcta;*  *P. flexousa; P. glandulosa; P. hassleri; P. juliflora; P. kuntzei; P. laevigata; P. nigra; P. pallida; P. palmeri; P. palmeri; P. pubescens; P. reptans; P. rojasiana;*  *P. ruscifolia; P. sericantha; P. strombulifera; P. tamarugo; P. torquata; P. velutina; P. vinalillo* |
| Mozambique | *P alpataco; P. abbreviata; P. affinis; P. africana; P. alba; P. algarobilla; P. alpataco; P. argentina; P. articulata; P. caldenia;*  *P. campestris; P. castellanosii; P. chilensis; P. cineraria; P. cinerascens; P. denudans; P. elata; P. farcta; P. ferox; P. fiebrigii;*  *P. flexousa; P. glandulosa; P. hassleri; P. humilis; P. juliflora; P. kuntzei; P. laevigata; P. nigra; P. pallida; P. palmeri; P. pubescens; P. reptans; P. ruscifolia; P. sericantha; P. strombulifera; P. tamarugo; P. torquata; P. velutina; P. vinalillo* |
| Namibia | *P. abbreviata; P. affinis; P.africana; P. alba; P. alpataco; P. argentina; P. articulata; P. caldenia; P. castellanosii; P. chilensis;*  *P. cineraria; P. cinerascens; P. denudans; P. elata; P. farcta; P. ferox; P. flexousa; P. glandulosa; P. hassleri; P. juliflora; P. kuntzei; P. laevigata; P. nigra; P. pallida;*  *P. palmeri; P. pubescens; P. reptans; P. reptans; P. rojasiana; P. ruscifolia; P. sericantha; P. strombulifera; P. tamarugo; P. torquata; P. velutina; P. vinalillo* |
| Nepal | *P. alba; P. alpataco; P. articulata; P. caldenia; P. campestris; P. chilensis; P. flexousa; P. glandulosa; P. hassleri; P. juliflora; P. kuntzei; P. laevigata; P. nigra; P. pallida; P. palmeri; P. reptans; P. ruscifolia; P. sericantha; P. torquata* |
| Netherlands Antilles | *P. abbreviata; P. alpataco; P. argentina; P. caldenia; P. castellanosii; P. chilensis; P. denudans; P. farcta; P. ferox; P. flexousa; P. glandulosa; P. juliflora; P. kuntzei; P. laevigata; P. nigra; P. pubescens; P. sericantha; P. strombulifera; P. tamarugo; P. torquata; P. velutina* |
| New Caledonia | *P. affinis; P. africana; P. alba; P. algarobilla; P. alpataco; P. caldenia; P. chilensis; P. cineraria; P. cinerascens; P. elata;*  *P. fiebrigii; P. flexousa; P. glandulosa; P. hassleri; P. humilis;*  *P. juliflora; P. kuntzei; P. laevigata; P. nigra; P. pallida; P. pubescens; P. reptans; P. ruscifolia; P. sericantha ;P. strombulifera; P. vinalillo* |
| Nicaragua | *P. affinis; P. africana; P. alba; P. alpataco; P. articulata; P. chilensis; P. cineraria; P. elata; P. fiebrigii; P. glandulosa; P. hassleri; P. kuntzei; P. laevigata; P. nigra; P. pallida; P. ruscifolia; P. sericantha; P. vinalillo* |
| Niger | *P. abbreviata; P. alba; P. alpataco; P. argentina; P. articulata; P. caldenia; P. castellanosii; P. chilensis; P. cineraria; P. denudans; P. farcta; P. ferox; P. flexousa; P. glandulosa; P. juliflora; P. laevigata; P. nigra; P. pallida; P. palmeri; P. pubescens; P. reptans; P. sericantha; P. strombulifera; P. tamarugo; P. torquata; P. velutina* |
| Nigeria | *P. abbreviata; P. affinis; P. alba; P. alpataco; P. argentina; P. articulata; P. caldenia; P. castellanosii; P. chilensis; P. cineraria;*  *P. denudans; P. elata; P. farcta; P. ferox; P. fiebrigii ; P. glandulosa; P. hassleri; P. juliflora; P. kuntzei; P. laevigata; P. nigra; P. pallida; P. palmeri; P. pubescens; P. reptans; P. ruscifolia; P. sericantha; P. strombulifera; P. tamarugo; P. torquata; P. velutina; P. vinalillo* |
| Oman | *P. abbreviata; P. africana; P. alba; P. alpataco; P. argentina; P. articulata; P. farcta; P. glandulosa; P. juliflora; P. laevigata; P. pallida; P. palmeri; P. pubescens; P. reptans; P. strombulifera; P. torquata; P. velutina* |
| Pakistan | *P. abbreviata; P. affinis; P. africana; P. alba; P. algarobilla; P. alpataco; P. alpataco; P. argentina; P. articulata; P. caldenia;*  *P. castellanosii; P. chilensis; P. cinerascens; P. elata; P. farcta; P. ferox; P. fiebrigii; P. flexousa; P. glandulosa; P. hassleri; P. humilis; P. juliflora; P. kuntzei; P. laevigata; P. nigra; P. pallida; P. palmeri; P. pubescens; P. reptans; P. rojasiana; P. ruscifolia; P. sericantha; P. strombulifera; P. tamarugo; P. torquata; P. velutina; P. vinalillo* |
| Panama | *P. affinis; P. africana; P. alba; P. chilensis; P. cineraria; P. elata;*  *P. fiebrigii; P. glandulosa; P. hassleri; P. kuntzei; P. laevigata; P. nigra; P. pallida; P. ruscifolia; P. sericantha; P. vinalillo* |
| Papua New Guinea | *P. affinis; P. africana; P. alba; P. chilensis; P. cineraria; P. elata; P. fiebrigii; P. glandulosa; P. hassleri; P. juliflora; P. kuntzei; P. laevigata; P. nigra; P. pallida; P. ruscifolia; P. sericantha; P. vinalillo* |
| Paraguay | *P. abbreviata; P. affinis; P. africana; P. alba; P. algarobilla; P. alpataco; P. argentina; P. caldenia; P. castellanosii; P. chilensis; P. cineraria; P. cinerascens; P. denudans; P. elata; P. farcta; P. ferox; P. fiebrigii; P. flexousa; P. glandulosa; P. hassleri; P. humilis; P. juliflora; P. kuntzei; P. laevigata; P. nigra; P. pallida; P. pubescens; P. reptans; P. ruscifolia; P. sericantha; P. strombulifera; P. tamarugo; P. torquata; P. velutina; P. vinalillo* |
| Peru | *P. abbreviata; P. africana; P. alpataco; P. argentina; P. articulata; P. caldenia; P. castellanosii; P. cineraria; P. cinerascens; P. denudans; P. elata; P. farcta; P. ferox; P. fiebrigii; P. flexousa; P. glandulosa; P. hassleri; P. kuntzei; P. nigra; P. palmeri; P. pubescens; P. rojasiana; P. ruscifolia; P. sericantha; P. strombulifera; P. tamarugo; P. torquata; P. velutina; P. vinalillo* |
| Philippines | *P. affinis; P. africana; P. alba; P. articulata; P. chilensis;P. cineraria; P. elata; P. fiebrigii; P. glandulosa; P. hassleri; P. juliflora; P. kuntzei; P. laevigata; P. nigra; P. pallida; P. ruscifolia; P. sericantha; P. vinalillo* |
| Portugal | *P. alba; P. caldenia; P. campestris; P. chilensis; P. denudans; P. farcta; P. flexousa; P. glandulosa; P. hassleri; P. julifloraP. laevigata; P. nigra; P. pallida; P. palmeri;*  *P. pubescens; P. reptans; P. ruscifolia; P. sericantha; P. strombulifera; P. torquata; P. velutina* |
| Puerto Rico | *P. affinis; P. africana; P. alba; P. chilensis; P. cineraria; P. elata;*  *P. fiebrigii; P. glandulosa; P. hassleri; P. juliflora; P. kuntzei; P. laevigata; P. nigra; P. pallida; P. ruscifolia; P. sericantha; P. vinalillo* |
| Qatar | *P. abbreviata; P. africana; P. alba; P. alpataco; P. argentina;*  *P. articulata; P. glandulosa; P. juliflora; P. laevigata; P. pallida;*  *P. palmeri; P. pubescens; P.reptans; P. strombulifera; P. torquata; P. velutina; P. farcta* |
| Reunion | *P. affinis; P. africana; P. alba; P. algarobilla; P. caldenia; P. chilensis; P. cineraria; P. cinerascens; P. elata; P. fiebrigii; P. flexousa; P. glandulosa; P. hassleri; P. humilis; P. juliflora; P. kuntzei; P. laevigata; P. nigra; P. pallida; P. pubescens; P. reptans; P. ruscifolia; P. sericantha; P. strombulifera; P. velutina; P. vinalillo* |
| Romania | *P. abbreviata; P. affinis; P. africana; P. alba; P. algarobilla; P. alpataco; P. alpataco; P. argentina; P. articulata; P. caldenia;*  *P. cineraria; P. cinerascens; P. elata; P. fiebrigii; P. flexousa;*  *P. glandulosa; P. hassleri; P. humilis; P. juliflora; P. kuntzei; P. laevigata; P. nigra; P. pallida; P. palmeri; P. pubescens;*  *P. reptans; P. ruscifolia; P. sericantha; P. strombulifera; P. torquata; P. velutina* |
| Russia | *P. abbreviata; P. affinis; P. africana; P. alba; P. algarobilla; P. alpataco; P. argentina; P. articulata; P. caldenia; P. cineraria;*  *P. cinerascens; P. elata; P. fiebrigii; P. flexousa; P. glandulosa;*  *P. hassleri; P. humilis; P. kuntzei; P. laevigata; P. nigra; P. pallida; P. palmeri; P. pubescens; P. reptans; P. ruscifolia; P. sericantha; P. strombulifera; P. torquata; P. velutina* |
| Rwanda | *P. affinis; P. africana; P. albaP. articulata; P. chilensis; P. cineraria; P. elata; P. fiebrigii; P. flexousa; P. glandulosa; P. hassleri; P. juliflora; P. kuntzei; P. laevigata; P. nigra; P. pallida; P. ruscifolia; P. sericantha; P. strombulifera; P. velutina; P. vinalillo* |
| Saint Lucia | *P. juliflora; P. kuntzei* |
| Sao Tome and Principe | *P. affinis; P. africana; P. alba; P. chilensis; P. cineraria; P. elata;*  *P. fiebrigii; P. glandulosa; P. hassleri; P. juliflora; P. kuntzei; P. laevigata; P. nigra; P. pallida; P. ruscifolia; P. sericantha; P. vinalillo* |
| Saudi Arabia | *P. abbreviata; P. affinis; P. africana; P. alba; P. alpataco; P. argentina; P. articulata; P. caldenia; P. castellanosii; P. chilensis*  *P. cinerascens; P. denudans; P. elata; P. ferox; P. flexousa;*  *P. glandulosa; P. hassleri; P. juliflora; P. kuntzei; P. laevigata;*  *P. nigra; P. pallida; P. palmeri; P. pubescens; P. reptans; P. reptans; P. rojasiana; P. ruscifolia; P. sericantha; P. strombulifera; P. tamarugo; P. torquata; P. velutina;*  *P. vinalillo* |
| Senegal | *P. abbreviata; P. affinis; P. alba; P. alpataco; P. argentina;*  *P. articulata; P. caldenia; P. castellanosii; P. chilensis; P. cineraria; P. denudans; P. elata; P. farcta; P. ferox; P. fiebrigii;*  *P. flexousa; P. glandulosa; P. hassleri; P. juliflora; P. kuntzei; P. laevigata; P. pallida; P. palmeri; P. pubescens; P. reptans; P. ruscifolia; P. sericantha; P. trombulifera; P. tamarugo; P. torquata; P. velutina; P. vinalillo* |
| Serbia | *P. affinis; P. alba; P. algarobilla; P. alpataco; P. caldenia; P. cinerascens; P. elata; P. fiebrigii; P. flexousa; P. glandulosa; P. hassleri; P. humilis; P. juliflora; P. kuntzei; P. laevigata; P. nigra; P. pallida; P. pubescens; P. reptans; P. ruscifolia; P. sericantha; P. strombulifera; P. velutina* |
| Sierra Leone | *P. affinis; P. alba; P. chilensis; P. cineraria; P. elata; P. fiebrigii; P. glandulosa; P. hassleri; P. juliflora; P. kuntzei; P. laevigata; P. nigra; P. pallida; P. ruscifolia; P. sericantha; P. vinalillo* |
| Slovenia | *P. affinis; P. alba; P. algarobilla; P. alpataco; P. caldenia; P. cinerascens; P. elata; P. fiebrigii; P. flexousa; P. glandulosa; P. hassleri; P. humilis; P. juliflora; P. kuntzei; P. laevigata; P. nigra; P. pallida; P. pubescens; P. reptans; P. ruscifolia; P. sericantha; P. strombulifera;P. velutina* |
| Solomon Islands | *P. juliflora; P. kuntzei* |
| Somalia | *P. abbreviata; P. affinis; P. africana; P. alba; P. alpataco; P. argentina; P. articulata; P. chilensis; P. cineraria; P. elata; P. farcta; P. fiebrigii; P. glandulosa; P. hassleri; P. juliflora; P. kuntzei; P. laevigata; P. nigra; P. pallida; P. palmeri;*  *P. pubescens; P. reptans; P. ruscifolia; P. sericantha; P. strombulifera; P. torquata; P. velutina; P. vinalillo* |
| South Africa | *P. abbreviata; P. affinis; P. africana; P. alba; P. algarobilla; P. alpataco; P. argentina; P. articulata; P. caldenia; P. campestris; P. castellanosii; P. chilensis; P. cineraria; P. cinerascens; P. denudans; P. elata; P. farcta; P. ferox; P. fiebrigii; P. flexousa; P. glandulosa; P. hassleri; P. humilis; P. juliflora; P. kuntzei; P. laevigata; P. nigra; P. pallida; P. palmeri; P. pubescens; P. reptans; P. rojasiana; P. ruscifolia; P. sericantha; P. strombulifera; P. tamarugo; P. torquata; P. velutina; P. vinalillo* |
| South Korea | *P. affinis; P. alba; P. algarobilla; P. alpataco; P. caldenia; P. caldenia; P. campestris; P. cinerascens; P. elata; P. fiebrigii; P. flexousa; P. glandulosa; P. hassleri; P. humilis; P. juliflora; P. kuntzei; P. laevigata; P. nigra; P. pallida; P. palmeri; P. pubescens; P. reptans; P. reptans; P. ruscifolia; P. sericantha; P. strombulifera; P. torquata; P. velutina* |
| Spain | *P. abbreviata; P. affinis; P. africana; P. alba; P. algarobilla; P. alpataco; P. argentina; P. articulata; P. caldenia; P. castellanosii;*  *P. chilensis; P. cineraria; P. cinerascens; P. denudans; P. elata;*  *P. farcta; P. ferox; P. fiebrigii; P. flexousa; P. glandulosa; P. hassleri; P. humilis; P. juliflora; P. kuntzei; P. laevigata; P. nigra;*  *P. pallida; P. palmeri; P. pubescens; P. reptans; P. ruscifolia; P. sericantha; P. strombulifera; P. tamarugo; P. torquata; P. velutina* |
| Sri Lanka | *P. affinis; P. africana; P. alba; P. chilensis; P. chilensis; P. cineraria; P. elata; P. fiebrigii; P. glandulosa; P. hassleri; P. juliflora; P. kuntzei; P. laevigata; P. nigra; P. pallida; P. ruscifolia; P. sericantha; P. vinalillo* |
| Sudan | *P. abbreviata; P. affinis; P. alba; P. alpataco; P. argentina; P. articulata; P. caldenia; P. caldenia; P. campestris; P. castellanosii; P. chilensis; P. cineraria; P. denudans; P. elata; P. farcta; P. ferox; P. fiebrigii; P. flexousa; P. glandulosa; P. hassleri;*  *P. juliflora; P. kuntzei; P. laevigata; P. nigra; P. pallida; P. palmeri; P. pubescens; P. reptans; P. ruscifolia; P. sericantha; P. strombulifera; P. tamarugo; P. torquata; P. velutina; P. vinalillo* |
| Suriname | *P. affinis; P. africana; P. alba; P. chilensis; P. cineraria; P. elata;*  *P. fiebrigii; P. glandulosa; P. hassleri; P. juliflora; P. kuntzei; P. laevigata; P. nigra; P. pallida; P. ruscifolia; P. sericantha; P. vinalillo* |
| Swaziland | *P. abbreviate; P. affinis; P. alba; P. argentina; P. caldenia; P. caldenia; P. campestris; P. chilensis;P. cinerascens; P. elata; P. farcta; P. ferox; P. fiebrigii; P. flexousa; P. glandulosa; P. hassleri; P. humilis; P. juliflora; P. kuntzei; P. laevigata; P. nigra; P. pallida;*  *P. palmeri; P. pubescens; P. reptans; P. ruscifolia; P. sericantha*  *P. strombulifera; P. tamarugo; P. torquata; P. velutina* |
| Syria | *P. abbreviata; P. africana; P. alba; P. alpataco; P. argentina; P. articulata; P. caldenia; P. caldenia; P. castellanosii; P. chilensis;*  *P. cineraria; P. denudans; P. ferox; P. flexousa; P. glandulosa;*  *P. juliflora; P. laevigata; P. nigra; P. pallida; P. palmeri; P. pubescens; P. reptans; P. sericantha; P. strombulifera; P. tamarugo; P. torquata; P. velutina* |
| Taiwan | *P. affinis; P. africana; P. alba; P. algarobilla; P. articulata; P. caldenia; P. campestris; P. chilensis; P. cineraria; P. cinerascens; P. elata; P. fiebrigii; P. flexousa; P. glandulosa; P. hassleri; P. humilis; P. juliflora; P. kuntzei; P. laevigata; P. nigra; P. pallida; P. palmeri; P. pubescens; P. reptans; P. ruscifolia; P. sericantha; P. strombulifera; P. torquata; P. velutina; P. vinalillo* |
| Tajikistan | *P. abbreviata; P. affinis; P. africana; P. alba; P. alpataco; P. argentina; P. articulata; P. caldenia; P. chilensis; P. cineraria; P. cinerascens; P. elata; P. ferox; P. flexousa; P. glandulosa; P. hassleri; P. juliflora; P. kuntzei; P. laevigata; P. nigra; P. pallida; P. palmeri; P. pubescens; P. reptans; P. rojasiana; P. ruscifolia; P. sericantha; P. strombulifera; P. torquata; P. velutina; P. vinalillo* |
| Tanzania | *P. abbreviata; P. affinis; P. alba; P. algarobilla; P. argentina; P. articulata; P. caldenia; P. campestris; P. castellanosii; P. chilensis; P. cineraria; P. cinerascens; P. elata; P. farcta; P. ferox; P. fiebrigii; P. flexousa; P. glandulosa; P. hassleri; P. humilis; P. juliflora; P. kuntzei; P. laevigata; P. nigra; P. pallida; P. palmeri; P. pubescens; P. reptans; P. ruscifolia; P. sericantha; P. strombulifera; P. tamarugo; P. torquata; P. velutina; P. vinalillo* |
| Thailand | *P. affinis; P. africana; P. alba; P. caldenia; P. campestris; P. chilensis; P. cineraria; P. elata; P. fiebrigii; P. flexousa; P. glandulosa; P. hassleri; P. juliflora; P. kuntzei; P. laevigata; P. nigra; P. pallida; P. palmeri; P. reptans; P. ruscifolia; P. sericantha; P. torquata; P. vinalillo* |
| Timor-Leste | *P. affinis; P. africana; P. alba; P. alpataco; P. articulata; P. chilensis; P. cineraria; P. elata; P. fiebrigii; P. glandulosa; P. hassleri; P. juliflora; P. kuntzei; P. laevigata; P. nigra; P. pallida; P. ruscifolia; P. sericantha; P. vinalillo* |
| Togo | *P. affinis; P. alba; P. chilensis; P. cineraria; P. elata; P. fiebrigii; P. glandulosa; P. hassleri; P. juliflora; P. kuntzei; P. laevigata; P. nigra; P. pallida; P. ruscifolia; P. sericantha; P. vinalillo* |
| Trinidad and Tobago | *P. affinis; P. africana; P. alba; P. chilensis; P. cineraria; P. elata; P. fiebrigii; P. glandulosa; P. hassleri; P. juliflora; P. kuntzei; P. laevigata; P. nigra; P. pallida; P. ruscifolia; P. sericantha; P. vinalillo* |
| Tunisia | *P. abbreviata; P. affinis; P. africana; P. alba; P. alpataco; P. argentina; P. articulata; P. caldenia; P. castellanosii; P. chilensis;*  *P. cineraria; P. cinerascens; P. denudans; P. elata; P. ferox; P. flexousa; P. glandulosa; P. hassleri; P. juliflora; P. kuntzei; P. laevigata; P. nigra; P. pallida; P. palmeri;*  *P. pubescens; P. reptans; P. rojasiana; P. ruscifolia; P. sericantha; P. strombulifera; P. tamarugo; P. torquata; P. velutina; P. vinalillo* |
| Turkey | *P. abbreviata; P. affinis; P. africana; P. alba; P. algarobilla; P. alpataco; P. argentina; P. articulata; P. caldenia; P. chilensis; P. cineraria; P. cinerascens; P. denudans; P. elata; P. fiebrigii; P. flexousa; P. glandulosa; P. hassleri; P. humilis; P. juliflora; P. kuntzei; P. laevigata; P. nigra; P. pallida; P. palmeri; P. pubescens; P. reptans; P. ruscifolia; P. sericantha; P. strombulifera; P. torquata; P. velutina* |
| Turkmenistan | *P. abbreviata; P. affinis; P. africana; P. alba; P. alpataco; P. argentina; P. articulata; P. caldenia; P. chilensis; P. cineraria; P. cinerascens; P. elata; P. ferox; P. flexousa; P. glandulosa; P. hassleri; P. juliflora; P. kuntzei; P. laevigata; P. nigra; P. pallida; P. palmeri; P. pubescens; P. reptans; P. rojasiana; P. ruscifolia; P. sericantha; P. strombulifera; P. torquata; P. velutina; P. vinalillo* |
| Turks and Caicos Islands | *P. affinis; P. africana; P. alba; P. chilensis; P. cineraria; P. elata; P. fiebrigii; P. glandulosa; P. hassleri; P. juliflora; P. kuntzei; P. laevigata; P. nigra; P. pallida; P. ruscifolia; P. sericantha; P. vinalillo* |
| Uganda | *P. affinis; P. alba; P. chilensis; P. cineraria; P. elata;P. fiebrigii; P. flexousa; P. glandulosa; P. hassleri; P. juliflora; P. kuntzei; P. laevigata; P. nigra; P. pallida; P. ruscifolia; P. sericantha; P. strombulifera; P. velutina; P. vinalillo* |
| Ukraine | *P alpataco; P. affinis; P. alba; P. algarobilla; P. caldenia; P. cinerascens; P. elata; P. fiebrigii; P. flexousa; P. glandulosa; P. hassleri; P. humilis; P. kuntzei; P. laevigata; P. nigra;*  *P. pallida; P. pubescens; P. reptans; P. ruscifolia; P. sericantha; P. strombulifera; P. velutina* |
| United Arab Emirates | *P. abbreviata; P. africana; P. alba; P. alpataco; P. argentina; P. articulata; P. glandulosa; P. juliflora; P. laevigata; P. pallida; P. palmeri; P. pubescens; P. reptans; P. strombulifera; P. torquata;*  *P. velutina* |
| United Kingdom | *P. farcta; P. glandulosa; P. juliflora; P. pubescens; P. velutina* |
| United States | *P. abbreviata; P. affinis; P. africana; P. alba; P. algarobilla; P. alpataco; P. argentina; P. caldenia; P. castellanosii; P. cineraria;*  *P. denudans; P. elata; P. farcta; P. ferox; P. fiebrigii; P. flexousa;*  *P. hassleri; P. humilis; P. juliflora; P. kuntzei; P. nigra; P. pallida; P. palmeri; P. reptans; P. rojasiana; P. ruscifolia; P. sericantha; P. tamarugo; P. torquata; P. vinalillo* |
| Uruguay | *P. algarobilla; P. cinerascens; P. elata;P. fiebrigii; P. flexousa; P. glandulosa; P. hassleri; P. humilis; P. juliflora; P. kuntzei; P. laevigata; P. nigra; P. pallida; P. pubescens; P. reptans; P. sericantha; P. strombulifera; P. velutina* |
| Uzbekistan | *P. abbreviata; P. affinis; P. africana; P. alba; P. alpataco; P. argentina; P. articulata; P. caldenia; P. chilensis; P. cineraria;*  *P. cinerascens; P. elata; P. farcta; P. ferox; P. flexousa; P. glandulosa; P. hassleri; P. juliflora; P. kuntzei; P. laevigata; P. nigra; P. pallida; P. palmeri; P. pubescens; P. reptans; P. rojasiana; P. ruscifolia; P. sericantha; P. strombulifera;*  *P. torquata; P. velutina; P. vinalillo* |
| Vanuatu | *P. affinis; P. chilensis; P. juliflora; P. juliflora; P. kuntzei; P. pallida* |
| Venezuela | *P. abbreviata; P. affinis; P. africana; P. alba; P. alpataco; P. argentina;P. caldenia; P. castellanosii; P. chilensis; P. cineraria; P. elata; P. farcta; P. ferox; P. fiebrigii P. glandulosa; P. hassleri; P. kuntzei; P. laevigata; P. nigra;*  *P. pallida; P. pubescens; P. ruscifolia; P. sericantha; P. strombulifera; P. tamarugo; P. torquata; P. velutina;*  *P. vinalillo* |
| Vietnam | *P. affinis; P. africana; P. alba; P. alpataco; P. articulata; P. caldenia; P. campestris; P. chilensis; P. cineraria; P. elata; P. fiebrigii; P. flexousa; P. glandulosa; P. hassleri; P. juliflora; P. kuntzei; P. laevigata; P. nigra; P. pallida; P. palmeri; P. reptans; P. ruscifolia; P. sericantha; P. torquata; P. vinalillo* |
| West Bank | *P. abbreviata; P. africana; P. alba; P. alpataco; P. argentina; P. articulata; P. caldenia; P. castellanosii; P. chilensis; P. cineraria;*  *P. denudans; P. farcta; P. ferox; P. flexousa; P. glandulosa; P. juliflora; P. laevigata; P. nigra; P. pallida; P. palmeri;*  *P. pubescens; P. reptans; P. sericantha; P. strombulifera; P. tamarugo; P. torquata; P. velutina* |
| Western Sahara | *P. abbreviata; P. africana; P. alba; P. alpataco; P. argentina; P. articulata; P. cineraria; P. farcta; P. glandulosa; P. juliflora; P. laevigata; P. pallida; P. palmeri; P. pubescens; P. reptans; P. strombulifera; P. torquata; P. velutina* |
| Yemen | *P. abbreviata; P. affinis; P. africana; P. alba; P. alpataco; P. argentina; P. articulata; P. caldenia; P. castellanosii; P. chilensis*  *P. cinerascens; P. denudans; P. elata; P. ferox; P. flexousa; P. glandulosa; P. hassleri; P. juliflora; P. kuntzei; P. laevigata; P. nigra; P. pallida; P. palmeri; P. pubescens; P. reptans; P. rojasiana; P. ruscifolia; P. sericantha; P. strombulifera; P. tamarugo; P. torquata; P. velutina; P. vinalillo* |
| Zambia | *P. abbreviata; P. affinis; P. africana; P. alba; P. alpataco; P. argentina; P. articulata; P. caldenia; P. campestris; P. castellanosii; P. chilensis; P. cineraria; P. denudans; P. elata*  *P. farcta; P. ferox; P. fiebrigii; P. flexousa; P. glandulosa; P. hassleri; P. juliflora; P. kuntzei; P. laevigata; P. nigra;*  *P. pallida; P. palmeri; P. pubescens; P. reptans; P. ruscifolia; P. sericantha; P. strombulifera; P. tamarugo; P. torquata; P. velutina; P. vinalillo* |
| Zimbabwe | *P. abbreviata; P. affinis; P. africana; P. alba; P. alpataco; P. argentina; P. articulata; P. caldenia; P. campestris; P. castellanosii; P. chilensis; P. cineraria; P. denudans; P. elata; P. farcta; P. ferox; P. fiebrigii; P. flexousa; P. glandulosa; P. hassleri;*  *P. juliflora; P. kuntzei; P. laevigata; P. nigra; P. pallida; P. palmeri; P. pubescens; P. reptans; P. ruscifolia; P. sericantha; P. strombulifera; P. tamarugo; P. torquata; P. velutina; P. vinalillo* |
