# Supplementary material for: Prosopis: a global assessment of the biogeography, benefits, impacts and management of one of the world's worst woody invasive plant taxa
Source: AoB Plants. 2014 Jun 4;6:plu027. doi: 10.1093/aobpla/plu027 (PMC4086457; doi:10.1093/aobpla/plu027)
Supplement: Additional Information [file supp_plu027_plu027supp_file4.doc]

| **­­­Country/**  **Territory** | **Introduction dates** | **Source of introduction** | **Reason for introduction** | **Uses** | **Use level (zero, low, moderate, high)** | **Impacts** | **Evidence of impacts (zero, low, moderate, high)** | **Scale of invasion (area or region)** | **Control operations** | **No publications** | **Level of evidence understanding (just occurrence, very low, low, moderate, good, excellent)** | **References** |
| --- | --- | --- | --- | --- | --- | --- | --- | --- | --- | --- | --- | --- |
| Ascension Island | 1960s | ? | Consolidate ground where a village was being constructed | none | zero | Impacts turtle and seabird nesting sites, impacts native flora and supports vermin, | moderate | Large area of island - approximately 53 km² | Have a sound strategic plan set up, biocontrol, mechanical and chemical control especially at sea turtle nesting sites | 3 | good | Pickup, 1999; Belton, 2008; Miller and Darlow, 2008 |
| Afghanistan | ? | ? | ? | Fuelwood | low | Invades fields | zero | ? | no | 1 | low | Sohrabi et al. 2011 |
| Algeria | ? | ? | ? | ? | zero | ? | zero | ? | no | 1 | just occurrence | Pasiecznick et al. 2001; |
| Argentina | N/A | N/A | N/A | Useful agroforestry species – fuelwood and timber | high | Encroaches native rangelands, decreasing grazing potential | moderate | 33 000 km² increasing abundance ( *P. caldenia*) | Small scale control programs (mechanical and chemical control) also suggested that burning and grazing management can be beneficial to control its spread and is used in some areas (cultural control) | >10 | good | Lasalle, 1962; Distel et al. 1996; Dussart et al. 1997; Peinetti et al. 1997; Medina et al. 2000; de Villalobos et al. 2005a; de Villalobos et al. 2005b; Villagra et al. 2005; de Villalobos et al. 2007; Bogino and Villalba, 2008; Busch et al. 2012; Cardoso et al. 2012; Lauenstein et al. 2013. |
| Australia | Early 1900s | Hawaii or Pacific Islands, USA, South Africa | Ornamental, shade and fodder | Ornamental, shade and fodder especially opportunistic grazing by cattle | zero | Erosion, reduces grazing potential, impacts water supply, supports feral animals, damage to vehicles; impacts local vegetation | high | Widespread between 0.5 and 1 million ha and could invade up to 70 % of the country, number of patches in a 450 ha study area more than doubled over 31 years, spread of 0.71 % per year | Biocontrol (2 seed beetles, a leaf tying moth and a sap-sucker) mechanical, chemical and fire control programmes, fines for non-compliance, animal management control (7day period before transport) good strategic plan | >10 | excellent | Panetta and Carstairs, 1989; Csurhes, 1996; Lynes and Campbell, 2000; Pasiecznik et al. 2001; National Weeds Strategy Executive Committee, 2001; Osmond, 2003; van Kilnken and Burwell, 2005; Anderson et al. 2006; Page and Lacey, 2006; van Klinken et al. 2006; van Klinken et al. 2007; Robinson et al. 2008; van Klinken and White, 2011; Northern Territory Government, 2012; van Klinken, 2012 |
| Bahrain | 1920-1930 | Hawaii | Soil stabilisation | ? | zero | ? | zero | ? | no | 1 | low | Pasiecznick et al. 2001 |
| Bangladesh | ? | ? | ? | ? | zero | ? | zero | ? | No – suggested that further introductions would be good | 1 | just occurrence | Pasiecznick et al. 2001; Hasan and Alam, 2006 |
| Botswana | ? | Presumed accidental from South Africa or Namibia | ? | Fuelwood, fodder | low | Blocks boreholes and water ways, water uptake, depletes ground water, hiding place for criminals, increased car accidents because of lower visibility | moderate | Large areas invaded in the southern and western parts of the country – exact areas not knows | Monitoring spread – no formal control | 2 | moderate | Botswana Government, 2009; Muzila et al.2011 |
| Brazil | 1942; 1947; 1948 | USA; Peru; Sudan | Agroforestry | Source of pollen for honey; fuelwood and fodder | moderate | Less native species in invaded areas, impacts on native communities, also promotes the presence of other invasive; impacts cattle and goat health; impacts soil and microbes negatively | good | Wide spread over 1 million ha (*P. juliflora*) and small populations of  *P. ruscifolia* | No formal control; recent paper highlighting the need for control | 7 | good | Pasiecznik et al. 2001; De Andrade et al. 2010; Leão et al. 2011; Matanda wet al. 2011; de Olivera et al. 2012 |
| Burkina Faso | Accidental mid-1900, purposeful 1986 | Peru, Chile, Mexico and  probably Senegal, Honduras | Combat desertification - agroforestry | ? | zero | ? | zero | ? | no | 2 | low | Pasiecznick et al. 2001; Ræbild et al. 2003 |
| Cape Verde | 1950s-1970s (multiple) | Trinidad, Arizona, India, Chile | Afforestation, soil stabilisation | Fuelwood, fodder, timber, erosion control, | moderate | Ground water uptake | low | Wide spread | no | 3 | moderate | FAO, 2004; AFTD, 2009; Cienciala et al. 2013 |
| Chad | ? | ? | Fuelwood, afforestation, soil stabilization, improving degraded areas | ? | zero | ? | zero | Wide spread (more than 100 000 ha) | no | 2 | low | Pasiecznik et al. 2001; ISSG, 2005; Geesing et al. 2004 |
| Chile | N/A | N/A | N/A | Agroforestry, fuelwood, fodder, food sources | high | none | zero | ? | no | 1 | low | Pasiecznick et al. 2001 |
| Columbia | N/A | N/A | N/A | Agroforestry | high | ? | zero | ? | no | 1 | low | Pasiecznick et al. 2001 |
| Cote D’Ivore | ? | ? | ? | ? | zero | ? | zero | ? | no | 1 | just occurrence | Pasiecznick et al. 2001 |
| Djibouti | ? | ? | ? | ? | zero | ? | zero | ? | Control through utilisation | 1 | just occurrence | Pasiecznick et al. 2001 |
| Egypt | 1900 |  |  |  |  | Reduces ground water, impacts indigenous species, impacts grazing encroaching in protected areas |  | Prominent at one site it is now up to 120 km form original sources | Biocontrol –two seed bruchids- not purposefully introduced, small scale community projects doing mechanical and chemical clearing in protected areas | 2 | moderate | Pasiecznik et al. 2001;Ghazali, 2006; |
| Eretria | 1970s | Probably Sudan | Accidental | Fodder and fuelwood (approximately 10 % of households use it | low | Invading protected areas, health problems for animals and people, refuge for wild animals, impacts grazing and cropping lands, reduces water supply, impacting native tree species | good | Approximately 1 million ha (spread of 50 00 ha per year) | Spent over 31 million Eritrean birr on mechanical and chemical control in the past and producing a national strategy and action plan - using control through utilisation as main approach now | 2 | good | Pasiecznick et al. 2001; Bokrezion 2008 |
| Ethiopia | 1972 | India and accidently from Kenya and or Sudan | Fuelwood, fencing, fodder and wind breaks | Fuelwood, fencing, fodder and wind breaks | moderate | Biodiversity impacts, grazing impacts, injuries to humans and livestock, encroachment of infrastructure, exerting extra finical burden on local communities | good | Large scale – continuous for over 400 km – over 1.2 million ha | Acknowledge control is needed, Discussing biocontrol. FARM Africa set control through utilisation projects up with in the country | >10 | good | ISSG, 2005; Berhanu and Tesfay, 2006; Ethiopia Government, 2002; Shiferaw et al. 2004; Berhanu and Tesfaye, 2008; Admasu, 2006; FARM-Africa, 2008;Steele et al. 2008; Demessie, 2009; Seid, 2012; Tessema, 2012 |
| Gambia | ? | ? | ? | ? | zero | ? | zero | ? | no | 1 | just occurrence | Pasiecznick et al. 2001 |
| Georgia | N/A | N/A | N/A | ? | zero | ? | zero | ? | no | 1 | just occurrence | Pasiecznick et al. 2001 |
| Ghana | ? | ? | ? | ? | zero | ? | zero | ? | no | 1 | just occurrence | Pasiecznick et al. 2001 |
| Guinea-Bissau | ? | ? | ? | ? | zero | ? | zero | ? | no | 1 | just occurrence | AFTD, 2009 |
| Haiti | Multiple 1900-1930s | USA and Indi or South Africa | silviculture | Fuelwood and fodder (meets 70 % of fuelwood needs) | high | ? | zero | common | no | 4 | low | Pasiecznick et al. 2001; Lee et al. 1992; Timyan, 1996 |
| Hawaii | 1828 | Peru, Brazil via France | agroforestry | Honey, erosion control, fodder | moderate | Impacts native species richness and soil chemistry; uptake of ground water, reduces grazing land | good | Widespread 60700 ha and present on all islands; potential to spread over 350 000 ha | Mechanical and chemical control ,and monitoring fairly large scale work in conservation area (multiple biocontrol most introduced some unintentionally introduced in the early 1900s | 6 | good | Skolmen, undated; Emlen ,1986; Hawaii Ecosystem at Risk Project, 1998; Pasiecznick et al. 2001; Gallaber and Merlin, 2010; Kaur et al. 2012 |
| India | 1857; 1876; 1912; 1913; 1915larg scale planting and distribution in the 1930s | Jamaica; Mexico; Peru, Argentina and Uruguay; Australia | Fuelwood ; soil rehabilitation; charcoal making important source of income up to 79% for many households due to benefits declared a royal plant in 1940; some towns have *Prosopi*s as sacred treed, human food, honey production | Fodder, only about 5 % , fuelwood (up to 89% of households where it is invasive – in general still heavy utilization | High | Impacting native species richness, and grazing capacity in national parks thereby impacting tiger populations- causing local conflicts between people and managers; degradation of land and grazing potential, impacting livelihoods; encroaching protected areas; groundwater depletion; impacts on bird biodiversity in urban areas; impacting national parks; agricultural abandonment, effecting biodiversity; Changes diazotrophic diversity in the rhizosphere; alters soil characteristics; encroaching national parks, impacting black buck populations, alters animal interactions | good | Widespread through the country – from 378km² in 1980 to 684 km² in 1992  5.5 million ha in total (on site planted on 31500 ha and has spread to over 2 million ha in Gujarat – spread at approximately 25 km² p/a) | Various control- exploitation/value addition (control through utilisation), very small scale mechanical clearing at Localised control areas e.g. National Parks, lots of experimental control trials – local community eradication mainly to reclaim land for agriculture; clearing in some national parks | >10 | excellent | Chandrasekaran and Swamy , unsated; Jhala, 1993; Sharma and Dakshini, 1998; Pasiecznick et al. 2001; Ibrahim, 2004; Pasiecznick et al. 2004; FAO, 2006; Dayal, 2007; AFTD, 2009; Chikuni et al. 2009; Reddy et al. 2009; Bhatt et al. 2011; Walter, 2011; Cibichakravarthy et al. 2012; Joshi et al. 2012; Kaur et al. 2012; Pandy et al. 2012; Singh and Shkla, 2012; Naseeruddin et al. 2013; Sato, 2013 |
| Iran | 1950s | ? | Dune stabilisation | ? | low | Weedy in agricultural areas | low | 25 000 ha | No management, but possible promotion of the species for recovering soil in Iran | 3 | moderate | Pasiecznik et al. 2001; AFTD, 2009; Sohribi et al. 2011; Sajad and Sefidi, 2012. |
| Iraq | 1950s | ? | Dune stabilisation, local greening | Dune stabilisation and ornamental | low | ? | zero | ? | no | 1 | low | Berhanu and Tesfay, 2006 |
| Israel | 1970s | Mexico | ? | Invades agricultural fields | low | ? | zero | Low invasion | no | 1 | low | Pasiecznick et al. 2001; |
| Jordan | 1950s | ? | ? | Dune stabilisation, local greening | zero | Encroaches croplands – impenetrable thickets – also encroaches water related infrastructure – refuge for parasites (*Cuscuta* spp) and hosts white fly which impacts agricultural crops | moderate | ? | Mechanical and chemical control - fairly unsuccessful. Found Carydon palaestinicus insect eating seeds – experimental sites for herbicide control | 2 | moderate | Pasiecznik et al. 2001; Quasem, 2007 |
| Kenya | Officially in 1973 however it is suspected that it was accidently introduced from Sudan earlier | Hawaii | To rehabilitate degraded areas and to improve fuelwood and fodder supply | Still over 50 % of people using it for poles, fuelwood and fodder accounting for up to 1600 Kdh per year per household | high | Impacts on human and animal health, punctures, impacts native vegetation, soil, agriculture – causing legal conflicts, average a decreased yield of 600kg of maize per household per year; seen to increase presence of malaria, causes drainage problem and cracks ground impacts soils and herbaceous plants | good | Spread approximately 50 km from planted sites, has now has invades over 300 km² potential to spread from 3 up to 27.7 million ha – approximately 739.3 ha of initial planting | Control through utilisation | >10 | excellent | Andersson, 2005; Choge et al. 2002; Lemlem, 2003; Choge and Chikami, 2004; Mwangi and Swallow, 2005; Choge et al. 2006; FAO 2006; Pasiecznik et al. 2006; Choge et al. 2007; Kahi et al. 2009; Muturi et al. 2009; Mworia et al. 2011; Choge et al. 2012; Njoroge et al. 2012; Maundu et al. 2013; Muturi et al. 2013; |
| Kuwait | 1950s | ? | ? | ? | zero | ? | zero | ? | no | 1 | just occurrence | Pasiecznick et al. 2001 |
| Libya | ? | ? | ? | ? | zero | ? | zero | ? | no | 1 | just occurrence | Pasiecznick et al. 2001 |
| Mali | 1980s |  | Combat desertification and stabilisation | *Prosopis* is seen to be facilitating natural forest growth – less browsing pressure and improved soil, use for fuelwood and fodder | moderate | Loss of land, and accessibility | moderate | Widespread (190 km²) | Local villagers have expressed concern over its invasiveness | 2 | moderate | Djoudi et al. 2011 |
| Malawi | 1970s – mass planting in 1986 and invasive by 1996 | Central America | Live fencing, agroforestry | 100 % utilization for fuelwood, 44 % incomes and 11 % fencing and 10 % shade | high | Injury (40%) impacts access 71 % loss of grazing ground 54 %,; loss of biodiversity 20 % impacts fishing, 13 %, blocks view, 6 % impacts cultivation 6 % ; biodiversity impacts | moderate | From 1986-1998 spared 10 km however are other areas with still relatively small invasion areas | Control called for by one author – biological control being considered government aware of the problem | 1 | moderate | Chikuni et al. 2004 |
| Marquesas Islands | Early 1900s | Hawaii | ? | ? | zero | ? | zero | ? | no | 1 | just occurrence | Gallaber and Merlin, 2010 |
| Mauritania | ? | ? | ? | ? | zero | ? | zero | ? | no | 2 | just occurrence | Gritzner, 1979; Jensen and Hajej, 2001 |
| Mauritius | ? | ? | ? | ? | zero | ? | zero | ? | no | 1 | just occurrence | Gallaber and Merlin, 2010 |
| Mexico | N/a | N/a | N/a | Improves local spp richness, islands of fertility; Heavy use for almost everything in honey production , fodder, fuelwood, timber the past less now not possible reason for invasiveness | high | Decreases water supply by about 10 % p/a ; impacts biodiversity, changes climate variability, alters nutrient cycling; alters hydrology patterns; reduced forb cover which impacts local herbivores | moderate | 0.9 % cover in 1937 to 13.1 % in 2003, > 30 million ha | no | 6 | good | Almanza and Moya, 1986; López-Portillo and Montana, 1999; Palacios, 2006; Ruiz et al. 2008; Foroughbakhch et al. 2012; Nie et al. 2012 |
| Mozambique | ? | ? | ? | ? | zero | ? | zero | ? | no | 1 | just occurrence | Witt, 2013 – pers. comm. |
| Namibia | 1912 | South America | Fodder | Shade and fodder | moderate | Impacts native plant and bird diversity, an negatively impacting blacked faced impala, impacting natural ecosystem function especially rivers | good | Small populations along rivers found across the whole country | Control using herbicide in the 1980s – mechanical and chemical removal from national parks in the 1970s – constant monitoring – illegal to plant *Prosopis* – 4 biocontrol agents released – evidence of private land owner clearance- small scale control by at localised areas by government | 5 | good | Brown et al. 1985; Zimmerman, 1991; Bethune et al. 2004; Smit, 2004; Shapaka et al. 2008; Poynton, 2009; Henschel and Parr, 2010 |
| Niger | 1970s and 1980s | ? introduced accidentally prior to 1977, planting in 1977; Sudan | Dune stabilisation, accidental | Agroforestry tree | moderate | Impacts water ways, and encroach on infrastructure, thus impacting local livelihoods | moderate | 300 000 ha spread of 75 00 m² p/a | Control implemented, cutting and burning – biocontrol present but not purposefully introduced – control though utilisation also been conducted | 2 | good | FAO, 2004;Geesing et al. 2004 |
| Nigeria | ? | Probably Kenya- unintentional | ? | Made into a fermented condiment | low | Impacts fishing access | low | ? | no | 1 | low | Borokini and Babalola, 2012 |
| Oman | 1970s | Dune stabilisation, local greening | Improving degraded soils | Popular ornamental species, honey source | low | Impacts native species richness and water supply | moderate | Widespread in small populations | Mechanical and chemical control program running since 1998 - poor control so far – also private clearing | 5 | moderate | Brown 1991; Ghazanfar, 1996; Al Rawahy et al. 2003;; Al Abri et al. 2004; Mahgoub et al. 2005 |
| Pakistan | Introduced 1857, Naturalised since the 1950s | Mexico | Sand/dune control, greening along roads | Fodder, fuelwood; Medicinal uses to treat respiratory and gastrointestinal ailments | high | Impacts natural vegetation and agriculture; impacts native community structures and encroaching a wild life sanctuary (Sandsprit) impacts on local livelihoods | high | Widespread common in about a third of the country approximately 250 000 km² | no | 7 | good | Felker, 1987; Pasiecznik et al. 2004; AFTD, 2009; Kazmi, 2009; Hussain et al. 2010; Khan et al. ,2011; Rashi et al. 2014. |
| Papua New Guinea | ? | ? | ? | ? | zero | ? | zero | ? | no | 1 | just occurrence | AFTD, 2009 |
| Paraguay | N/A | N/A | N/A | Agroforestry | high | ? | zero | ? | no | 1 | low | Pasiecznick et al. 2001 |
| Puerto Rico | ? | ? | ? | ? | zero | ? | zero | ? | no | 1 | just occurrence | Little and Wadsworth; 1964 |
| Qatar | 1950s | ? | ? | ? | zero | ? | zero | ? | no | 1 | just occurrence | Pasiecznick et al. 2001 |
| Reunion | ? | ? | ? | ? | zero | Disrupts ecosystems | low | ? | no | 1 | low | Kueffer and Lavergne, 2004 |
| Saudi Arabia | 1950s | ? | ? | Not used by people in the country | Impacting and displacing local vegetation in species rich areas – impacting conservation initiatives | Restricted invasions distribution but common in invasive areas |  |  | Proposed mechanical and chemical control – paper calls for the need for monitoring and experimental control | 4 | moderate | Pasiecznik et al. 2001; Hall et al. 2010; Harfi and Alsaeed, 2011; Khider et al. 2011;  Al Mautairi et al. 2012 |
| Senegal | 1822 and 1980s | Mexico – not certain | Recover degraded soils, prevent desertification | Soil improvement, fuelwood, fodder | moderate | ? | zero | ? | no | 2 | low | Pasiecznick et al. 2001 |
| Somalia | 1950s mass planting in 1970s and 1980s | ? | ? | ? | zero | ? | zero | ? | no | 1 | low | Pasiecznick et al. 2001 |
| South Africa | Introduced 1880s Naturalised by 1930s and promoted mass planting in the 1950s and 1960s, 1971, 1985 | Mexico, USA, Hawaii, Chile, Honduras | Fodder, fuelwood, shade | Fodder, fuelwood, medicine (company) | moderate | Impacts grazing potential, water supply, bird biodiversity, insect diversity, and natural vegetation diversity; impacts bird communities. | high | 1.8 million ha – 8 % per year potential to cover 30 million ha | Mechanical and chemical -programmes spent almost half a billion rand in the last 10 years – only clear just over 15 000 ha, 3 biological control agents | >10 | excellent | Harding, 1987; Harding and Bate, 1991; Zimmermann, 1991; Hoffmann et al. 1993; Hoffman et al. 1995; Steenkamp and Chown, 1996; Coetzer and Hoffmann, 1997; Pasiecznik et al. 2001; Dean et al. 2002; Poynton, 2009; Van den Berg, 2010; Ndhlovu et al. 2011; Zachariades et al. 2011; Kay et al. 2012; Mazibuko, 2012; van Wilgen et al. 2012; Wise et al. 2012; Dzikiti et al. 2013; Schachtschneider and February, 2013 |
| Sri Lanka | 1880, and again in the 1950s | Suspected India | Speculate to improve soil quality | ? | zero | Impacts on native fauna and flora; reduces grazing potential impacts human and animal health | moderate | Spreading rapidly, and widespread (20 km inland from original source, good understanding of where localised populations exist | no | 1 | moderate | Parera and Pasiecznik, 2005 |
| Sudan | 1917; 1946; 1965 | South Africa via Egypt | Dune stabilization; live fencing | Fodder, fuelwood | moderate | Displaces indigenous species; Higher than normal transpiration rates, therefore impacting limited water supply in the country, impacts on local communities livelihoods | good | 230 000 ha spread of approximately 500 ha p/a | Presidential decree to eradicate *Prosopis* - 1995; one biocontrol present, mechanical and chemical control doing small scale eradication programmes, jail sentences for non-compliance with removal, large scale education and awareness programmes promoting people to remove it from their lands; spent over 15 million US$ trying to clear at two priority sites but poor follow up control | 6 | excellent | El Fadl, 1997; Pasiecznick et al. 2001; Elfadel and Luukkanen, 2006; FAO, 2006; Laxén, 2007;  Hoshino et al. 2012. |
| Syria | N/A | N/A | N/A | ? | zero | ? | zero | ? | no | 1 | just occurrence | ISSU, 2005 |
| Tanzania | ? | ? | ? | ? | zero | ? | zero | ? | no | 1 | just occurrence | Witt, 2013 – pers. comm. |
| Turkey | N/A | N/A | N/A | ? | zero | ? | zero | ? | no | 1 | just occurrence | Pasiecznick et al. 2001 |
| Tunisia | ? | ? | ? | ? | zero | ? | zero | ? | no | 1 | just occurrence | Pasiecznick et al. 2001 |
| Turkmenistan | N/A | N/A | N/A | ? | zero | ? | zero | ? | no | 1 | just occurrence | GBIF, 2013 |
| Ukraine | N/A | N/A | N/A | ? | zero | ? | zero | ? | no | 1 | just occurrence | Pasiecznik et al. 2001 |
| United Arab Emirates | 1970s | ? | For greening of landscapes and desertification control and afforestation | ? | zero | Impacts native species; impacts soils; encroaches natural and managed habitats causing degradation and land abandonment; impacts species richness in conservation areas | moderate | Spreads on average 1.2 % per patch size increase p/a over 100 stands - (each stand approximately 225 m²) widespread distributed across various sites – density of 10-35 individuals per 100m² | no | 6 | good | El-Keblawy and Al-Rawai, 2005; El-Keblawy and Ksiksi 2005;El-Keblawy and Al-Rawai, 2007;Issa and Dohai, 2008; AFTD, 2009; El-Keblawy, 2012 |
| United states of America | N/A | N/A | N/A | Increases native species richness; benefits denning of skunks, fuelwood | low | Impacts ecosystem services, hydrology, livelihoods, biodiversity, decreased system resilience, impacts grazing potential; altered vegetation communities ,losses to livestock industry 200-500 million US$ p/a, soil erosion; water impacts- decreased supply by about 10 %; impacts soils, impacts natural fire regimes; shifts in rodent populations; outcompetes other native species; alters soil altered soil bacterial and fungal communities; displaces riverine trees and grasses on plains; facilitates other exotics; loss of wetlands; decline in cactus communities | high | Cover of *Prosopi*s doubled from 1936 to 1996 increased by 80 000 ha between 1973-1997 in a catchment in Arizona small study site in Texas – cover of between 81-150 km, 38 million ha in south-western USA | Over 50 years of management, Fire; stocking rates – lots of experimental eradication sites, mechanical and chemical control, repeated burning by farmers and conservation officers – burning not very efficient | >10 | excellent | Johnston, 1962; DeLoach, 1984; Ethridge et al. 1984; Torell and McDaniel, 1986; Archer 1989; Brown and Archer 1989; Dugas and Mayeux, 1991; Bahre and Shelton, 1993; Stromberg et al. 1993; Polley et al. 1994; Warren et al. 1996; Nolte and Fulbright, 1997; Lloyd et al. 1998; Teague et al. 2001; Goslee et al. 2003; Ansley et al. 2004; Bestelmeyer 2005; Grandtner, 2005; Kreuter et al. 2005; McClaran and Angell, 2006; Bock et al. 2007; Browning et al. 2008; Teague et al. 2008; Throop and Archer, 2008; Eggemeyer and Schwinning, 2009; Ansley et al. 2010; Havstad and James, 2010; Hollister et al. 2010; de Dios et al. 2012; Nie et al. 2012; Creamer et al. 2013 |
| Venezuela | N/A | N/A | N/A | ? | zero | ? | zero | ? | no | 1 | low | Pasiecznick et al. 2001 |
| Yemen | Accidental pre1950s; Purposeful 1974 | Probably Sudan or Somalia | Accidental initially – but later promoted for dune stabilisation and fuelwood, erosion control and greening | Fuelwood | moderate | Impacts agriculture buy encroaching land, and is clogging up canals | moderate | 350-400 thousand ha | Costs on average 4.5 US$ per ha to clear using bulldozer and fire (mechanical clearing in canals (2 million US$ p/a), one biocontrol but was not purposefully released, promoting utilisation as control approach with support from FAO | 3 | good | Felker, 2003; Geesing et al. 2004; FAO, 2006 |

Admasu D. 2008. Invasive plants and food security: the case of *Prosopis juliflora* in the Afar region of Ethiopia. Ethiopia: FARM-Africa and IUCN.

Agroforestree Database (AFTD). 2009. Prosopis. Kenya: World Agroforestry Centre.

Al Abri AS, Al Ajmi DS, Al Halhali AS, Al Saqry NM, Forsberg NE, Kadim IT, Mahgoub O, Richie AR. 2004. In: Salem B, Nefzaoui A, Morand-Fehr P, eds. Nutrition and feeding strategies of sheep and goats under harsh climates. Zaragoza: CIHEAM.

Almanza SG, Moya EG. 1986. The uses of mesquite (*Prosopis* spp.) in the highlands of San Luis Potosi, Mexico. *Forest Ecology and Management* 16:49-56.

Al Mutairi K, El-Bana M, Mansor M, Al-Rowaily S, Mansor A. 2012. Floristic diversity, composition and environmental correlates on the arid Coralline Islands of the Farasan Archipelago, Red Sea, Saudi Arabia. *Arid Land Reserch and Management* 26:137-150.

Al Rawahy SH, Al Dhafri K, Al Bahlani SS. 2003. Germination, growth and drought resistance of native and alien plant species of the genus Prosopis in the Sultanate of Oman. Asian Journal of Plant Sciences 2:1020-1023.

Anderson S. 2005. *Spread of the introduced tree species Prosopis juliflora (Sw.) D.C*. Sveriges lantbruksuniversitet: Institutionen för skoglig vegetationsekologi.

Anderson LJ, van Klinken RD, Parr RJ, Climas R, Barton D. 2006. Integrated management of hybrid mesquite: a collaborative fight against one of Australia’s worst woody weeds. Adelaide: *Fifteenth Australian Weeds Conference*.

Ansley RJ, Pinchak WE, Teague WR, Kramp BA, Jones DL, Jacoby PW. 2004. Long-term grass yield following chemical control of honey mesquite. *Journal of Range Management* 57:49-57.

Ansley RJ, Boutton TW, Mirik M, Castellano MJ, Kramp BA. 2010. Restoration of C4 grasses with seasonal fires in a C3/C4 grassland invaded by *Prosopis glandulosa*, a fire-resistant shrub. *Applied Vegetation Science* 13:520-530.

Archer S. 1989. Have southern Texas savannas been converted into woodlands in recent history? *American Naturalist*  134:545-561.

Bahre CJ, Shelton ML.1993. Historic vegetation change, mesquite increases, and climate in southeastern Arizona. *Journal of Biogeography* 20:489-504.

Belton T. 2008*. Management Strategy for Mexican thorn (Prosopis juliflora) on Ascension Island: An assessment of this species, and recommendations for management*. Bedfordshire: RSPB.

Berhanu A, Tesfaye G. 2006. The *Prosopis* dilemma, impacts on dryland biodiversity and some controlling methods. *Journal of the Drylands* 1L158-164.

Bestelmeyer BT. 2005. Does desertification diminish biodiversity? Enhancement of ant diversity by shrub invasions in south-western USA. *Diversity and Distributions* 11:45-55.

Bethune S, Griffin M, Joubert D. 2004. National review of invasive alien species Namibia. Windhoek. Directorate of Environmental Affairs, Ministry of Environment and Tourism.

Bhatt SS, Chovatiya SG, Shah AR. 2011. Evaluation of raw and hydrothermically processed *Prosopis juliflora* seed meal as supplementary feed for the growth of *Labeo rohita* fingerlings. *Aquaculture Nutrition* 17:164-173.

Bock CE, Kennedy L, Bock JH, Jones ZF. 2007. Effects of fire frequency and intensity on velvet mesquite in an Arizona grassland. *Rangeland Ecology and Management* 60:508-514.

Bogino SM, Villalba R. 2008. Radical growth and biological rotation age of *Prosopis caldenia* Burkart in Central Argentina. *Journal of Arid Environments* 72:16-23.

Bokrezion H. 2008. The ecological and socio-economic role of *Prosopis juliflora* in Eritrea: An analytical assessment within the context of rural development in the Horn of Africa. PhD Thesis, Johannes Gutenberg University, Mainz.

Borokini TI, Babalola FD. 2012. Management of invasive plant species in Nigeria through economic exploitation: lessons from other countries. *Management of Biological Invasions* 3:45-55.

Botswana Government, 2009. Botswana fourth national report to the convention of biological diversity. Gaborone: Botswana Government.

Brown CJ, Macdonald IAW, Brown SE. 1985. Invasive alien organisms in South West Africa/Namibia. South Africa. Foundation for Research Development.

Brown JR, Archer S. 1989. Woody plant invasion of grasslands: establishment of honey mesquite (*Prosopis gnaldulosa* var. *glandulosa*) on sites differing in herbaceous biomass and grazing history. *Oecologia* 80:19-26.

Brown K. 1991. Biology of *Prosopis cineraria* (Leguminosae) in the Sultanate of Oman. Phd in Science. Durham: Durham University.

Browning DM, Archer S, Asner G, McClaran MP, Wessman CA. 2008. Woody plants in grasslands post-encroachment and stand dynamics. *Ecological applications* 18:928-944.

Busch M, Knight C, Hodara CNMK, Chaneton EME. 2012. Rodent seed predation on tree invader species in grassland habitats of the inland Pampa. *Ecological Research* 27:369-376.

Cardoso MB, Ladio AH, Lozada M. 2012. The use of firewood in a Mapuche community in a semi-arid region of Patagonia, Argentina. *Biomass and Bioenergy* 46;155-164.

Chandrasekaran S Swamy PS. Undated. Ecological and socio-economic impacts of *Prosopis juliflora* invasion into the semi-arid ecosystems in selected villages of Ramand district in Tamilnadu. Madurai: Madurai Kamaraj University.

Chikuni MF, Dudley CO, Sambo EY. 2004. Prosopis glandulosa Torry (Leguminosae-Mimosoidae) at Swang’oma, Lake Chilwa plain: A blessing in disguise. Malawi Journal of Science and Technology 7:10-16.

Choge SK, Ngujiri FD, Kuria MN, Busaka EA, Muthondeki JK. 2002. The status and impact of Prosopis spp. in Kenya. Nairobi: KEFRI.

Choge SK, *Chikami* BN. 2004. Experiences of Prosopis utilization and management from outside Kenya. Proceedings of the Workshop on Integrated Management of Prosopis Species in Kenya. Nairobi, Kenya: KEFRI.

Choge SK, Harvey M, Chesang S, Pasiecznik NM. 2006. Cooking with Prosopis flour. Recipes tried and tested in Bango District, Kenya. Nairobi and Coventry: KEFRI and HDRA.

Choge SK, Pasiecznik NM, Harvey M, Wright J, Awan SZ, Harris PJC. 2007. *Prosopis* pods as human food, with special reference to Kenya. *Water South Africa* 33:419-424.

Choge SK, Clement N, Gitonga M, Okuye J. 2012. Status report on commercialization of Prosopis tree resources in Kenya, Technical report for the KEFRI/KFS Technical Forest Management and Research Liaison Committee. Nairobi: KEFRI.

Cibichakravarthy B, Preetha R, Sundaram SP, Kumar K, Balachandar D. 2012. Diazothrophic diversity in the rhizosphere of two exotic weed plants, *Prosopis juliflora* and *Parthenium hysterophorus. World Journal of Microbiology and Biotechnology* 28;605-613.

Cienciala E, Centeio A, Balazek P, da Cruz Gomes Soares M, Russ R. 2013. Estimation of stem and tree level biomass models for Prosopi juliflora/pallida applicable to multi-stemmed tree species. Trees 27:1061-1070.

Coetzer W, Hoffmann JH. 1997. Establishment of *Neltumius arizonensis* (Coleoptera: Bruchidae) on Mesquite (*Prosopis* species: Mimosaceae) in South Africa. *Biological Control* 10:187-182.

Creamer CA, Filley TR, Olk DC, Stott DE, Dooling V, Boutton TW. 2013. Changes to soil organic N dynamics with leguminous woody plant encroachment into grasslands. *Biogeochemistry* 113:307-321.

Csurhes S. ed. 1996. *Mesquite (Prosopis spp) in Queensland*. Australia: Department of Natural Resources and Mines.

Dayal V. 2007. Social diversity and ecological complexity: how an invasive tree could affect diverse agents in the land of the tiger. *Environment and Development Eonomics* 12:553-571.

Dean WRJ, Anderson MD, Milton SJ, Anderson TA. 2002. Avian assemblages in native Acacia and alien Prosopis drainage line woodland in the Kalahari, South Africa. Journal of Arid Environments 51:1-19.

De Andrade AA, Fabricante JR, deOliveira FX. 2010. Impactos da invasao de *Prosopis juliflora* (sw.) DC. (Fabaceae) sobre o estrato arbustivo-arboreo em areas de Caatinga no Estafo da Paraiba, Braazil. *Maringá* 32:249-255.

de Dios V, Weltzin JF, Huxman TE, Williams DG. 2012. Windows of opportunity for *Prosopis velutina* seeding establishment and encroachment in a semiarid grassland. *Perspectives in Plant Ecology, Evolution and Systematics* 14:275-282.

DeLoach CJ. 1984. Conflicts of interest over beneficial and undesirable aspects of Prosopis (Prosopis spp.) in the United States as related to biological control. Vancouver, Canada: 6th International Symposium on Biological Control, 19–25 August 1984, pp. 301–340.

Demissie H. 2009. Invasion of *Prosopis juliflora* (Sw.) DC. Into Awash National Park and its impacts on plant species diversity and soil characteristics. Masters in Science. Addis Ababa: Addis Ababa University.

de Oliveira LSB, de Andrade LA, Fabricante JR, Gonçalves GS. 2012. Structure of a Prosopis juliflora (Sw.) DC. Population established in a temporary riverbed in the microregion of Cariri in the Stat of Paraiba. Semina: Ciências Agrárias, Londrina 33(5):1769-1778.

de Villalobos AE, Paláez DV, Elia OR. 2005a. Factors related to establishment of *Prosopis caldenia* Burk. Seedlings in central rangelands of Argentina. *Acta Oecologica* 27:99-106.

de Villalobos AE, Paláez DV, Elia OR. 2005b; Growth of *Prosopis cladenia* Burk: seedlings in central semi-arid rangelands of Argentina. *Journal of Arid Environments* 61:345-346.

de Villalobos AE, Peláez DV, Bóo RM, Mayor MD, Elia OR. 2007. Effect of a postfire environment on the establishment of *Prosopis caldenia* seedlings in central semiarid Argentina. *Austral Ecology* 32:581-591.

Distel RA, Peláez DV, Bóo RM, Mayor MD, Elia OR. 1996. Growth of *Prosopis caldenia* seedlings in the fields as related to grazing history of the site and in a greenhouse as related to different levels of competition from *Stipa tenuis*. *Journal of Arid Environments* 32:251-257.

Djoudi H, Brockhaus M, Locatelli B. 2011. Once there was a lake: vulnerability to environmental changes in northern Mali. Regional Environmental Change DOI 10.1007/s10113-011-0262-5.

Dufgas WA, Mayeux HS. 1991. Evaporation from rangeland with and without honey mesquite. *Journal of range Management* 44:161-170.

Dussart E, Peinetti R, Boninesegra JA. 1997. Análisis del crecimiento de *Prosopis caldenia* (L) Burk. En relación con parametros ambientales y fuego. Buenos Aires: Reunión Argentina de Ecologia.

Dzikiti S, Schachtschneider K, Naiken V, Gush M, Moses G, Le Maitre DC. 2013. Water relations and the effects of clearing invasive *Prosopis* trees on groundwater in an arid environment in the Northern Cape, South Africa. *Journal of Arid Environments* 90:103-113.

Eggemeyer KD, Schwinning S. 2009. Biogeography of woody encroachment: why is mesquite excluded from shallow soils? *Ecohydrology* 2:81-87.

Elfadl MA. 1997. Management of Prosopis juliflora for use in agroforestry systems in the Sudan. Helsinki: Tropical Forestry Reports 16, University of Helsinki.

Elfadl MA, Luukkanen O. 2006. Field studies on the ecological strategies of *Prosopis juliflora* in a dryland ecosystem 1. A leaf gas exchange approach. *Journal of Arid Environments 66:1-15.*

El-Keblawy A, Al-Rawai A. 2005. Effect of salinity, temperature and light on germination of invasive *Prosopis juliflor* (Sw.) D.C. *Journal of Arid Environments* 61:555-565.

Al-Keblawy. 2012. *Impacts of native and exotic Prosopis species on native plants in aridlands of the UAE*. Phuket: International Conference on Ecology, Agriculture and Chemical Engineering December 18-19.

El-Keblawy A, Ksiksi T. 2005. Artificial forests as conservation sites for the native flora of the UAE. *Forest Ecology and Management* 213:288-296.

El-Keblawy A, Al-Rawai A. 2007. Impacts of the invasive exotic *Prosopis juliflora* (Sw.) D.C. on the native flora and soils of the UAE.

Emlan JT. Land-bird densities in matched habitats on six Hawaiian Islands: A test of resource regulation theory. *The American Naturalist* 127:125-139.

Ethiopia Government. 2002. Ethiopia-National Plan for *Prosopis* – Final Draft. Ethiopia Government.

Ethridge DE, Dahl BE, Sosebee RE. 1984. Economic evaluation of chemical mesquite control using 2,4,5-T. *Journal of Range Management* 37:152-156.

FAO. 2004. The introduction of *Prosopis* spp. In the drylands of the world ledge of plight for biodiversity. Food and Agricultural Organization of the United Nations

FAO. 2006. Problems posed by the introduction of Prosopis spp. in selected countries. Rome: Plant Production and Protection Division, Food and Agricultural Organization of the United Nations.

FARM- Africa. 2008. *Experiences on Prosopis management case of Afar Region.* Ethiopia: FARM-Africa.

Felker P. 1987. Recommendations for development of *Prosopis* in Pakistan. Texas: Centre for Semi-Arid Forest Resources.

Felker P, 2003. Management, use and control of *Prosopis* in Yemen. Yemen: Project Number: TCP/YEM/0169(A).

Foroughbakhch R, Parra AC, Pinero JLH, Vázquez MAA, Estrada AR, Cardenas ML. 2012. Wood volume production ans use of 10 woody species in semiarid zones of northeastern Mexico. *International Journal of Forestry Research doi:10.1155/2012/529829*

Gallaber T, Merlin M. 2010. Biology and impacts of Pacific Island invasive species. 6. Prosopis pallida and Prosopis juliflora (Algarroba, Mesquite, Kiawe) (Fabaceae). Pacific Science 64: 489-526.

Geesing D, Al-Khawlani M, Abba ML. 2004. Management of introduced Prosopis species: Can economic exploitation control and invasive species? Unasylva 217:36-44.

Ghazanfar SA. 1996. Invasive Prosopis in the Sultanate of Oman. Alien 3:10.

Ghazaly UF. 2006. Community-based management of invasive Prosopis juliflora in Egypt. Washington: GISP.

Global Biodiversity Information Facility (GBIF). 2013. Prosopis species Lists. Copenhagen and Denmark: GBIF.

Goslee SC, Havstad KM, Peters DP, Rango A, Schlesinger WH. 2003. High-resolution images reveal rate and pattern of shrub encroachment over six decades in New Mexico, U.S.A. *Journal of Arid Environments* 54:755-767.

Grandtner MM. 2005. ELSEVIER’S Directory of trees. Amsterdam: ELSEVIER.

Gritzner JA. 1979. Environmental degradation in Mauritania: Staff Report. Mauritania: Board on Science and Technology for International Development, Commission on International Relations and National research Council.

Hall M, Llewellyn OA, Miller AG, Al-Abbasi TM, Al-Wetaid AH, Al-Harbi RJ, Al-Shammari KF. 2010. Important plant areas in the Arabian Peninsula: 2. Farasan Archipelago. Edinburgh Journal of Botany 67(2):189-208.

Harding GB. 1987. *The status of Prosopis as a weed*. Uitenhage: South Africa: Plant Protection Research Institute.

Harding GB, Bate GC. 1991. The occurrence of invasive Prosopis species in the north- western cape, South Africa. South African Journal of Science, 87: 188-192.

Harfi HH, Alsaeed AH. 2011. Allergenicity to allergins like *Prosopis juliflora* and date tree pollens in Saudi Arabia. *Kuwait Medical Journal* 43:109-112.

Hasan MK, Alam AKAA, 2006. Land degradation situation in Bangladesh and the role of Agroforestry. *Journal of Agriculture and Rural Development* 4:19-25.

Havstad KM, James D. 2010. Prescribed burning to affect a state transition in a shrub-encroached desert grassland. *Journal of Arid Environments* 74:1324-1328.

Hawaii Ecosystem at Risk Project. 1998. *Prosopis pallida* (Fabaceae). Map of estimated distribution on Molokai. Hawaii: Hawaii Ecosystem at Risk Project

Henschel JR, Parr T. 2010. Population changes of alien invasive plants in the Lower Kuiseb River. Windhoek, Namibia: DINTERIA No. 31: 5-17.

Hoffman, M.T. Sonnenberg, D., Hurford, J.L and Jagger, B.W. 1995. Ecology and management of Riemvasmaak’s natural resources. National Botanical Institute, Cape Town.

Hoffmann JH, Impson FAC, Moran VC. 1993. Competitive interactions between two Burchid species (*Algarobius* spp.) introduced into South Africa for biological control of mesquite weeds 9*Prosopis* spp.). *Biological Control* 3:215-220.

Hollister EB, Schadt CW, Palumbo AV, Ansley JR, Boutton TW. 2010. Structural and functional diversity of soil bacteria and fungal communities following woody plant encroachment in the southern Great Planes. *Soil Biology and Biochemistry* 42:1816-1824.

Hoshino B, Karamalla A, Abd Elbasit MAM, Manayeva K, Yoda K, Suliman M, Elgamri M, Nawata H, Yasuda H. 2012. Evaluating the invasion strategic of Mesquite (*Prosopis juliflora*) in eastern Sudan using remotely sensed technique. *Journal of Arid Land Studies* 22:1-4.

Hussain SS, Ahmed M, Siddiqui MF, Wahab M. 2010. Threatened and endangered native plants of Karachi. International Journal of Biology and Biotechnology 7(3):259-266.

Ibrahim F. 2004. No place like home: History, politics and mobility among a pastoral nomadic community in western India. *Nomadic Peoples* 8:168-190.

Issa S, Dohai B. 2008. GIS analysis of invasive *Prosopis juliflora* dynamics in two selected sites from the United Arab Emirates. *Canadian Journal of Pure and Applied Sciences* 2:235-242.

Jensen AM, Hajej MS. 2001. The road of hope: control of moving sand dunes in Mauritania. Unasylva 207:31-36.

Jhala YV. 1993. Predation on blackbuck by wolves in the Velavadar National Park, Gujarat, India. *Conservation Biology* 7:874-881.

Johnston MC. 1962. The North American mesquites Prosopis Sect. Algarobia (Leguminosae). Brittonia 14(1):72-89.

Joshi EB, Joshi PN, Jain BK. 2012. Phytosociological study and management action for the invasive weed : *Prosopis juliflora* (SW.) DC. in Tapkeshwari Hill Ranges in the Kachchh Island, Gujarat, India. *Journal of Non-Timber Forest Products* 19:29-36.

Kahi CH, Ngugi RK, Mureithi SM, Ng’ethe JC.2009. The canopy effects of *Prosopis juliflora* (DC.) and *Acacia tortillis* (hayne) trees on herbaceous plants species and soil physio-chemical properties in Njemps Flats, Kenya. *Tropical and Subtropical Agroecosystems* 10:441-449.

Kazmi JH. 2009. Ecological and Socio-economic evaluation of the use of *Prosopis juliflora* for bio-char production in Pakistan. Pakistan: Drynet

Khan I, Marwat KB, Khan IA, Ali H, Dawar K, Khan H. 2011. Invasive weeds of southern districts of Khyber Pakhtunkhwa –Pakistan. Pakistani Journal of Weed Science Research 17(2):161-174.

Kaur R, Gonzales WL, Llambi LD, Soriano PJ, Callaway RM, Rout ME, Gallaher JT, Inderjit. 2012. Community impacts of Prosopis juliflora invasion: Biogeographic and congeneric comparisons. PLoS One 7:e44966.

Khider MM, Al Abjar ZA, Eltigani A. 2011. Studies on ecology and biology of some insect pests of the mesquite trees *Prosopis juliflora* in Sudan. *Egyptian Journal of Biological Pest Control* 21:353-359.

Kreuter UP, Amestoy HE, Kothmann MM, Ueckert DN, McGinty WA, Cummings SR. 2005. The use of brush management methods: A Texas landowner survey. *Rangeland Ecology and Management*  58:284-291.

Kueffer C, Lavergne C. 2004. Case studies on the status of invasive woody plant species in the western Indian ocean. Rome: FAO.

Lasalle JC. 1962. El increment de la masa forestall del caldén (*Prosopis caldenia* Burk). *Revista Forestal Argentina* 6:44-50.

Lauenstein DAL, Fernanández ME, Verga AR. 2013. Drought stress tolerance of *Prosopis chilensis* and *Prosopis flexuosa* species and their hybrids. *Trees 27:285-296.*

Laxén J. Is prosopis a curse or a blessing? – An ecological – economic analysis of an invasive alien tree species in Sudan. Dissertation. Helsinki: University of Helsinki.

Leão, TCC, de Almeida WR, de Sá Dechoum M, Ziller SR. 2011. Espécies exόticas invasoras: no Nordeste do Brasil. Brazil: CEPAN Instituto Hόrus.

Lee SG, Russel EJ, Bingham RL, Felker P. 1992. Discovery of thornless, non-browsed, erect tropical Prosopis in 3-year-old Hatian progeny trials. Forest Ecology and Management 48:1-13.

Lemlem N. 2003. *Mapping of Prosopis juliflora using remote sensing and GIS – Case srudy of Marigat Division, Baringo District*. Bachelor of Science in Surveying. Nairobi: University of Nairobi.

Little EL, Wadsworth FH. 1964. Common trees of Porto Rico and the Virgin Islands. Washington, D.C: Department of Agriculture, Forest Services

Lloyd J, Mannan RW, Destefano S, Kirkpatrick C. 1998. The effects of mesquite invasion on a southeastern Arizona grassland bird community. *Wilson Bulletin* 110:403-408.

López-Portillo J, Montana C. 1999. Spatiall distribution of *Prosopis glandulosa* var. *torreyana* in vegetation strips of the southern Chihuahuan Desert. *Acta Oecologica* 20:197-208.

Lynes BC, Campell SD. 2000. Germination and viability of mesquite (*Prosopis pallida*) seed following ingestion and excretion by feral pigs (*Sus scrofa*). *Tropical Grasslands* 34:125-128.

Mahgoub O, Kadim IT, Johnston EH, Srikandakumar A, Al-Saqri NM, Al-Abri AS, Ritchie A. 2005. The use of concentrate containing Meskit (*Prosopis juliflora*) pods and date palm by-products to replace commercial concentrate in diets of Omani sheep. *Animal Feed Scinace and Technology* 120:33-41.

Maundu P, Kibet S, Morimoto Y, Imbumi M, Adeka R. Impact of *Prosopis juliflora* on Kenya’s semi-arid and arid ecosystems and local livelihoods. *Biodiversity* 10:33-50.

Mazibuko DM. 2012. *Phylogenetic relationships of Prosopis in South Africa: An assessment of the extent of hybridization, and the role of genome size and seed size in the invasion dynamics.* MSc Thesis, Stellenbosch University, Stellenbosch.

McClaran MP, Angell DL. Long-term vegetation response to mesquite removal in Desert Grassland. *Journal of Arid Environments* 66:686-697.

Mc Kay F, Gandolfo D, Witt ABR. 2012. Biology and host range of *Coelocephalapion gandolfoi* Kissinger (Brentidae), a promosing candidate for the biological control of invasive *Prosopis* species (Legumiosae) in South Africa. *African Entomology* 20:281-291.

Medina AA, Dussart EG, Estelrich HD, Morici EA. 2000. Reconstrucción de la historia del fuego en un bosque de *Prosopis caldenia* (Burk.) de Arizona, south of San Luis Province *Multequina* 9:91-98.

Miller C, Darlow A. 2008. A reiview of the Ascension Island action plan for SAIS Project. RSPB.

Miranda RQ, Oliveira MTP, Correia RM, Almedia-Cortez JS, Pompelli MF. 2011. Germination of *Prosopis juliflora*(Sw) DC seeds after scarification treatments. *Plant Species Biology* 26:186-192.

Muturi GM, Mohren GMJ, Kimani JN. 2009. Prediction of *Prosopis* species invasion in Kenya using geographical information system techniques.

Muturi GM, Poorter L, Mohren GMJ, Kigomo BN. 2013. Ecological impact of *Prosopis* species invasion in Turkwel riverine forest, Kenya. *Journal of Arid Environments* 92:89-97.

Muzila M, Setshogo MP, Moskei B, Morapedi R. 2011. An assessment of *Prosopis* L. in the Bokspits area south-western Botswana, based on morphology. *The African Jounral of Plant Science and Biotechnology*  5:75-80.

Mwangi M, Swallow B. 2005. Invasion of Prosopis juliflora and local livelihoods: Case study from the Lake Baringo area of Kenya. ICRAF Working Paper – no. 3. Nairobi: World Agroforestry Centre.

Mworia JK, Kinyamario JL, Omari JK, Wambua JK. 2011. Patterns of seed dispersal and establishment of invader *Prosopis juliflora* in the upper floodplain of Tana River, Kenya. *African Journal of Range and Forage Science* 28:35-41.

Naseeruddin S, Yadav KS, Sateesh L, Manikyam A, Desai S, Rao LV. 2013; Selection of the best chemical pretreatment for lignocellulosic substance *Prosopis juliflora*. *Bioresource Technology* 136:542-549.

National Weeds Strategy Executive Committee. 2001. *Mesquite (Prosopis Speceis) Strategic Plan*. Launceston: National Weeds Strategy Executive Committee.

Ndhlovu T, Milton-Dean SJ, Esler KJ. 2011. Impact of Prosopis (mesquite) invasion and clearing on the grazing capacity of semiarid Nama Karoo rangeland, South Africa. African Journal of Range and Forage Science 28:129-137.

Nie W, Yuan Y, Kepner W, Erickson C, Jackson M. 2012. Hydrological impacts of mesquite encroachment in the upper San Pedro watershed. Journal of Arid Environments 82:147-155.

Njoroge E, Sirmah P, Mburu F, Koech E, Mware M, Chepkwony J. 2012. Preference and adoption of farmer field school (FFS) *Prosopis juliflora* management practices: Experiences in Baringo District, Kenya. Forestry Studies in China 14(4):283-290.

Nolte KR, Fulbright TE. 1997. Plant, small mammal and avian diversity following control of honey mesquite. *Journal of Range Management*  50:205-212.

Northern Territory Government. 2012. Weed management plan for mesquite (Prosopis species). Palmeton: Northern Territory Government.

Osmond R. 2003. *Best practice manual, mesquite: Control and management options for mesquite (Prosopis spp.) in Australia*. Queensland: National Weeds Programme and Queensland Department of Natural Resources and Mines.

Page AR, Lacey KL. 2006. *Economic impact assessment of Australia weed biological control. CRC for Australian Weed Management*. Australia.

Palacios RA. 2006. Los mezquites Mexicanos: Biodiversidad y distrabucion geografica. *Bulletin of the Botanical Society of Argentina* 41:99-121.

Pasiecznik NM, Felker P, Harris PJC, Harsh LN, Cruz G, Tewari JC, Cadoret K, Maldonado LJ. 2001. *The Prosopis juliflora-Prosopis pallida complex: A monograph*. Coventry, UK: HDRA.

Pasiecznik NM, Harris PJC, Smith SJ. 2004. Identifying tropical Prosopis species: A field guide. Coventry, UK: HDRA.

Pasiecznik NM, Choge SK, Muthike GM, Chesang S, Fehr C, Bakewell-Stone P, Wright J, Harris PJC. 2006. Putting Knowledge on Prosopis into Use in Kenya. Pioneering Advances in 2006. Nairobi and Coventry, UK: KEFRI and HDRA.

Pandey CN, Pandey R, Bhatt JR. 2012. iProsopis juliflora (*Swartz*) D.C.: Management dilemmas and regulatory issues in Gujarat. In *Invasive alien plants: An ecological appraisal for the Indian subcontinent* Bhatt (et al (eds). CAB International.

Panetta FD, Carstairs SA. 1989. Isozymic discrimination of tropical Australian populations of mesquite (*Prosopis* spp.): implications for biological control. *Weed Research* 29:157-165.

Peinetti R, Sosa A, Kin A, Cerqueira E. 1997. Modelo de simulación del banco de semillas del caldén (*Prosopis caldenia*) *Reunión Argentina de Ecologia* 48:98.

Perera ANF, Pasiecznik NM. 2005. Using invasive Prosopis to improve livelihoods in Sri Lanka. Coventry UK: HDRA.

Pickup AR. 1999. *Ascension Island Management Plan*. Report from RSPB and Birdlife International.

Polley HW, Johnson HB, Mayeux JH. 1994. Increasing CO2: Comparitive responces of the C4 grass schizachyrium and grassland invader Prosopis. *Ecology* 75:976-988.

Poynton RJ. 2009. *Tree planting in southern Africa, volume 3: Other genera*. Pretoria, South Africa: Department of Agriculture, Forestry and Fisheries.

Qasem JR. 2007. Chemical control of Prosopis farcta (Banks and Sol.) Macbride in the Jordan Valley. Crop Protection 26:572-575.

Ræbild A, Diallo BO, Graudal L, Dao L, Sanou J. 2003.Evaluation of a species and provenance trial of Prosopis at Gonsé, Nurkina Faso. Rome: FAO

Rashid M, Abbas SH, Rehman A. 2014. The status of highly alien invasive plants in Pakistan and their impact on the ecosystem: A review. *Innovare Journal of Agricultural Science* 2:1-4.

Reddy CS, Rangaswamy M, Pattanaik C, Jah CS. 2009. Invasion of alien species in wetland of Samaspur Bird Sanctuary, Uttar Pradesh, India. *Asian Journal of water, Environment and Pollution* 6:43-50.

Robinson TP, van Klinken RD, Metternicht G. 2008. Spatial and temporal rates and patterns of mesquite (*Prosopis* species) invasions in Western Australia. *Journal of Arid Environments* 72:175-188.

Ruiz TG, Zaragoza SR, Cerrato RF. 2008. Fertility islands around *Prosopis laevigata* and *pachycereus hollianus*  in the drylands of Zapotitlán Salinnas, Mexico. *Journal of Arid environments* 72:1202-1212.

Sato T. 2013. Beyond water-intensive agriculture: Expansion of *Prosopis juliflora* and its growing economic use in Tamil Nadu, India. *Land Use Policy* 35:283-292.

Sajad G, Sefidi K. 2012. Comparison of sustainable forest management (SFM) trends at global and country levels: case study in Iran. Journal of forestry Research 23(2):311-317.

Schachtschneider K, February E. Impact of *Prosopis* invasion on a keystone tree species in the Kalahari Desert. *Plant Ecology* 214:597-605.

Seid MJ. 2012. Household perception about *Prosopis juliflora* and its effect on pastoral livelihood diversification strategy: The case of Gewane District in Afar Regional State, Ethiopia. *International Journal of Agricultural Science and Research* 2:21-51.

Shapaka TN, Cunningham PL, Joubert DF. 2008. Invasive alien plants in the Daan Viljoen Game Park. Windhoek: DINTERIA 30:19-32.

Sharma R, Dakshini KMM. 1998. Intergration of plant and soil charecteristics and the ecological success of two *Prosopis* species. *Plant Ecology* 139:63-69.

Shiferaw H, Teketay D, Nemomissa S, Assefa F. 2004.Some biological characteristics that foster the invasion of Prosopis juliflora (Sw.) DC.at Middle Awash Rift Valley Area, north-eastern Ethiopia. Journal of Arid Environments 58:135-154.

Sigh A, Singh PK. 2009. An ethnobotanical study of medicinal plants in Chandauli District of Uttar Pradesh, India. *Journal of ethnopharmacology* 121:324-329.

Singh G, Shukla S. 2012. Effect of *Prosopis julifora* (DC.) tree on under canopy resources, dicersity and productivity of herbaceous vegetation in Indian desert. *Arid Land Research and Management* 26:151-165.

Skolmen RG. Undated. *Prosopis pallida* (Humb. And Bonpl. Ex Willd.) H.B.K. Kiawe.

Smit P. 2004. Prosopis: a review of existing knowledge relevant to Namibia. Journal of the Scientific Society 52:13-40.

Sohrabi S, Gherekhloo J, Mohassel MHR, Ghanbari A, Mahalati MN. 2011. *Cardinal temperatures of three invasive weeds in Iran*. Acuna, Switzerland: 3^rd^ International Symposium on Weeds and Invasive Plants.

Steele P, Breithaupt J, Labrada R. 2008. Proceedings Expert Consultation (no. 4): Increases food security control and management of *Prosopis.* Ethiopia. FAO.

Steenkamp HE, Chown SL.1996. Influence of dense stands of an exotic tree Prosopis glandulosa Benson, on a savanna dung beetle (Coleoptera: Scarabeidae) assemblage in southern Africa. Biological Conservation 78:305–311.

Stromberg JC, Wilkins SD, Tress JA. 1993. Vegetation-hydrology models: Implications for management of *Prosopis velutina* (velvet mesquite) riparian ecosystems. *Ecological Applications* 2:307-314.

Tabosa IM, Riet-Correa F, Barros SS, Summers BA, Simoes SVD, Medeiros RMT, Nobre VMT. 2006. Neurohistologic and ultrastructural lesions in cattle experimentaly intoxicated with the plant *Prosopis juliflora. Veterinary Pathology* 43:695-701.

Teague WR, Ansley RJ, Kreuter UP, Pinchak WE, McGrann JM. Economics of managing mesquite in northern Texas: A sensitivity analysis. *Journal of Range Management* 54:553-560.

Teague WR, Grant WE, Kreuter UP, Diaz-Solis H, Dube S, Kothmann MM, Pinchak WE, Ansley RJ. 2008. An ecological economic simulation model for assessing fire and grazing management effects on mesquite rangelands in Texas. *Ecological Economics* 64:611-624.

Tessema Ya. 2012. Ecological and Economic Dimensions of the paradoxical invasive species – *Prosopis juliflora* and policy changes in Ethiopia. *Journal of Economics and Sustainable Development*  3:62-71.

Throop HL, Archer SR. 2008. Srub (*Prosopis velutina*) encroachment in a semidesert grassland: special-temporal changes in soil organic carbon and nitrogen pools. *Global Change Biology* 14:2420-2431.

Timyan J. 1996. BWA YO: Important trees of Hati. Washington, D.C. South-East Consortium for International Development.

Torell LA, McDaniel KC. 1986. Optimal timing of investements to control honey mesquite. *Journal of Range Management* 39:378-382.

Van den Berg EC. 2010. Detection, quantification and monitoring Prosopis spp. in the Northern Cape Province of South Africa using Remote Sensing and GIS. MSc Thesis, North-West University, Potchefstroom.

van Klinken ED, Burwell CJ. 2005. Evidence from a gelechidd leaf-tier on mesquite (Mimosaceae: *Prosopis*) that semi-concealed Lepidopteran biological control agents may not be at risk from parasitism in Australian rangelands. *Biological control* 32:121-129.

van Klinken RD, Graham J, Flack LK. 2006. Population ecology of hybrid mesquite (*Prosopis* species) in Western Australia: how does it differ from native range invasions and what are the implications for impacts and management? *Biological Invasions* 8:727-741.

van Klinken RD, Shepherd D, Parr R, Robinson TP, Anderson L. 2007. Mapping mesquite (*Prosopis*) distribution and desnnity using visual areal surveys. *Rangeland Ecology and Management* 60:408-416.

van Klinken RD, White AJ. 2011. Overcoming seasonally fluctuating resources: Bruchid predation of mesquite (*Prosopis*) seed in dung. *Biological Control* 59:361-365.

van Klinken R. 2012. Prosopis spp. – mesquite. In: Julien M, McFadyen R, Cullen J, eds. Biological control of weeds in Australia. Melbourne, Australia: CSIRO.

van Wilgen BW, Forsyth GG, Le Maitre DC, Wannenburgh A, Kotze DF, van den Berg E, Henderson L. 2012. An assessment of the effectiveness of a large, national-scale invasive alien plant control strategy in South Africa. Biological Conservation 148:28-38.

Villagra PE, Boninsegna JA, Alvarez JA, Cony M, Cesca E, Villalba R. 2005. Dendroecology of *Prosoois flexuosa* woodlands in the Monte desert: Implications for their management. *Dendrochronologia* 22:209-213.

Walter K. 2011. *Prosopis*, an alien amoung the sacred trees of south India. Dissertation. Helsinki: University of Helsinki.

Warren A, Holechek J, Cardenas M. 1996. Honey mesquite influence on Chihuahuan desert vegetation. *Journal of Range Management* 49:46-52.

Wise RM, van Wilgen BW, Le Maitre DC. 2012. Costs, benefits and management options for an invasive alien tree species: The case of mesquite in the Northern Cape, South Africa. *Journal of Arid Environments* 84:80-90.

Wojtusik T, Felker P, Russel EJ. 1993. Cloning the erect, thornless, non-browsed nitrogen fixing trees of Haiti’s principal fuelwood species (*Prosopis juliflora*).*Agroferestry Systems* 21:293-300.

Zachariades C, Hofmann JH, Roberts A. 2011. Biological control of mesquite (Prosopis species) (Fabaceae) in South Africa. African Entomology 19:402-415.

Zimmermann HG. 1991. Biological control of Prosopis, *Prosopis* spp. (Fabaceae), in South Africa. *Agriculture, Ecosystems and Environment* 37:175-186.
